# SeuratExtend: streamlining single-cell RNA-seq analysis through an integrated and intuitive framework

**DOI:** 10.1093/gigascience/giaf076

**Published:** 2025-07-08

**Authors:** Yichao Hua, Linqian Weng, Fang Zhao, Florian Rambow

**Affiliations:** Department of Applied Computational Cancer Research, Institute for AI in Medicine (IKIM), University Hospital Essen, Essen 45131, Germany; University Duisburg-Essen, Essen 45141, Germany; Department of Oncology, KU Leuven, Leuven 3000, Belgium; University Duisburg-Essen, Essen 45141, Germany; Laboratory of Molecular Tumor Immunology, Department of Dermatology, University Hospital Essen, Essen 45147, Germany; Department of Applied Computational Cancer Research, Institute for AI in Medicine (IKIM), University Hospital Essen, Essen 45131, Germany; University Duisburg-Essen, Essen 45141, Germany; German Cancer Consortium (DKTK), partner site Essen/Düsseldorf, University Duisburg–Essen, University Medicine Essen, Essen 45141, Germany

**Keywords:** single-cell RNA-seq, bioinformatics, multitool integration, visualization, pathway analysis, R package, Seurat framework, education

## Abstract

Single-cell RNA sequencing (scRNA-seq) has revolutionized the study of cellular heterogeneity, but the rapid expansion of analytical tools has proven to be both a blessing and a curse, presenting researchers with significant challenges. Here, we present SeuratExtend, a comprehensive R package built upon the widely adopted Seurat framework, which streamlines scRNA-seq data analysis by strategically integrating essential tools and databases. SeuratExtend offers a user-friendly and intuitive interface for performing a wide range of analyses, including functional enrichment, trajectory inference, gene regulatory network reconstruction, and denoising. The package integrates multiple databases, such as Gene Ontology and Reactome, and incorporates popular Python tools like scVelo, Palantir, and SCENIC through a unified R interface. We illustrate SeuratExtend’s capabilities through case studies investigating tumor-associated high-endothelial venules and autoinflammatory diseases, as well as showcase its novel applications in pathway-level analysis and cluster annotation. SeuratExtend enhances data visualization with optimized plotting functions and carefully curated color schemes, ensuring both aesthetic appeal and scientific rigor. The package’s effectiveness has been demonstrated through successful workshops and training programs, establishing its value in both research and educational contexts. SeuratExtend empowers researchers to harness the full potential of scRNA-seq data, making complex analyses accessible to a wider audience. The package, along with comprehensive documentation, tutorials, and educational resources, is freely available at GitHub, providing a valuable resource for the single-cell genomics community.

## Introduction

In recent years, single-cell RNA sequencing (scRNA-seq) has revolutionized our understanding of cellular diversity and complexity at an unprecedented scale across various biological disciplines. The rapid advancement of this technology has led to an explosion of computational tools and algorithms, with over 1,700 methods reported as of April 2024 [1]. While this proliferation underscores the field’s vibrancy, it also presents a daunting challenge for researchers, who often find themselves overwhelmed by the myriad of choices and complexities.

The most common analytical tasks in scRNA-seq include doublet removal, denoising, batch integration, cell clustering and annotation, pathway and functional analysis, gene regulatory network inference, trajectory and pseudotime analysis, and cell–cell communication [2–5]. Each of these analytical dimensions addresses fundamental biological questions but often requires navigating a complex landscape of software tools with unique input requirements, operational intricacies, and output formats. This diversity, although scientifically enriching, frequently translates into steep learning curves. Researchers often encounter issues such as convoluted code, challenging error-tracing, outdated software, and ambiguous tutorials, leading to significant time wastage. Moreover, mainstream scRNA-seq analysis tools are primarily developed in either the R or Python languages, with additional options like Nextflow and Snakemake (RRID:SCR_003475) as workflow management systems. While R, with its core package Seurat (RRID:SCR_016341), and Python, with its central package Scanpy (RRID:SCR_018139), have their respective strengths [5, 6], researchers typically lack expertise in both languages, creating challenges in cross-platform tool interoperability. This challenge is compounded by the contrasting maturity of support ecosystems. Python’s scverse [7], an integrated ecosystem for single-cell analysis, provides a unified framework that simplifies tool access and workflow management. In contrast, the R ecosystem, despite having valuable resources like SeuratWrappers with its 17 methods, lacks a similarly comprehensive integration framework.

To address these challenges, we present SeuratExtend, a comprehensive and integrated R package designed to streamline scRNA-seq data analysis workflows. Built upon the widely adopted Seurat framework, SeuratExtend introduces 3 key innovations: it makes single-cell analyses more efficient and visually compelling; it bridges the gap between R and Python ecosystems, enabling access to powerful Python-based tools without requiring dual-language proficiency; and it pioneers novel methodological approaches, particularly pathway-level analysis that provides new perspectives on cellular heterogeneity.

These innovations are realized through 3 core design principles. *Integration* encompasses not only the incorporation of multiple databases (Gene Ontology [GO], Reactome) [8, 9] and Python tools, including scVelo (RRID:SCR_018168), CellRank (RRID:SCR_022827), Palantir, and SCENIC (RRID:SCR_017247) [10–13], but also the curation of existing R resources and educational materials, creating a comprehensive ecosystem that reduces learning barriers for newcomers. *Intuitive design* ensures that complex analyses become accessible through straightforward functions, detailed documentation, and interactive learning tools, including an artificial intelligence (AI)–powered chatbot. *Visual aesthetics* transform standard visualizations into publication-ready graphics through optimized plotting methods and professionally curated color schemes. By leveraging this philosophy, SeuratExtend has demonstrated exceptional value in both research applications and educational settings, as illustrated through diverse case studies, successful workshop implementations, and its growing user community.

## Materials and Methods

### Integration of multiple databases for GSEA

The GO database was obtained from the official website [14], including the .gaf and .obo files for both human and mouse. The .gaf files contain pathway-gene information, while the .obo files contain GO term naming, definitions, and hierarchical relationships between terms. The msga package’s readGAF function and ontologyIndex’s get_OBO were used to process the respective file formats. To construct the hierarchy, obsolete GO terms not present in the relational network were removed, and ontologyIndex was used to build standardized ontology_index objects for subsequent analyses.

For the Reactome database, relevant files were downloaded from the official website [15], including “Ensembl2Reactome_PE_All_Levels.txt” (containing pathway-gene information) and “Ensembl2Reactome_PE_Reactions.txt” (containing information on relationships between pathways). Human- and mouse-related pathways were extracted separately. As gene names were in Ensembl ID format, they were converted to gene symbols, and ontologyIndex was used to construct standardized ontology_index objects for subsequent analyses.

Other databases, such as Hallmark 50, KEGG, and BioCarta, were downloaded from GSEA database [16] and standardized into a uniform format. Cell-type marker gene information was obtained from the PanglaoDB database [17].

### Seurat object integration and conversion

The hdf5r package was used to create a dataset named “LOOM_SPEC_VERSION” in an HDF5 file, setting its value to “3.0.0” to specify the version of the Loom file format being used. The data type for this dataset was set to UTF-8 string. Count matrices (raw, normalized, or velocyto’s spliced/unspliced) were stored in the layers group, while meta.data information was stored in the col_attrs group. To convert from Loom to AnnData, Python’s Scanpy read_loom function was used.

### Python environment setup and integration of Python tools using conda and reticulate

The reticulate framework was used to integrate Python in R. By default, reticulate was used to create a conda environment named “seuratextend,” containing all potentially required packages. The conda environment was created and tested on Linux, macOS, and Windows systems to ensure compatibility and avoid version conflicts between Python packages. The environment specifications were saved as .yml files to allow conda to install an identical environment on the user’s respective operating system.

Visualizations for scVelo and CellRank were generated using reticulate to call the respective packages. For Palantir and MAGIC, reticulate was used to perform the computations, and the results were exported using numpy and imported into the Seurat object in R. SCENIC’s loom output was imported into the Seurat object using the LoomR package, creating an assay named “TF.” Various SeuratExtend visualization functions were then used to generate plots. For gene regulatory network visualizations, CytoScape was used in conjunction with SeuratExtend.

### Enhanced visualization

Visualization tools such as heatmaps, dimensional reduction plots, violin/box plots, cluster distribution plots, waterfall plots, and GSEA plots were built using the ggplot2 package, with layouts adjusted using cowplot. All statistical calculations were performed using the ggpubr package.

### Implementation of professional color schemes

The “color_pro” presets were constructed using the I Want Hue tool [18]. The provided API and JavaScript code were used to generate all presets.

### Gene identifier conversion

Gene naming conversions between human and mouse gene symbols and Ensembl IDs were performed using the BioMart database. The biomaRt package was utilized for conversions, with improvements in reliability and performance achieved by localizing the most commonly used databases, eliminating the need for Internet connectivity and addressing frequent instability issues with biomaRt. For UniProt ID conversion, the database was downloaded from the official website [19] and used for conversion to gene symbols.

## Results

### SeuratExtend: A comprehensive R ecosystem for single-cell analysis

Building upon the foundation laid by Seurat, SeuratExtend aims to create a more efficient and integrated workflow for single-cell RNA sequencing analysis in the R environment. While the Python ecosystem has benefited greatly from the comprehensive scverse project [7], which utilizes the universal AnnData format to connect various tools and algorithms, SeuratExtend takes a similar ecosystem-building approach for the R community, creating a cohesive analytical experience that bridges R and Python environments while maintaining the familiar Seurat workflow.

**Figure 1:**
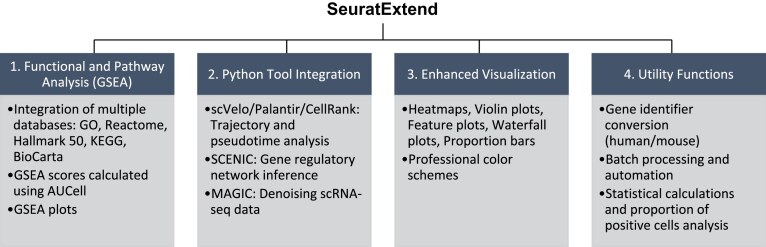
Overview of the SeuratExtend package’s key features. SeuratExtend streamlines single-cell RNA-seq data analysis by integrating essential components into the Seurat framework: (1) Functional and Pathway Analysis (GSEA) with multiple databases and AUCell algorithm; (2) Python Tool Integration for trajectory analysis (scVelo, Palantir, CellRank), gene regulatory network inference (SCENIC), and denoising (MAGIC); (3) Enhanced Visualization with optimized methods and professional color schemes; and (4) Utility Functions for gene identifier conversion, batch processing, and statistical analysis.

SeuratExtend expands upon the Seurat framework by integrating a comprehensive suite of tools and functionalities (Fig. 1). The package encompasses 4 key areas: (i) an advanced functional and pathway analysis module, which incorporates multiple databases and utilizes the AUCell algorithm for gene set enrichment analysis; (ii) seamless integration of Python-based tools, enabling sophisticated analyses such as trajectory inference, gene regulatory network reconstruction, and data denoising; (iii) enhanced visualization capabilities, featuring optimized plotting methods and professionally curated color schemes; and (iv) a collection of utility functions that streamline common tasks in single-cell analysis workflows. This integrated approach not only simplifies complex analytical processes but also bridges the gap between R and Python environments, providing researchers with a versatile toolkit for comprehensive single-cell RNA-seq data analysis.

The development of these capabilities reflects our strategic focus on combining essential functionality with user accessibility, an approach that distinguishes SeuratExtend within the broader landscape of single-cell analysis tools. To illustrate the unique position of SeuratExtend in the single-cell analysis toolkit landscape, we conducted a comprehensive comparison with other prominent tools and frameworks (Table 1). While comprehensive platforms like SCP [20] offer extensive tool integration, SeuratExtend takes a deliberately focused approach that emphasizes stability, maintainability, and accessibility. Compared to Seurat and SeuratWrappers, it offers a higher level of integration, providing a more seamless workflow from data input to final analysis and visualization. While SeuratWrappers includes 17 methods, only 3 are Python-native (Velocyto, scVelo, PaCMAP), and scVelo’s implementation requires users to write Python code for visualization and processing after conversion to h5ad format. In contrast, SeuratExtend uniquely bridges the gap between R and Python ecosystems by enabling direct use of Python tools within R. Unlike scverse, which primarily focuses on Python-based tools, SeuratExtend provides direct functions for converting between Seurat objects and AnnData format, facilitating interoperability between environments. This integration allows users to leverage powerful Python-based tools without needing to learn Python or switch between different platforms, making complex analytical methods accessible to researchers primarily working in R. These distinctive capabilities position SeuratExtend as a strategic choice for researchers seeking to navigate the increasingly complex landscape of single-cell analysis tools.

**Table 1:** Comparison of SeuratExtend with other single-cell analysis toolkits

Features	SeuratExtend	SCP	scverse	Seurat/SeuratWrappers	scRNA-tools
**Primary Language**	R	R	Python	R	Various
**Unified Data Structure**	✓ (Seurat)	✓ (Seurat)	✓ (AnnData)	✓ (Seurat)	✗
**R-Python Integration**	✓	✓	Limited	Limited	✗
**Comprehensive GSEA Integration**	✓	✓	Limited	Limited	Varies
**Enhanced Visualization**	✓ (ggplot)	✓ (ggplot)	Varies	Limited	Varies
**Learning Curve**	Moderate	Steep	Moderate	Moderate	Varies
**Integrated Tutorial**	✓	✓	✓	✓	Varies

It is important to emphasize that SeuratExtend is not merely a collection of existing tools. It introduces numerous extensions and novel developments, including enhanced database integration, improved visualization capabilities, and a suite of practical utility functions. Particularly noteworthy is SeuratExtend’s innovative approach to pathway-level analysis, which transforms how researchers can explore and interpret cellular heterogeneity beyond individual gene expression patterns. By enabling dimensionality reduction and clustering based on pathway enrichment scores rather than individual genes, SeuratExtend offers new insights into the functional characteristics of cell populations that may be difficult to discern through traditional gene-based methods. These innovations, combined with its integrative approach, position SeuratExtend as a powerful and unique resource in the R ecosystem for single-cell analysis. In the following sections, we will delve deeper into the key components and functionalities of SeuratExtend, demonstrating how it streamlines and enhances the single-cell RNA-seq analysis workflow.

### Integrated functional enrichment analysis with multisource databases

SeuratExtend offers an integrated approach to functional enrichment analysis, incorporating multiple authoritative databases within a unified framework (Fig. 1). This feature enables researchers to harness diverse data sources and employ state-of-the-art analytical methods, facilitating a comprehensive understanding of their single-cell transcriptomic data.

At the core of this integration lies the GO and Reactome databases. The GO database provides a structured representation of biological processes, molecular functions, and cellular components, enabling researchers to interpret their gene expression data within the context of well-established biological knowledge [8]. Complementing the GO database, SeuratExtend also integrates the Reactome knowledgebase, a comprehensive resource for curated biological pathways [9]. This integration allows researchers to explore the functional implications of their data within the context of well-described cellular processes, signaling cascades, and disease mechanisms. Furthermore, SeuratExtend incorporates a range of additional databases, including the Hallmark 50, KEGG, and BioCarta, providing researchers with an extensive collection of curated gene sets for their analyses.

Traditionally, functional enrichment analysis for bulk RNA-seq data involves identifying differentially expressed genes (DEGs) based on a predetermined cutoff and then comparing the resulting gene list against pathway databases to calculate enrichment scores. However, this approach suffers from inherent biases and limitations, as it fails to capture the nuances of single-cell data and relies on arbitrary cutoffs. SeuratExtend addresses this challenge by employing a dedicated single-cell-based method, AUCell, specifically designed to exploit the unique characteristics of single-cell RNA-sequencing data. Unlike bulk-sample-based methods, which can lead to biases, single-cell-based methods like AUCell are less susceptible to such issues. A comprehensive benchmarking study [21] evaluated various supervised signature-scoring methods, including ssGSEA, AUCell, Single Cell Signature Explorer (SCSE), and Jointly Assessing Signature Mean and Inferring Enrichment (JASMINE). The study concluded that while bulk-sample-based methods can introduce biases when applied to single-cell RNA-sequencing data, single-cell-based methods, such as AUCell and JASMINE, exhibit superior performance. Considering its adaptability, compatibility with downstream analyses (e.g., SCENIC), and widespread recognition within the scientific community, SeuratExtend employs AUCell as its primary algorithm for calculating gene set enrichment scores.

**Figure 2:**
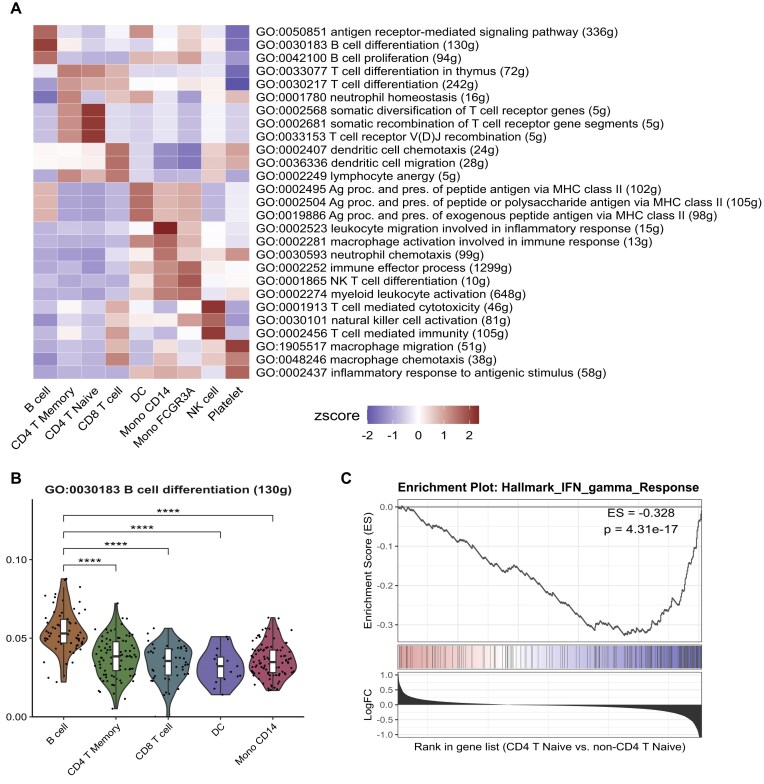
Visualizations of gene set enrichment analysis (GSEA) results using SeuratExtend. (A) Heatmap displaying the *z*-scores of immune-related GO terms across different cell types or conditions. (B) Violin plots showing the distribution of GSEA scores for the selected GO term across different cell types, with asterisks indicating statistically significant differences (****P < 0.001). (C) GSEA plot for the Hallmark IFN gamma Response gene set, comparing CD4 T naive cells to non-CD4 T naive cells. Kolmogorov–Smirnov test.

The integrated functional enrichment analysis in SeuratExtend is designed to be accessible and user-friendly. Researchers can easily navigate and search through the available databases, as well as filter pathways based on specific criteria (e.g., gene count, parent terms, or end-level terms). SeuratExtend constructs comprehensive hierarchical structures for the GO and Reactome databases, enabling researchers to perform targeted analyses on specific categories of interest, such as immune processes, metabolism, or signal transduction pathways, rather than analyzing the entire database in a nonspecific and computationally intensive manner. Researchers can perform GSEA on these specific categories or any other term from the GO hierarchy and visualize the results through informative heatmaps, violin plots, waterfall plots, or plots that emulate the GSEA plot developed by the Broad Institute (Fig. 2A–C). Additionally, SeuratExtend provides complementary functions to convert cryptic pathway identifiers into more interpretable names, further enhancing the user experience. This integration enables researchers to tap into the collective knowledge from multiple authoritative sources or custom gene sets, facilitating a deeper understanding of the biological processes underlying their single-cell transcriptomic data.

### Seamless integration of Python tools within an R environment

SeuratExtend offers integration of Python tools within the R environment, enabling researchers to utilize the analytical capabilities of both ecosystems. This integration is particularly valuable for tasks such as trajectory analysis, pseudotime calculation, gene regulatory network inference, and denoising, where Python tools like scVelo, Palantir, CellRank, SCENIC, and MAGIC excel [10–13, 22] (Fig. 1).

To facilitate this integration, SeuratExtend implements 3 key components: First, it redesigns the architecture for converting between fundamental scRNA-seq data storage formats, including Seurat, loom, and AnnData objects. Second, it provides a 1-stop solution for Python environment setup across Windows, macOS, and Linux platforms, freeing users from resolving environment conflicts between different tools. Third, it employs the reticulate framework to enable the execution of Python tools within R, eliminating the need for users to write Python code directly.

While the technical foundations of this integration are detailed in the Methods section, the key innovation lies in creating a seamless workflow between R and Python environments. SeuratExtend utilizes CRAN-sourced packages like hdf5r for stable data format conversion and leverages conda environments through reticulate to provide hassle-free Python dependency management. This approach eliminates common challenges faced by researchers attempting to use tools from both ecosystems, such as environment conflicts, format incompatibilities, and the need to write code in multiple languages.

**Figure 3:**
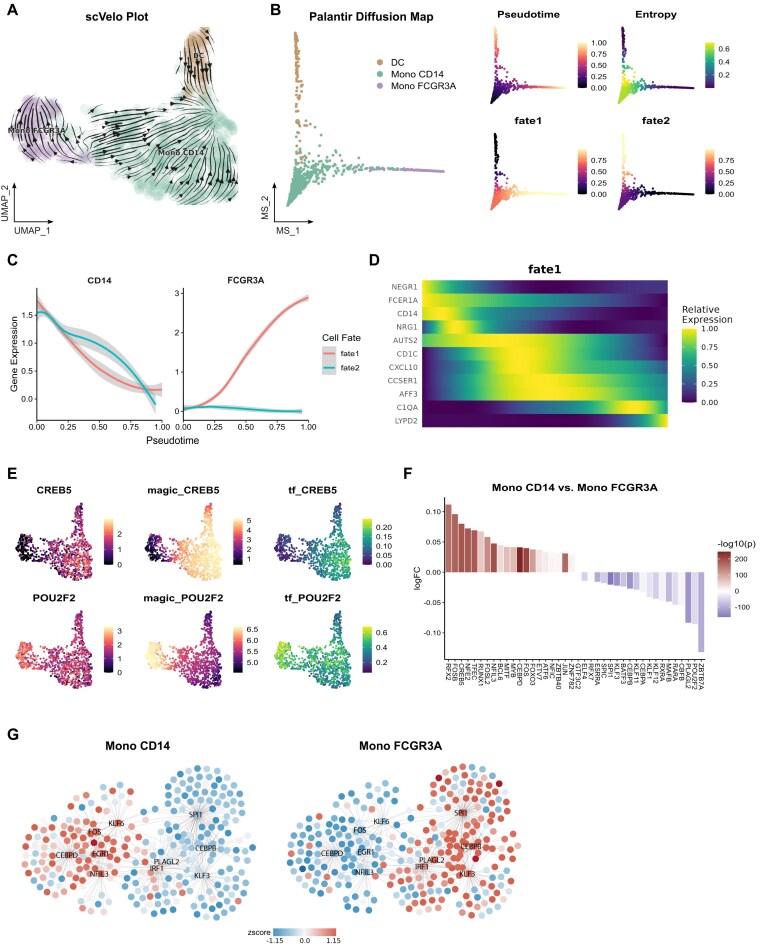
Trajectory analysis, pseudotime inference, denoising, and gene regulatory network visualization using SeuratExtend’s integrated Python tools. (A) RNA velocity analysis using scVelo, displaying velocity vectors on a UMAP embedding. (B) Diffusion map and pseudotime calculation using Palantir, comparing DC, Mono CD14, and Mono FCGR3A cell populations. (C) Gene expression dynamics along the pseudotime trajectory for the CD14 and FCGR3A genes. (D) Heatmap showing the relative expression of fate marker genes (rows) along the pseudotime trajectory (columns) for fate1. (E) UMAP visualizations of CREB5 and POU2F2 expression before (CREB5, POU2F2) and after (magic_CREB5, magic_POU2F2) denoising with MAGIC, as well as the transcription factor activity AUCell score (tf_CREB5, tf_POU2F2) inferred by SCENIC. (F) Waterfall plot highlighting differential TF regulon activities between Mono CD14 and Mono FCGR3A cell populations, with the top 20 TFs labeled. (G) Gene regulatory networks predicted by SCENIC for Mono CD14 and Mono FCGR3A cell populations, with nodes colored by relative gene expression (round nodes) or regulon activity (square nodes).

With these components in place, SeuratExtend unlocks a wide range of analytical possibilities. Users can employ the powerful trajectory analysis capabilities of scVelo and Palantir (Fig. 3A–D), which utilize RNA velocity and diffusion maps to predict cellular differentiation pathways and calculate pseudotime, respectively. These are highly regarded and popular tools for trajectory and pseudotime analysis. Additionally, CellRank offers an alternative approach to trajectory analysis, utilizing precalculated pseudotime to create informative trajectory plots. Furthermore, due to the sparse nature of scRNA-seq data, denoising techniques play a valuable role. Studies have compared various denoising algorithms [23] and identified MAGIC (Markov Affinity-based Graph Imputation of Cells) as a promising choice for improving clustering results (Fig. 3E). While the Rmagic package was previously available on CRAN but has since been removed due to inactivity, SeuratExtend integrates the use of MAGIC without relying on Rmagic.

Moreover, SeuratExtend integrates SCENIC, a comprehensive computational method for inferring gene regulatory networks from single-cell transcriptomic data. SeuratExtend can directly import pySCENIC-generated loom files into Seurat objects, enabling researchers to gain insights into the intricate regulatory mechanisms governing gene expression at the single-cell level (Fig. 3E–G).

This seamless integration of Python tools within the R environment allows researchers to harness the collective strengths of both ecosystems, enabling them to tackle complex analytical challenges and unravel the intricate mechanisms governing cellular processes at the single-cell level.

### Enhanced data visualization with aesthetic refinement

Effective visualization is paramount in the field of single-cell transcriptomics, enabling the communication of intricate cellular patterns, facilitating data interpretation, and conveying scientific insights. SeuratExtend recognizes the importance of aesthetics in scientific communication and introduces an array of tools to enhance the visual appeal and clarity of data representations.

While offering advanced analytical capabilities, SeuratExtend also places great emphasis on optimizing and expanding the fundamental visualization functions essential for presenting complex analyses. The package provides improved versions of core visualization tools such as heatmaps, dimensional reduction (Uniform Manifold Approximation and Projection [UMAP]) plots, violin/box plots, cluster proportion bar plots, dot/bubble plots, waterfall plots, and volcano plots (Figs. 1, 2A, 2B, 3E, and 3F). SeuratExtend also continues to expand its visualization capabilities in response to user needs. These enhancements streamline the visualization process and introduce new features and customization options, such as convenient statistical annotation and layout settings, enabling researchers to create more informative and visually compelling representations of their data.

Central to SeuratExtend’s approach to visualization is the implementation of thoughtfully curated color schemes that adhere to principles of effective data science plotting. The creation of the “professional discrete color” (*color_pro*) series stems from the understanding that choosing the right colors for scientific visualizations is critical. Colors must be distinct enough to differentiate data points clearly, yet coordinated and subdued enough to maintain professionalism and avoid visual strain.

**Figure 4:**
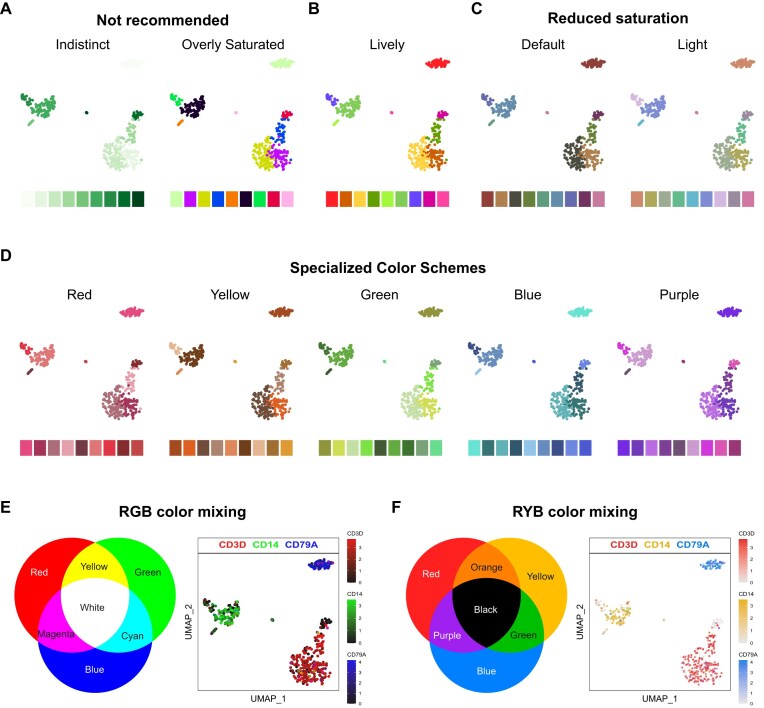
Comparison of color schemes and their suitability for data science visualization. (A) Not recommended color schemes: indistinct monochromatic colors that reduce visual distinction and overly saturated colors that can be visually aggressive and distracting. (B) A lively color scheme that, while engaging, may be considered informal for professional journal standards. (C) The “color_pro” series offers 2 meticulously crafted color schemes: “default” and “light,” which span the entire hue domain and cater to general use cases. (D) The “color_pro” series also includes 5 specialized color schemes: “red,” “yellow,” “green,” “blue,” and “purple,” which offer harmonious hues confined to specific regions, enabling vibrant yet coordinated visualizations that reflect the biological or categorical properties of the data. (E, F) Multifeature visualization using color mixing principles in the RGB (E) or RYB (F) color system. The expression levels of 3 genes (CD3D, CD14, and CD79A) are displayed on a UMAP plot.

In the realm of data science visualization, certain color choices should be avoided, such as monochromatic schemes that can reduce visual distinction (Fig. 4A), causing data points to blend together. Similarly, overly saturated colors can be visually aggressive and distracting, detracting from the scientific message (Fig. 4A). While certain vibrant schemes might be engaging in an advertising context, they may be considered informal for professional journal standards (Fig. 4B).

The *color_pro* series addresses these concerns by offering a collection of 7 meticulously crafted color schemes: *default, light, red, yellow, green, blue*, and *purple* (Fig. 4C, D). These palettes are generated using *I Want Hue* [18], an optimized algorithm that ensures visually pleasing and distinctly separable color combinations, with carefully adjusted parameters to align with the aforementioned principles. The *default* and *light* schemes span the entire hue domain, catering to general use cases while accommodating different background and text color requirements (Fig. 4C). The specialized color schemes (*red, yellow, green, blue*, and *purple*) offer harmonious hues confined to specific regions (Fig. 4D), enabling vibrant yet coordinated visualizations that reflect the biological or categorical properties of the data.

In addition to the curated color schemes, SeuratExtend introduces an innovative approach to visualize multiple features simultaneously on a single dimension reduction plot, such as UMAP. This novel method utilizes the principles of color mixing in either the RGB (red, green, blue) or RYB (red, yellow, blue) color systems to represent the levels of different features, such as gene expression, pathway AUCell scores, or transcription factor activities.

In the RGB system, black represents no or low expression, while brighter colors indicate higher levels (Fig. 4E). This color mixing approach is straightforward and well established in the digital color space. In contrast, the RYB system, which is more intuitive and closely mimics the subtractive color mixing used in traditional art, employs white to represent no expression, with deeper colors indicating higher expression levels (Fig. 4F).

However, designing an algorithm for RYB color mixing that accurately simulates real-world color blending poses a challenge. Moreover, the pure primary colors in the RYB system have inherent limitations for data visualization purposes, as yellow tends to be too light and blue too dark. To address these issues, SeuratExtend has developed a custom algorithm for RYB color mixing and has fine-tuned the brightness and saturation of the primary colors to optimize them for data visualization (Fig. 4F).

In summary, SeuratExtend equips researchers to create visually compelling and scientifically rigorous representations of complex biological insights by integrating innovative color schemes, multifeature visualization capabilities, and intuitive color mixing principles into its optimized plotting tools.

### Utility toolset: streamlining analysis workflows

SeuratExtend introduces a comprehensive suite of utility tools designed to streamline and enhance scRNA-seq data analysis workflows (Fig. 1). A recurring challenge in scRNA-seq analysis involves reconciling gene identifiers across disparate databases and organisms. SeuratExtend addresses this by providing robust functions that facilitate gene naming conversions between human and mouse gene symbols, Ensembl IDs, and UniProt accession numbers. These functions utilize localized databases, ensuring reliable and efficient conversion while mitigating instability issues associated with online resources. Users can directly convert gene expression matrices between human and mouse counterparts, streamlining cross-species analyses. For scenarios requiring online resources, SeuratExtend provides flexibility to fetch results directly from BioMart databases.

SeuratExtend introduces convenient tools for computing statistics and assessing the proportion of positive cells within clusters or groups. One tool computes various metrics, including mean, median, *z*-scores, or log-fold changes, for genomic data stored in Seurat objects or standard matrices. This enables researchers to identify cluster-specific gene expression patterns or pathway activities, crucial for understanding cellular heterogeneity and functional characteristics. Another tool allows users to assess the proportion of positive cells expressing a particular feature within specified clusters or groups, enabling the identification of genes or pathways exhibiting significant expression levels within subpopulations of cells.

To further enhance accessibility and reproducibility, SeuratExtend introduces a function that automates the execution of a standard Seurat pipeline, including normalization, principal component analysis (PCA), clustering, and UMAP visualization. This function offers extensive customization options and intelligent conditional execution, ensuring that specific steps are rerun only when necessary. Additionally, it provides the option to integrate and correct for batch effects using the Harmony algorithm. According to benchmarking studies [24], Harmony performs well in general tasks, offering fast computation and efficient resource utilization while maintaining simplicity of use, making it an excellent choice for batch effect correction in scRNA-seq data analysis.

SeuratExtend’s utility toolset represents a valuable advancement in streamlining and enhancing scRNA-seq data analysis workflows, facilitating efficient navigation of the complexities of scRNA-seq data exploration and accelerating scientific discoveries.

### Novel applications of seuratextend in pathway-level analysis and cluster annotation

The SeuratExtend framework offers innovative approaches to address fundamental challenges in single-cell transcriptomic data analysis, such as cellular heterogeneity interpretation and cluster annotation. By leveraging the package’s comprehensive integration of pathway databases and analytical tools, researchers can explore novel strategies to gain deeper insights into the functional characteristics of cell populations and streamline the annotation process.

#### Exploring and analyzing single-cell data at the pathway level

SeuratExtend introduces a novel approach to dimensionality reduction and clustering by harnessing its extensive integration of pathway databases, such as GO, Reactome, Kyoto Encyclopedia of Genes and Genomes (KEGG), and BioCarta. Instead of relying solely on gene expression–cell matrices, this method transforms the data into pathway enrichment score–cell matrices using AUCell. Subsequent analyses, including PCA and clustering, are then performed on these pathway-level matrices. This pathway-based approach offers several advantages. First, clustering based on pathway-level information rather than individual genes greatly reduces the impact of gene expression fluctuations. Second, by focusing on curated pathways containing functionally characterized genes, the influence of sample-specific genes with unknown functions, such as noncoding RNAs and pseudogenes, is minimized.

**Figure 5:**
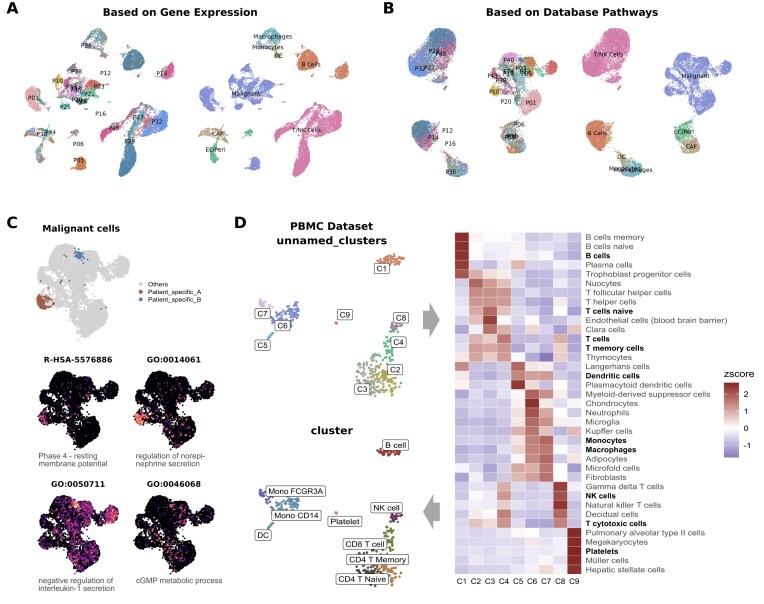
SeuratExtend’s novel applications in pathway-level analysis and cluster annotation. (A) UMAP visualization of melanoma scRNA-seq data colored by sample origin and clusters, demonstrating the influence of batch effects on gene expression–based clustering. (B) UMAP visualization of the same data colored by sample origin and clusters derived from pathway enrichment scores based on multiple databases (GO, Reactome, KEGG, and BioCarta), showing the grouping of cells with conserved features (e.g., T cells and B cells) while preserving biological variations in malignant cells. (C) UMAP visualization of malignant cells highlighting the location of patient_specific_A and patient_specific_B clusters (top) and their corresponding specific pathway activities (bottom). (D) UMAP visualization of unsupervised clustering results yielding 9 unnamed clusters (top left) and the corresponding heatmap of top enriched cell type signatures from PanglaoDB for each cluster (right), facilitating semi-automated cluster annotation (bottom left).

To illustrate the potential of this approach, we present an example using scRNA-seq data from a melanoma cohort [25]. When clustering is based on gene expression, the UMAP visualization is significantly influenced by sample origin (Fig. 5A). In contrast, clustering based on pathway enrichment scores derived from multiple databases (GO, Reactome, KEGG, and BioCarta) effectively aggregates cells with conserved features, such as T cells and B cells, while preserving the biological variations in malignant cells across samples (Fig. 5B). Importantly, this pathway-based analysis can provide novel functional insights that may be difficult to obtain through traditional gene-based methods. For instance, in the original publication of the melanoma dataset, the authors identified 2 clusters of malignant cells named “patient_specific_A” and “patient_specific_B” but could not infer their functional characteristics based on marker genes alone. By leveraging SeuratExtend’s pathway-based approach, we can easily identify the specific pathways that distinguish these clusters (Fig. 5C), thereby gaining a deeper understanding of their biological roles.

SeuratExtend’s pathway-based approach is highly versatile, as it can be applied to various databases, as well as customized pathway databases, enabling fine-tuned clustering for specific datasets. However, it should be acknowledged that this method shares some of AUCell’s limitations, such as sensitivity to the number of genes expressed per cell (nGene) and optimal performance within certain gene set size ranges. Consequently, substantial differences in nGene across samples may impact the computational results.

#### Semi-automated cluster annotation with signature enrichment analysis

SeuratExtend also facilitates semi-automated cluster annotation without the need for additional tools like SingleR [26]. By leveraging databases such as PanglaoDB [27], which contains marker genes for over 100 cell types, researchers can calculate AUCell scores to identify enriched cell-type signatures within each cluster, greatly assisting the annotation process.

In the provided example, unsupervised clustering yields 9 unnamed clusters (Fig. 5D). By calculating AUCell scores using PanglaoDB and sorting the top cell-type signatures for each cluster, researchers can efficiently annotate the clusters based on this information (Fig. 5D).

In summary, SeuratExtend’s novel applications in pathway-level analysis and cluster annotation demonstrate its potential to address critical challenges in scRNA-seq data interpretation. By leveraging integrated pathway databases and signature enrichment analysis, SeuratExtend enables researchers to gain deeper insights into the functional characteristics of cell populations, streamline the annotation process, and ultimately expand the analytical horizons of single-cell transcriptomics.

### Educational impact

We recently demonstrated SeuratExtend’s educational value by designing a comprehensive scRNA-seq analysis teaching module based on it. The course successfully transitioned complete beginners to competent analysts and underlines SeuratExtend’s accessibility and intuitive design. Structured as 6 progressive lessons, the curriculum began with fundamental R programming concepts and gradually advanced to sophisticated analytical techniques. This systematic approach enabled participants to build confidence with basic operations before tackling more complex analyses. Notably, the course expanded beyond SeuratExtend’s core functionality to cover essential advanced topics in single-cell analysis, including doublet removal, cell–cell communication analysis, automated cluster annotation, T-cell receptor (TCR)/B-cell receptor (BCR) analysis analysis, and copy number variation inference. This comprehensive coverage ensured participants to gain a deeper understanding of the single-cell analysis landscape while learning to effectively utilize SeuratExtend’s streamlined workflow.

Following the workshop’s success and responding to community interest, all course materials have been made freely available online, extending SeuratExtend’s educational impact beyond the initial workshop. This development marks a significant step in democratizing single-cell analysis education, making sophisticated analytical techniques accessible to a broader audience of researchers while positioning SeuratExtend as a valuable educational resource in the field of single-cell genomics.

### Comprehensive tutorials and interactive learning resources

SeuratExtend prioritizes user accessibility through its extensive documentation and interactive learning resources. The package’s primary documentation [28] provides detailed function descriptions, practical examples, and comprehensive workflow guides. This documentation is continuously refined based on user feedback and common questions, ensuring its relevance and utility for users at all skill levels.

To further enhance the learning experience, SeuratExtend incorporates artificial intelligence through a specialized chatbot powered by large language models. This interactive assistant, trained on the package’s documentation and tutorials, provides real-time support for users encountering technical challenges or seeking guidance on analytical approaches. The chatbot complements traditional documentation by offering personalized assistance and helping users navigate the complexities of single-cell analysis.

The integration of these educational resources—comprehensive documentation, interactive support, and structured tutorials—creates a robust learning ecosystem that supports users from their initial encounter with single-cell analysis through to advanced applications. This systematic approach to user education and support distinguishes SeuratExtend within the single-cell analysis toolkit landscape, making sophisticated analytical techniques more accessible to researchers across different experience levels.

### Evolving applications of SeuratExtend: From early adoption to current capabilities

SeuratExtend originated from practical research needs, with its capabilities expanding through real-world application. Here, we highlight specific instances where the package facilitated discoveries in 2 diverse research domains, demonstrating its practical value and subsequent evolution.

In a study investigating tumor-associated high-endothelial venules (TU-HEVs) [29], SeuratExtend’s integrated workflow enabled a comprehensive multiangle analysis approach that would have been challenging to implement with fragmented tools. The researchers conducted extensive comparative analyses between TU-HEVs, lymph node HEVs (LN-HEVs), and tumor ECs (TU-ECs) across both murine and human samples. This comprehensive analysis integrated multiple trajectory inference methods in Python and R, pathway enrichment analyses, and transcription factor network reconstruction within a unified framework. The ability to seamlessly transition between these analytical perspectives was crucial for identifying the most promising directions for experimental validation, ultimately confirming that TU-HEVs arise from the metaplastic conversion of postcapillary venules (PCVs). Similarly, in a subsequent study of systemic autoinflammatory diseases (SAIDs) [30], this integrated approach facilitated rapid cross-disciplinary analysis, combining GSEA with trajectory analysis tools like scVelo and Palantir to quickly uncover an unexpected therapeutic mechanism. Rather than simply suppressing inflammatory cytokines, anti-TNF therapy was found to alter macrophage differentiation pathways—an insight that emerged from the ability to efficiently investigate multiple analytical perspectives in parallel.

These applications demonstrate how SeuratExtend’s integration of diverse analytical methods within a unified framework can facilitate biological discoveries by enabling more comprehensive data exploration. Since these initial applications, SeuratExtend has undergone significant development based on user feedback. Many functions have been optimized for computational efficiency and compatibility with different R environments. The current version represents a substantial advancement, offering a comprehensive, integrated platform for single-cell RNA-seq analysis, supported by extensive documentation and tutorials to lower the entry barrier for researchers new to the field.

## Discussion

SeuratExtend represents a significant advancement in the field of scRNA-seq analysis, addressing the critical challenges that have emerged from the rapid proliferation of computational tools and algorithms in this domain. Our comprehensive evaluation and integration of essential analytical components have resulted in a robust, user-friendly framework that streamlines complex workflows and enhances the accessibility of advanced scRNA-seq analysis techniques.

The development of SeuratExtend was guided by 3 core principles: integration, intuitive design, and visual aesthetics. By incorporating multiple databases, analytical tools, and visualization techniques, we have created a cohesive ecosystem that bridges the gap between R and Python environments. This integration not only simplifies the analytical process but also expands the repertoire of available tools for researchers working primarily in R. The intuitive design of SeuratExtend, featuring straightforward functions and extensive documentation, significantly lowers the entry barrier for both novice and experienced users. Furthermore, our emphasis on visual aesthetics, exemplified by the carefully curated color schemes and optimized visualization methods, enhances the clarity and impact of data representation in scientific communications.

SeuratExtend provides a comprehensive suite of analytical tools, encompassing essential components such as denoising, batch integration, pathway analysis, gene regulatory network inference, and trajectory analysis. While the single-cell field continues to expand with new methodologies and tools, SeuratExtend adopts a strategic approach to tool integration, recognizing that reimplementing every available method within a single package is neither practical nor necessarily beneficial to users. Many existing tools already offer excellent Seurat compatibility, making redundant integration unnecessary. Instead, SeuratExtend focuses on ensuring robust implementation and reliable maintenance of its integrated functionalities while providing comprehensive educational resources and guidance for additional analytical approaches such as doublet removal, cell–cell communication, copy number variation inference, and automated cluster annotation through its extensive tutorial system. This approach ensures that users not only have access to well-maintained analytical tools but also gain the knowledge and confidence to effectively utilize the broader ecosystem of single-cell analysis methods.

To realize this potential, SeuratExtend’s future development and expansion will be greatly enhanced by active community engagement. As with many successful open-source projects, user feedback and contributions from the scientific community will play a crucial role in shaping the package’s trajectory. This collaborative approach ensures that SeuratExtend continues to evolve in alignment with users’ needs and the rapidly advancing field of single-cell genomics. We encourage users to provide feedback, suggest new features, and contribute to the codebase, fostering a vibrant ecosystem that can adapt to emerging challenges and opportunities in scRNA-seq analysis.

Looking ahead, SeuratExtend’s integration and intuitive nature position it as an excellent educational resource, further lowering the entry barrier for those eager to learn single-cell analysis. Additionally, the rise of large language models (LLMs) presents an opportunity for AI-assisted education. While SeuratExtend currently uses OpenAI’s platform for a chatbot, future endeavors may involve building more versatile chatbots using frameworks like Langchain and other LLMs like Claude and Llama 3, increasing accessibility and reducing costs. Furthermore, SeuratExtend’s standardized data analysis and visualization framework could pave the way for visual applications like Shiny apps, making scRNA-seq analysis accessible even to non-bioinformaticians.

In conclusion, SeuratExtend represents a significant stride in streamlining scRNA-seq data analysis, offering a focused and integrated solution built upon the Seurat framework. By addressing the challenges of tool proliferation and complexity while maintaining a clear emphasis on stability and user accessibility, SeuratExtend has made advanced scRNA-seq analysis more accessible to a broader range of researchers. The package’s success in educational settings and positive user feedback demonstrate its effectiveness in bridging the gap between sophisticated analytical capabilities and practical usability.

## Availability of Source Code and Requirements

Project name: SeuratExtend

Project homepage: https://github.com/huayc09/SeuratExtend

Operating system(s): Linux/Windows/MacOS

Programming language: R

Other requirements: R (≥ 3.6), Seurat, dplyr, ggplot2, reticulate

License: GNU General Public License (GPL) version 3 or later. Data files (*.rda files) are released under CC0 1.0 Universal Public Domain Dedication.

RRID:SCR_026143

WorkflowHub [31]: 10.48546/workflowhub.workflow.1385.1

Software Heritage PID [32]: swh:1:snp:815e826cdf8c4f4985308551b779b82e45895c81;origin=https://github.com/huayc09/SeuratExtend

## Supplementary Material

giaf076_Authors_Response_To_Reviewer_Comments_Original_Submission

giaf076_GIGA-D-24-00558_Original_submission

giaf076_GIGA-D-24-00558_Revision_1

giaf076_Reviewer_1_Report_Original_Submission Yu H. Sun, Ph.D. -- 1/25/2025

giaf076_Reviewer_2_Report_Original_Submission Daniel A. Skelly -- 2/16/2025

giaf076_Reviewer_2_Report_Revision_1 Daniel A. Skelly -- 5/27/2025

## Data Availability

The SeuratExtend package is freely available in the GitHub repository (https://github.com/huayc09/SeuratExtend) and has been deposited to Figshare [33]. A version of record snapshot of the GitHub repository has been archived in the Software Heritage Library with the PID swh:1:snp:815e826cdf8c4f4985308551b779b82e45895c81;origin (https://github.com/huayc09/SeuratExtend) [32]. The repository also contains comprehensive documentation and tutorials (https://huayc09.github.io/SeuratExtend/) to help users get started with the package and understand its functionalities. The example datasets used in the tutorials are available on Zenodo [34]. SeuratExtend has been registered in WorkflowHub [31]. SeuratExtend Chatbot (beta version, powered by ChatGPT): https://chatgpt.com/g/g-8scQjmzkd-scrna-seq-assistant

## Abbreviations

BCR: B-cell receptor; DEGs: differentially expressed genes; GO: Gene Ontology; GSEA: gene set enrichment analysis; MAGIC: Markov Affinity-based Graph Imputation of Cells; PCA: principal component analysis; SCENIC: Single-Cell Regulatory Network Inference and Clustering; scRNA-seq: single-cell RNA sequencing; TCR: T-cell receptor; UMAP: Uniform Manifold Approximation and Projection.

## References

[1] Zappia L, Theis FJ. Over 1000 tools reveal trends in the single-cell RNA-seq analysis landscape. Genome Biol. 2021;22:301. 10.1186/s13059-021-02519-4. 34715899 PMC8555270

[2] Luecken MD, Theis FJ. Current best practices in single-cell RNA-seq analysis: a tutorial. Mol Syst Biol. 2019;15:e8746. 10.15252/msb.20188746. 31217225 PMC6582955

[3] Heumos L, Schaar AC, Lance C, et al. Best practices for single-cell analysis across modalities. Nat Rev Genet. 2023;24:550–72. 10.1038/s41576-023-00586-w. 37002403 PMC10066026

[4] Kharchenko PV . The triumphs and limitations of computational methods for scRNA-seq. Nat Methods. 2021;18:723–32. 10.1038/s41592-021-01171-x. 34155396

[5] Hao Y, Hao S, Andersen-Nissen E, et al. Integrated analysis of multimodal single-cell data. Cell. 2021;184:3573–87.e29. 10.1016/j.cell.2021.04.048. 34062119 PMC8238499

[6] Wolf FA, Angerer P, Theis FJ. SCANPY: large-scale single-cell gene expression data analysis. Genome Biol. 2018;19:15. 10.1186/s13059-017-1382-0. 29409532 PMC5802054

[7] Virshup I, Bredikhin D, Heumos L, et al. The scverse project provides a computational ecosystem for single-cell omics data analysis. Nat Biotechnol. 2023;41:604–6. 10.1038/s41587-023-01733-8. 37037904

[8] The Gene Ontology Consortium . The gene ontology Resource: 20 years and still GOing strong. Nucleic Acids Res. 2019;47:D330–38. 10.1093/nar/gky1055. 30395331 PMC6323945

[9] Gillespie M, Jassal B, Stephan R, et al. The reactome pathway knowledgebase 2022. Nucleic Acids Res. 2022;50:D687–92. 10.1093/nar/gkab1028. 34788843 PMC8689983

[10] Bergen V, Lange M, Peidli S, et al. Generalizing RNA velocity to transient cell states through dynamical modeling. Nat Biotechnol. 2020;38:1408–14. 10.1038/s41587-020-0591-3. 32747759

[11] Lange M, Bergen V, Klein M, et al. CellRank for directed single-cell fate mapping. Nat Methods. 2022;19:159–70. 10.1038/s41592-021-01346-6. 35027767 PMC8828480

[12] Setty M, Kiseliovas V, Levine J, et al. Characterization of cell fate probabilities in single-cell data with Palantir. Nat Biotechnol. 2019;37:451–60. 10.1038/s41587-019-0068-4. 30899105 PMC7549125

[13] Aibar S, González-Blas CB, Moerman T, et al. SCENIC: single-cell regulatory network inference and clustering. Nat Methods. 2017;14:1083–86. 10.1038/nmeth.4463. 28991892 PMC5937676

[14] The Gene Ontology Consortium . Gene Ontology Database. https://geneontology.org/. Accessed 7 December 2024.

[15] Reactome. Reactome Pathway Database. https://reactome.org/. Accessed 7 December 2024.

[16] GSEA Molecular Signatures Database. https://www.gsea-msigdb.org/. Accessed 7 December 2024.

[17] Franzén O, Gan LM, Björkegren JLM. PanglaoDB Database. https://panglaodb.se/. Accessed 7 December 2024. 10.1093/database/baz046 PMC6450036 30951143

[18] MediaLab. I Want Hue Color Tool. http://medialab.github.io/iwanthue/. Accessed 7 December 2024.

[19] UniProt Consortium . UniProt Database. https://www.uniprot.org/. Accessed 7 December 2024.

[20] Zhang H . SCP: Single Cell Pipeline (Version 0.5.6). https://github.com/zhanghao-njmu/scp. Accessed 7 December 2024.

[21] Noureen N, Ye Z, Chen Y, et al. Signature-scoring methods developed for bulk samples are not adequate for cancer single-cell RNA sequencing data. eLife. 2022;11:e71994. 10.7554/eLife.71994. 35212622 PMC8916770

[22] van Dijk D, Sharma R, Nainys J, et al. Recovering gene interactions from single-cell data using data diffusion. Cell. 2018;174:716–29.e27. 10.1016/j.cell.2018.05.061. 29961576 PMC6771278

[23] Galuzzi BG, Vanoni M, Damiani C. Combining denoising of RNA-seq data and flux balance analysis for cluster analysis of single cells. BMC Bioinf. 2022;23:445. 10.1186/s12859-022-04967-6. PMC9597960 36284276

[24] Yu X, Xu X, Zhang J, et al. Batch alignment of single-cell transcriptomics data using deep metric learning. Nat Commun. 2023;14:960. 10.1038/s41467-023-36635-5. 36810607 PMC9944958

[25] Pozniak J, Pedri D, Landeloos E, et al. A TCF4-dependent gene regulatory network confers resistance to immunotherapy in melanoma. Cell. 2024;187:166–83.e25. 10.1016/j.cell.2023.11.037. 38181739

[26] Aran D, Lun A, Bunis D, et al. SingleR. Bioconductor. 10.18129/B9.bioc.SingleR.Accessed 7 December 2024.

[27] Franzén O, Gan L-M, Björkegren JLM. PanglaoDB: a web server for exploration of mouse and human single-cell RNA sequencing data. Database (Oxford). 2019;2019:baz046 10.1093/database/baz046. 30951143 PMC6450036

[28] Hua Y . SeuratExtend documentation and tutorials. https://huayc09.github.io/SeuratExtend/. Accessed 7 December 2024.

[29] Hua Y, Vella G, Rambow F, et al. Cancer immunotherapies transition endothelial cells into HEVs that generate TCF1+ T lymphocyte niches through a feed-forward loop. Cancer Cell. 2022;40:1600–18.e10. 10.1016/j.ccell.2022.11.002. 36423635 PMC9899876

[30] Hua Y, Wu N, Miao J, et al. Single-cell transcriptomic analysis in two patients with rare systemic autoinflammatory diseases treated with anti-TNF therapy. Front Immunol. 2023;14:1091336. 10.3389/fimmu.2023.1091336. 36911721 PMC9998688

[31] Hua Y . SeuratExtend. WorkflowHub. 2025. 10.48546/WORKFLOWHUB.WORKFLOW.1385.1.Accessed 30 May 2025.

[32] Hua Y, Weng L, Zhao F, et al. SeuratExtend: an enhanced toolkit for scRNA-seq analysis (Version 1) [Computer software]. Software Heritage. 2025. https://archive.softwareheritage.org/swh:1:snp:815e826cdf8c4f4985308551b779b82e45895c81;origin=https://github.com/huayc09/SeuratExtend.Accessed 30 May 2025.

[33] Hua Y, Weng L, Zhao F, et al. SeuratExtend (Version 1) [Computer software]. Figshare. 2025. 10.6084/m9.figshare.26264255.v1. Accessed 30 May 2025.

[34] Hua Y, Weng L, Zhao F, et al. SeuratExtend tutorial: curated example datasets for single-cell analysis. Zenodo. 2025. 10.5281/zenodo.10944065.Accessed 30 May, 2025.

