# Supplementary material for: SeuratExtend: streamlining single-cell RNA-seq analysis through an integrated and intuitive framework
Source: Gigascience. 2025 Jul 8;14:giaf076. doi: 10.1093/gigascience/giaf076 (PMC12236070; doi:10.1093/gigascience/giaf076)
Supplement: giaf076_GIGA-D-24-00558_Revision_1 [file giaf076_giga-d-24-00558_revision_1.pdf]

GigaScience

SeuratExtend: Streamlining Single-Cell RNA-Seq Analysis Through an Integrated and Intuitive Framework

--Manuscript Draft--

**Manuscript Number:**	GIGA-D-24-00558R1
**Full Title:**	SeuratExtend: Streamlining Single-Cell RNA-Seq Analysis Through an Integrated and Intuitive Framework
**Article Type:**	Technical Note
**Funding Information:**	Melanoma Research Alliance — Dr. Yichao Hua, Prof. Florian Rambow<br>Wolfgang & Gertrud Boettcher Foundation — Prof. Florian Rambow<br>Else Kröner-Fresenius-Stiftung (2021_EKMK.12) — Dr. Fang Zhao
**Abstract:**	Single-cell RNA sequencing (scRNA-seq) has revolutionized the study of cellular heterogeneity, but the rapid expansion of analytical tools has proven to be both a blessing and a curse, presenting researchers with significant challenges. Here, we present SeuratExtend, a comprehensive R package built upon the widely adopted Seurat framework, which streamlines scRNA-seq data analysis by strategically integrating essential tools and databases. SeuratExtend offers a user-friendly and intuitive interface for performing a wide range of analyses, including functional enrichment, trajectory inference, gene regulatory network reconstruction, and denoising. The package integrates multiple databases, such as Gene Ontology and Reactome, and incorporates popular Python tools like scVelo, Palantir, and SCENIC through a unified R interface. We illustrate SeuratExtend's capabilities through case studies investigating tumor-associated high-endothelial venules and autoinflammatory diseases, and showcase its novel applications in pathway-level analysis and cluster annotation. SeuratExtend enhances data visualization with optimized plotting functions and carefully curated color schemes, ensuring both aesthetic appeal and scientific rigor. The package's effectiveness has been demonstrated through successful workshops and training programs, establishing its value in both research and educational contexts. SeuratExtend empowers researchers to harness the full potential of scRNA-seq data, making complex analyses accessible to a wider audience. The package, along with comprehensive documentation, tutorials, and educational resources, is freely available at GitHub, providing a valuable resource for the single-cell genomics community.
**Corresponding Author:**	Yichao Hua, M.D. Ph.D.<br>University Hospital Essen: Universitatsklinikum Essen<br>Essen, GERMANY
**Corresponding Author Secondary Information:**	
**Corresponding Author's Institution:**	University Hospital Essen: Universitatsklinikum Essen
**Corresponding Author's Secondary Institution:**	
**First Author:**	Yichao Hua, M.D. Ph.D.
**First Author Secondary Information:**	
**Order of Authors:**	Yichao Hua, M.D. Ph.D.<br>Linqian Weng, M.D.<br>Fang Zhao, Ph.D.<br>Florian Rambow, Ph.D.
**Order of Authors Secondary Information:**	

Powered by Editorial Manager® and ProduXion Manager® from Aries Systems Corporation

Response to Reviewers:	Reviewer reports:<br>Reviewer #1: This manuscript introduces an extended version of the widely-used Seurat package, named SeuratExtend. Specifically, Hua et al. developed an integrated an intuitive framework to streamline scRNA-seq data analysis, such as trajectory analysis, GRN construction, and functional enrichment analysis. The package also features direct integration with other popular tools, including Seurat, scVelo, etc. Notably, the software has been demonstrated through training programs, with over 100 stars on GitHub, which is impressive. I have tested the package, including installation and some basic functions. Moreover, the GitHub webpage is well-documented, featuring multiple use cases tailored for beginners. The overall user experience exceeded my expectations, though I have a few minor comments for improvement:<br>1, The DimPlot2 function is very useful, and easy to customize the colors. However, the default color scheme seems to be too dark. Considering a more distinguishable and visually appealing color palette might be a solution.<br><br>RESPONSE:<br>We appreciate the reviewer's valuable feedback regarding the DimPlot2 function's default color scheme. This observation aligns with feedback from multiple users who found the default "dark" palette too intense for certain visualization contexts.<br>In response to this feedback, we have implemented several significant improvements in SeuratExtend v1.2.0:<br>·We have changed the default color scheme for discrete variables from "default" (dark) to "light" - a more visually comfortable palette with increased brightness and reduced saturation. This change makes plots more readable and visually appealing, particularly when labels are displayed.<br>·We have introduced a new "bright" color scheme that offers higher contrast colors for visualizations requiring more distinct separation between groups. This option is particularly useful for presentations or when subtle color differences need to be emphasized.<br>·We've expanded the maximum number of colors supported by our color palettes from 50 to 80, accommodating datasets with larger numbers of clusters or categories.<br>These enhancements provide users with greater flexibility in choosing color schemes that best suit their specific visualization needs while addressing the reviewer's concerns about distinguishability and visual appeal. The changes have been fully documented in our package vignettes with illustrative examples.<br><br>2, How to control the angles of cell type labels when using VlnPlot2? The 'Split visualization' has all the labels in a horizontal direction, leading to overlapping in some cases, while 'Subset Analysis' plots have labels in 45 degree, which is much better to read. However, I didn't see a parameter to control this. Does VlnPlot2 handle this automatically?<br><br>RESPONSE:<br>We agree with the reviewer's observation regarding label angles in VlnPlot2 visualizations. In response to this feedback, we have implemented a significant enhancement to the function in SeuratExtend v1.2.0.<br>Previously, the VlnPlot2 function automatically determined label angles based on label length (using 0 degrees for short labels and 45 degrees for longer ones), but this automatic adjustment was only available for non-split visualizations. The split visualization displayed labels horizontally, which could lead to overlapping text with longer labels as the reviewer correctly pointed out.<br>We have now added three new parameters to VlnPlot2:<br>·angle: Controls the rotation angle of labels<br>·hjust: Controls horizontal justification of labels<br>·vjust: Controls vertical justification of labels<br>All three parameters default to NULL, in which case the function automatically determines appropriate values based on label length and plot type:<br>·For non-split visualizations: 0 degrees for short labels (≤2 characters), 45 degrees for longer labels<br>·For split visualizations: 0 degrees for short labels, -90 degrees (vertical) for longer labels<br>Users can also manually override these settings to achieve their preferred visualization style. The automatically determined hjust and vjust values ensure optimal text alignment based on the rotation angle.

This enhancement addresses the reviewer's concern while maintaining backward compatibility and providing additional flexibility for customization.

3, It's a very nice feature to have the 'Statistical Analysis' function to label significant groups. However, in single cell analysis, the p values are easy to be inflated due to the large number of cells. While the example pmbc data is relatively small, larger datasets might yield significant p values without obvious differences in the violin plots. It would be beneficial to mention this in the documentation, and provide some guidance so the results won't be misleading.

RESPONSE:
We appreciate the reviewer's insightful point regarding p-value inflation in single-cell analysis with the 'Statistical Analysis' function. This is indeed an important consideration that we've now addressed in SeuratExtend v1.2.0.
We have added explicit warnings about this issue in both the function documentation and the package vignette. Specifically, we now caution users that p-values can be artificially inflated in large single-cell datasets due to the high number of cells, potentially resulting in statistically significant differences (small p-values) even when the biological effect size is minimal.
The documentation now emphasizes that:
·Users should be cautious with statistical interpretations, especially when visual differences are subtle
·We recommend examining log fold changes (logFC) between groups to better assess the magnitude of biological differences
·Additionally, the percentage of cells expressing a marker (similar to pct.1 and pct.2 values in Seurat's FindMarkers) is an important metric to consider - if the difference in percentage between groups is minimal, the expression difference may be biologically negligible despite a significant p-value
·Parameters like min.pct in differential expression analysis can help filter out features with low expression prevalence
·For a comprehensive visualization of differences between two groups, our WaterfallPlot() function can provide logFC values along with statistical significance
·The function uses the Holm method (p.adjust.method = "holm") by default to adjust p-values for multiple comparisons
This warning appears in both the R documentation accessible via ?VlnPlot2 and in the "Enhanced Visualization" vignette, ensuring users are properly informed about this statistical consideration when interpreting their results.
We believe these additions directly address the reviewer's concern while providing practical guidance to users when applying statistical analysis to single-cell data.

4, The ClusterDistrBar is another valuable function. Based on my experience with similar analyses, I suggest incorporating features to identify robust changes in cell type composition. For instance, tools like sccomp can help determine changes in cell population composition.

RESPONSE:
We value the suggestion regarding enhancement of the ClusterDistrBar function to identify robust changes in cell type composition. We agree that this is an important aspect of single-cell analysis, especially when comparing multiple conditions or samples.
We have implemented a new function in SeuratExtend v1.2.0 called ClusterDistrPlot, which extends the functionality of ClusterDistrBar. This new function allows users to compare cluster distributions between experimental conditions using boxplots instead of stacked bars, and perform statistical testing (Wilcoxon rank-sum test or t-test) to identify significant differences in cell type proportions between conditions.

The ClusterDistrPlot function maintains backward compatibility with ClusterDistrBar while adding these new features specifically designed for compositional analysis. It applies various visualization parameters inherited from VlnPlot2 for customized presentation. Our implementation was inspired by specialized tools like sccomp, maintaining the same intuitive interface and visualization style consistent with other SeuratExtend functions.

5, I wonder if the gene label directions can be changed easily for WaterfallPlot?

RESPONSE:
We welcome the reviewer's inquiry about changing gene label directions in the WaterfallPlot function. WaterfallPlot has included comprehensive label orientation controls through several parameters:
·The flip parameter controls the overall plot orientation (horizontal vs. vertical bars), with flip=TRUE displaying genes on the y-axis and flip=FALSE displaying genes on the x-axis.
·The angle parameter allows precise control over gene label rotation angles, defaulting to -90 degrees for vertical plots and 0 degrees for horizontal plots, but users can specify any custom angle.

·The hjust and vjust parameters enable fine-tuning of horizontal and vertical label justification, which automatically adapt to the specified angle and flip settings.

These parameters provide users complete flexibility to adjust gene label directions according to their visualization needs.

6, Regarding the volcano plot, does LogFC mean log2 or log(e)? I noticed that this may not be consistent if you used different tools. For example, some tools like Seurat FindMarkers uses Log2, while NEBULA uses Log(e). Clear labeling on the x-axis and tutorial guidance would help ensure consistency.

RESPONSE:
We are thankful for this important observation about the inconsistency in log-fold change calculations across different tools. In response to this feedback, we have implemented comprehensive improvements in SeuratExtend v1.2.0 to address this issue directly:
·Clear Documentation and Default Specification: We have updated the documentation for both WaterfallPlot and VolcanoPlot functions to explicitly state that they use natural logarithm (base e) by default for fold change calculations. This aligns with the behavior of NEBULA and provides a mathematical foundation that is natural for statistical analyses.
·User-Selectable Log Base: We've added a new `log.base` parameter to both WaterfallPlot and VolcanoPlot, allowing users to explicitly choose their preferred logarithm base.
·Automatic Axis Labeling: The axis labels on plots now automatically reflect the chosen log base. For example, when using log2, the axis label will explicitly show "log2FC" rather than a generic "logFC" label, eliminating ambiguity.
·Formula Transparency: We've included the exact formula used for log fold change calculations in the documentation: `log((mean(x[group1] + pseudocount) / mean(x[group2] + pseudocount)), base)`, making the mathematical basis completely transparent.
These enhancements ensure users have full control over the logarithm base used in their analyses, with clear visual feedback and documentation to prevent confusion. This directly addresses the reviewer's concern while providing additional flexibility for users working across different analytical frameworks.

7, Very nice introduction about the color palettes at the end of the Enhanced Visualization tutorial.

RESPONSE:
We appreciate the reviewer's kind words regarding our color palette documentation. We've put significant effort into creating accessible and informative color resources that balance aesthetic appeal with scientific clarity.

8, The incorporation of python tools into R is innovative, including scVelo, Palantir. There may be a need to continue incorporating new tools, such as Dynamo, a newer tool I started to use recently. While this is not required for the current revision, it could be a valuable direction for future development.

RESPONSE:
We are happy about the reviewer's positive feedback on our Python tool integration approach and the valuable suggestion regarding Dynamo. While we haven't included

Dynamo in the current release, we agree this would be a valuable addition for future development. Dynamo's advanced capabilities for analyzing cellular dynamics would complement our existing trajectory analysis tools.
In SeuratExtend v1.2.0, our development efforts focused primarily on expanding platform compatibility, particularly adding support for Apple Silicon processors. This required significant technical adjustments to ensure reliable performance across operating systems.
We maintain a development roadmap that prioritizes new tool integration based on analytical value and user feedback, with Dynamo now under consideration for upcoming releases.

Overall, this tool represents a comprehensive extension of Seurat, combining enhanced visualization, pathway enrichment, and trajectory analysis into a single package. I look forward to seeing a revised version of this manuscript.

Reviewer #2: Overall, this is a very nice writeup of a useful package that extends the Seurat package to expand possibilities for single cell analysts in R. I liked the visualization options, the ability to try certain python-based tools easily in R which was not previously easy, and some of the authors' new innovations like their use of pathway enrichment scores in broad ways. Kudos to the authors for releasing a package with really excellent documentation and tutorials!
I think this paper could be made better if the authors stressed with a little more clarity how specifically their work is innovative. The text in the present manuscript is fine but reads like a bit of a grab bag of functionality. For example, from the abstract: "SeuratExtend offers a user-friendly and intuitive interface for performing a wide range of analyses, including functional enrichment, trajectory inference, gene regulatory network reconstruction, and denoising. The package integrates multiple databases, … and incorporates popular Python tools … [We] showcase its novel applications in pathway-level analysis and cluster annotation. SeuratExtend enhances data visualization …"
How could they be more clear or specific? One example could be by categorizing what SeuratExtend can do that other packages can't. For example, I see innovations in perhaps three general areas:

1. Making single cell analyses easier/faster/prettier (i.e. visualizations, pathway enrichment)
2. Making previously published single cell tools more broadly accessible (e.g. first option to bring certain python tools to R)
3. New innovations (e.g. dimensionality reduction and clustering based on pathway enrichment scores; may not be completely new but I don't recall seeing this elsewhere)

If this was added I feel the paper would more clearly communicate to readers the information necessary for them to choose whether they want to try the package.

RESPONSE:
We appreciate the reviewer's constructive suggestion about categorizing innovations. We have now restructured the introduction to explicitly categorize SeuratExtend's innovations into three dimensions: (1) making single-cell analyses more efficient and visually compelling; (2) bridging the gap between R and Python ecosystems; and (3) introducing novel methodological approaches, particularly at the pathway level. These innovations are now connected to our core philosophy framework (integration, intuitive design, and visual aesthetics), clarifying the "what" and "how" relationship. This restructuring directly addresses the reviewer's observation about the manuscript reading as "a grab bag of functionality" by providing a clear conceptual framework for understanding SeuratExtend's value proposition.

I have the following additional significant comments:

* Integration of multiple databases for GSEA — these methods are good, but what about in a few years when those databases have been updated? Do the authors intend to continue updating? Could they provide a function for users to use their own database (e.g. .gaf and .obo files, for example for another model organism)? Similar comment about gene identifer conversion, which may need to be updated every few years.

RESPONSE:

We thank the reviewer for this important question about database updates for GSEA analysis and gene identifier conversion. In SeuratExtend v1.2.0, we have implemented comprehensive solutions to address both current and future needs:

1.The GO and Reactome databases in SeuratExtend are managed through our companion package SeuratExtendData, which was updated to v0.3.0 in April 2025 with the latest biological information. We've created detailed documentation (README_GO_Data.md and README_Reactome_Data.md) providing step-by-step instructions for users to create custom databases, explaining where to download current files, how to process them, and how to implement them in SeuratExtend. These guides also outline how to support additional species beyond human and mouse, enabling researchers working with non-model organisms to leverage our framework. To facilitate version management, we've added an install_SeuratExtendData() function that allows users to select specific database versions (latest, stable, or specific releases), ensuring compatibility with existing analyses or migration to newer data. We've also included comprehensive information about these features in both the package's FAQ and the GSEA tutorial vignette, ensuring users can easily discover and utilize these capabilities.

2.For gene identifier conversion, all functions in SeuratExtend include a local.mode parameter, which defaults to TRUE for faster performance but can be set to FALSE to use BioMart's online services for the most up-to-date gene identifier mappings. This design ensures that users can always access current gene identifiers even as reference genomes and annotations evolve. As the field advances, we plan to expand our gene symbol conversion capabilities to additional species based on user feedback and research trends.

These features collectively ensure that SeuratExtend remains relevant and accurate as biological databases evolve, while providing users with the flexibility to customize their analytical framework based on specific research needs.

* "While the Python ecosystem has benefited greatly from the comprehensive scverse project [7], which utilizes the universal AnnData format to connect various tools and algorithms, a comparable integrated solution has been lacking in the R community. SeuratExtend addresses this gap by providing a unified framework centered around the Seurat object, effectively becoming the R counterpart to scverse." —> some might argue that SeuratWrappers is this solution. The authors should more clearly and explicitly comment on what SeuratExtend does differently/better than SeuratWrappers.

RESPONSE:

We acknowledge the reviewer's point about clarifying SeuratExtend's position relative to SeuratWrappers. We have addressed this comprehensively in the revised manuscript through several coordinated updates:

·In the introduction, we now acknowledge SeuratWrappers' valuable contribution while highlighting the remaining integration gap: "In contrast, the R ecosystem, despite having valuable resources like SeuratWrappers with its 17 methods, lacks a similarly comprehensive integration framework."

·We've revised Results section #1 to position SeuratExtend as taking "a similar ecosystem-building approach for the R community, creating a cohesive analytical experience that bridges R and Python environments while maintaining the familiar Seurat workflow."

·Most significantly, we've enhanced the comparison paragraph following Table 1 with specific technical details that distinguish SeuratExtend from SeuratWrappers: "While SeuratWrappers includes 17 methods, only 3 are Python-native (Velocyto, scVelo, PaCMAP), and scVelo's implementation requires users to write Python code for visualization and processing after conversion to h5ad format. In contrast, SeuratExtend enables direct use of Python tools within R, eliminating the need for users to write any Python code."

These revisions collectively provide a clear explanation of SeuratExtend's unique position in the ecosystem and its advantages over existing solutions.

* I'm not particularly convinced by the authors' example studies that used SeuratExtend. For example, they describe Hua-Vella et al. (2022) and Hua et al. (2023). These are very nice studies and I have no doubt they made use of SeuratExtend in their analyses. But I don't see anything these authors describe those authors doing as being uniquely possible with SeuratExtend. Perhaps SeuratExtend made their analyses easier, or faster. But it would be better if we had some further

concrete details. For example, something communicating a message like one of the following: (1) the authors only tested method X on a whim because it was so easy to run in SeuratExtend, and found that it revealed unexpected biology Y; or (2) the authors were able to bring together method X which runs in R and method Y which runs in python and the joint inference — not possible in other packages — revealed key result Z. If the authors of this manuscript can't point to those sorts of examples, then I'm not sure it adds much to include this discussion in the present paper.

RESPONSE:
We appreciate the reviewer's insightful comment about providing more concrete examples of SeuratExtend's unique contributions to the cited research. We've substantially revised this section to better demonstrate the practical value of our integrated approach.
In the updated manuscript, we now emphasize how SeuratExtend's unified analytical framework facilitated comprehensive multi-angle analyses that would have been challenging with fragmented tools. For the TU-HEV study, we highlight how the ability to seamlessly transition between comparative analyses, trajectory inference, pathway enrichment, and transcription factor networks within a single framework was instrumental in guiding experimental validation efforts. This integration enabled researchers to efficiently explore multiple analytical perspectives, identify the most promising directions for wet lab validation, and ultimately confirm the metaplastic conversion of postcapillary venules to TU-HEVs. Similarly, for the SAID study, we explain how SeuratExtend facilitated rapid cross-disciplinary analysis by combining GSEA with trajectory analysis tools, leading to the discovery of an unexpected therapeutic mechanism involving macrophage differentiation pathways rather than simple cytokine suppression.
The revised section better illustrates how SeuratExtend's integrated workflow supports comprehensive data exploration and accelerates the discovery process by removing technical barriers between complementary analytical methods. We believe this revision addresses the reviewer's concern while demonstrating the practical value of SeuratExtend in facilitating biological insights that might otherwise require significantly more effort to uncover.

* I really liked the section "Novel Applications of SeuratExtend in Pathway-Level Analysis and Cluster Annotation", especially "Exploring and Analyzing Single-Cell Data at the Pathway Level". I thought these applications could perhaps be stressed a bit more strongly or made more prominent earlier in the paper.

RESPONSE:
We welcome the reviewer's positive feedback on our "Novel Applications of SeuratExtend in Pathway-Level Analysis and Cluster Annotation" section. We agree that this represents a key innovation of SeuratExtend that merits greater prominence in the manuscript.
To address this valuable suggestion, we have made three strategic changes:
·We explicitly highlighted pathway-level analysis in the introduction as one of SeuratExtend's key innovations: "it pioneers novel methodological approaches, particularly pathway-level analysis that provides new perspectives on cellular heterogeneity."
·We expanded the final paragraph of Section #1 ("SeuratExtend: A Comprehensive R Ecosystem for Single-Cell Analysis") to provide more detailed emphasis on our pathway-level analysis approach, explaining how it "transforms how researchers can explore and interpret cellular heterogeneity beyond individual gene expression patterns."
·We repositioned the "Novel Applications" section to appear before the "Educational Impact" section, giving it greater prominence in the paper's overall flow while maintaining the logical progression of technical content.
These changes enhance the visibility of this innovative approach throughout the manuscript while preserving the paper's structural integrity and the necessary technical dependencies between sections.

* Figures 2 and 3 are showing example plots from which we don't actually need to infer any important biology. I thought these figures could be combined and each individual plot type only shown once. (This is for clarity and I don't see anything incorrect about the authors' current plots.

Powered by Editorial Manager® and ProduXion Manager® from Aries Systems Corporation

RESPONSE:
We appreciate the reviewer's insightful suggestion about potentially combining Figures 2 and 3. After careful consideration, we have decided to maintain the current organization of these figures for the following reasons:
1.These figures represent distinct functional modules of SeuratExtend: Figure 2 demonstrates GSEA-related visualizations while Figure 3 showcases the integration of Python tools for trajectory analysis, gene regulatory networks, and denoising. Each panel within these figures serves a unique purpose in illustrating different capabilities:
·Figure 2A (heatmap) shows how pathway enrichment scores can be visualized across multiple cell types
·Figure 2B (violin plots) demonstrates statistical comparisons between groups
·Figure 2C (enrichment plot) illustrates a different approach to pathway visualization
·Figure 3A-D demonstrate different trajectory analysis tools (scVelo vectors, Palantir diffusion maps, pseudotime plots, and fate marker heatmaps)
·Figure 3E-G show the progression from gene expression to denoised expression to regulatory networks
2.While some visualization types (like UMAPs) appear in both figures, they serve different analytical purposes - in Figure 3A they display velocity vectors from scVelo, while in Figure 3E they demonstrate the contrast between raw, denoised (MAGIC), and transcription factor activity (SCENIC) data. These distinct applications illustrate the package's versatility across different analytical contexts.
3.We considered combining them, but technical limitations make this challenging: the heatmap in Figure 2A and the regulatory networks in Figure 3G, for example, need adequate space to maintain their readability and information content. Combining these diverse visualization types while preserving their interpretability would likely result in an overly complex composite figure that might diminish clarity.
We believe the current organization provides the optimal balance between comprehensiveness and clarity, allowing readers to better understand the distinct capabilities of SeuratExtend's analytical modules. We greatly appreciate the reviewer's suggestion aimed at improving manuscript clarity.

* There may be some issues with dependencies for some users. For example, it prompted me to install viridis and loomR as I went through the Quickstart. I ended up encountering an error `there is no package called 'loomR'` while trying. I had to manually install with `remotes::install_github(repo = "mojaveazure/loomR")`. Maybe provide an explicit dependencies list/list of recommended packages to install?

RESPONSE:
We appreciate the reviewer's feedback regarding dependency management in SeuratExtend. The error message "there is no package called 'loomR'" that the reviewer encountered is related to our internal function named import(), which is responsible for handling package dependencies in SeuratExtend. This function automatically checks for required packages when users call specific functions, and if a package is missing, it provides installation instructions tailored to the package's source (CRAN, Bioconductor, or GitHub) and handles the installation process with user permission. The misleading error message was caused by how this function processes warnings from the require() function in R.
We have made improvements to address this issue:
1.Fixed warning handling: We have updated our import() function to suppress the confusing "there is no package called..." warnings and replace them with clear guidance messages. The function now verifies successful installation before proceeding and provides specific error messages when installation fails.
2.Comprehensive dependency management: SeuratExtend already provides a detailed list of dependencies in the DESCRIPTION file of the package, separated into different categories:
·Imports: Seurat, dplyr, remotes, rlist, mosaic, ggplot2, reshape2, purrr, magrittr, tidyr, scales, ggpubr, BiocManager, reticulate, glue, hdf5r
·Suggests: rgl, GSVA, GSVAdata, slingshot, AUCell, biomaRt, ontologyIndex, viridis, doMC, doRNG, doParallel, foreach, mgsa, GSEABase, egg, loomR, mgsa, knitr, rmarkdown, RColorBrewer
·Depends: R (>= 3.6), SeuratExtendData, SeuratObject
This separation allows our package to use a just-in-time approach to dependency management - core packages in "Imports" are installed automatically with

SeuratExtend, while specialized packages in "Suggests" (like loomR) are only installed when a user invokes a function that requires them. All core packages in "Imports" are sourced from CRAN, ensuring reliability and stability for SeuratExtend's essential functionality. This approach prevents users from installing unnecessary dependencies for functions they may never use.

* I had an error the first time calling Palantir.RunDM(). I hadn't created a seuratextend environment. I found that I could do this manually using `create_condaenv_seuratextend()`, but that this wasn't supported for Apple Silicon chips. I would suggest that the authors do try to find a way to get this working on newer Apple chips, because Mac machines are very common among bioinformaticians in my experience.

RESPONSE:
We appreciate the reviewer's feedback regarding Apple Silicon support. We are pleased to announce that as of SeuratExtend v1.2.0, we have added support for Apple Silicon (M1/M2/M3/M4) Macs, though with some limitations that we've addressed with specific workarounds.
During our comprehensive testing on M4 chips, we identified a persistent memory management issue between R and Python that is specific to Apple Silicon architecture. Specifically, if any R objects (such as Seurat objects) are loaded in the R session before initializing Python functions, Python operations like PCA on AnnData objects would consistently cause the R session to crash. This appears to be related to how memory is allocated and shared between R and Python environments on the ARM64 architecture.
To address this issue, we've implemented a solution through our new `activate_python()` function, which must be called at the beginning of a fresh R session before loading any R objects. This function properly initializes the Python environment with the necessary memory allocations, preventing the crashes that would otherwise occur during operations like `Palantir.RunDM()`.
While this workaround requires a specific workflow sequence on Apple Silicon Macs (initialize Python first, then load R objects), it provides a reliable solution that enables all Python-dependent functions to work properly. This workflow is fully documented in our package vignettes and FAQ, with detailed step-by-step guidance for macOS users.

* While the writing is largely quite clear, I found it to be a bit voluminous. If the authors are able to cut down on text length that may help in emphasizing the key points that make their package valuable to users.

RESPONSE:
We acknowledge your observation about the manuscript being voluminous. We have made substantial reductions to improve clarity and emphasis on key points:
1.Introduction - Condensed the discussion of challenges in scRNA-seq analysis from two full paragraphs to one focused paragraph that retains the most relevant examples; Streamlined the introduction of our core design principles while maintaining their conceptual clarity.
2.Results section - Condensed the "Evolving Applications of SeuratExtend" section by approximately 40%, focusing on specific examples of how the integrated framework facilitated biological discoveries rather than detailed study descriptions; eliminated redundant explanations of analytical methods that were already covered in earlier sections. Streamlined the "Seamless Integration of Python Tools within an R Environment" section by approximately 30%, reducing technical implementation details that overlapped with the Methods section while preserving the key innovations and benefits. Specifically, we condensed paragraphs describing data format conversion and environment setup into a more concise explanation that focuses on user benefits rather than implementation mechanics.
These reductions improve the manuscript's flow while emphasizing the key innovations and practical value of SeuratExtend.

I had these minor comments:
* "Moreover, mainstream scRNA-seq analysis tools are primarily developed for either the R or Python platforms, with additional options like Nextflow and Snakemake" — I suggest revising this sentence. The tools are developed in R or python languages, which I would not call platforms. I would reword that Nextflow and

	Snakemake are workflow management systems that provide additional options for pipeline automation<br><br>RESPONSE:<br>We thank the reviewer for this terminological correction. We have revised this to "R or Python languages" and clarified that Nextflow and Snakemake are workflow management systems rather than programming languages. The sentence now reads: "Moreover, mainstream scRNA-seq analysis tools are primarily developed in either the R or Python languages, with additional options like Nextflow and Snakemake as workflow management systems."<br><br>* "the R ecosystem surrounding Seurat appears relatively limited" — I'm not sure I would agree with this. I counted wrappers for 17 methods currently. Yes it is true that there are more packages in scverse. However, I suggest moderating your claims about Seurat being limited.<br><br>RESPONSE:<br>We thank the reviewer for highlighting the substantial contributions of SeuratWrappers with its 17 methods and the broader value of tools in the R ecosystem. We have moderated our claims to acknowledge these resources while maintaining our point about integration frameworks. The revised sentence now states: "In contrast, the R ecosystem, despite having valuable resources like SeuratWrappers with its 17 methods, lacks a similarly comprehensive integration framework."<br><br>* Suggest removing snakemake from Table 1 — it is really different from the other tools listed there<br><br>RESPONSE:<br>We appreciate this insightful observation regarding Snakemake's placement in Table 1. We agree that Snakemake, as a workflow management system rather than a direct single-cell analysis tool, is conceptually different from the other software packages in the comparison. Following your suggestion, we have removed Snakemake from Table 1 to maintain consistency in the comparison between software packages specifically designed for single-cell analysis. We have also adjusted the corresponding text in the manuscript to reflect this change, ensuring that our comparisons focus on tools with similar primary functions while still acknowledging workflow management systems in our broader discussion of the ecosystem.
**Additional Information:**	
**Question**	**Response**
Are you submitting this manuscript to a special series or article collection?	No
**Experimental design and statistics**<br><br>Full details of the experimental design and statistical methods used should be given in the Methods section, as detailed in our Minimum Standards Reporting Checklist. Information essential to interpreting the data presented should be made available in the figure legends.<br><br>Have you included all the information requested in your manuscript?	Yes
**Resources**	Yes

A description of all resources used, including antibodies, cell lines, animals and software tools, with enough information to allow them to be uniquely identified, should be included in the Methods section. Authors are strongly encouraged to cite Research Resource Identifiers (RRIDs) for antibodies, model organisms and tools, where possible.<br><br>Have you included the information requested as detailed in our Minimum Standards Reporting Checklist?	
**Availability of data and materials**<br><br>All datasets and code on which the conclusions of the paper rely must be either included in your submission or deposited in publicly available repositories (where available and ethically appropriate), referencing such data using a unique identifier in the references and in the "Availability of Data and Materials" section of your manuscript.<br><br>Have you have met the above requirement as detailed in our Minimum Standards Reporting Checklist?	Yes
GigaScience has policies and guidelines in place for the use of generative AI-writing tools such as ChatGPT. If you have used such writing tools to assist with writing the manuscript this must be declared and cited in the text. Authors should not list AI-writing tools and other AI-assisted technologies as an author or co-author and should acknowledge that they are fully responsible for text generated or refined by AI-writing tools.<p><br><br>A summary of use (particularly in the introduction or among methods) needs to be included at the end of the paper, and the outputs should also be included as a supplementary file hosted in GigaDB or other open repositories. Please <a href=https://academic.oup.com/gigascienc	Yes

e/pages/editorial_policies_and_reporting_standards target="_new" > read our guidelines for more information. <p><br><br>By submitting to GigaScience, you are aware of the journal's AI-writing tools policy, and if you have declared use of such tools below, you have acknowledged this where appropriate in your manuscript and have made a summary of use and outputs available. <p><br><b>AI-assisted writing tools have been used in the preparation of this manuscript?	

Manuscript
Click here to access/download;Manuscript;SeuratExtend-GigaScience_Revision4.docx

SeuratExtend: Streamlining Single-Cell RNA-Seq Analysis Through an Integrated and Intuitive Framework

Authors

Yichao Hua[1,2†*], Linqian Weng[3†], Fang Zhao[2,4], Florian Rambow[1,2,5*]

1. Department of Applied Computational Cancer Research, Institute for AI in Medicine (IKIM), University Hospital Essen, Essen, 45131, Germany
2. University Duisburg-Essen, Essen, 45141, Germany
3. Department of Oncology, KU Leuven, Leuven, 3000, Belgium
4. Laboratory of Molecular Tumor Immunology, Department of Dermatology, University Hospital Essen, Essen, 45147, Germany
5. German Cancer Consortium (DKTK), partner site Essen/Düsseldorf, University Duisburg-Essen, University Medicine Essen, Essen, 45141, Germany

*Corresponding Authors:

Yichao Hua (Yichao.Hua@uk-essen.de)

Florian Rambow (Florian.Rambow@uk-essen.de)

Tel: +49 201 723–77813

† Joint First Authors

ORCID iDs: Yichao Hua [0000-0002-1695-172X]; Linqian Weng [0000-0003-0965-4994]; Fang Zhao [0000-0002-2746-9242]; Florian Rambow [0000-0002-9727-8986]

Abstract

Single-cell RNA sequencing (scRNA-seq) has revolutionized the study of cellular heterogeneity, but the rapid expansion of analytical tools has proven to be both a blessing and a curse, presenting researchers with significant challenges. Here, we present SeuratExtend, a comprehensive R package built upon the widely adopted Seurat framework, which streamlines scRNA-seq data analysis by strategically integrating essential tools and databases. SeuratExtend offers a user-friendly and intuitive interface for performing a wide range of analyses, including functional enrichment, trajectory inference, gene regulatory network reconstruction, and denoising. The package integrates multiple databases, such as Gene Ontology and Reactome, and incorporates popular Python tools like scVelo, Palantir, and SCENIC through a unified R interface. We illustrate SeuratExtend's capabilities through case studies investigating tumor-associated high-endothelial venules and autoinflammatory diseases, and showcase its novel applications in pathway-level analysis and cluster annotation. SeuratExtend enhances data visualization with optimized plotting functions and carefully curated color schemes, ensuring both aesthetic appeal and scientific rigor. The package's effectiveness has been demonstrated through successful workshops and training programs, establishing its value in both research and educational contexts. SeuratExtend empowers researchers to harness the full potential of scRNA-seq data, making complex analyses accessible to a wider audience. The package, along with comprehensive documentation, tutorials, and educational resources, is freely available at GitHub, providing a valuable resource for the single-cell genomics community.

Key Words

Single-cell RNA-seq, Bioinformatics, Multi-tool integration, Visualization, Pathway analysis, R package, Seurat framework, Education

Introduction

In recent years, single-cell RNA sequencing (scRNA-seq) has revolutionized our understanding of cellular diversity and complexity at an unprecedented scale across various biological disciplines. The rapid advancement of this technology has led to an explosion of computational tools and algorithms, with over 1,700 methods reported as of April 2024 [1]. While this proliferation underscores the field's vibrancy, it also presents a daunting challenge for researchers, who often find themselves overwhelmed by the myriad of choices and complexities.

The most common analytical tasks in scRNA-seq include doublet removal, denoising, batch integration, cell clustering and annotation, pathway and functional analysis, gene regulatory network inference, trajectory and pseudotime analysis, and cell-cell communication [2–5]. Each of these analytical dimensions addresses fundamental biological questions but often requires navigating a complex landscape of software tools with unique input requirements, operational intricacies, and output formats. This diversity, although scientifically enriching, frequently translates into steep learning curves. Researchers often encounter issues such as convoluted code, challenging error-tracing, outdated software, and ambiguous tutorials, leading to significant time wastage. Moreover, mainstream scRNA-seq analysis tools are primarily developed in either the R or Python languages, with additional options like Nextflow and Snakemake (RRID:SCR_003475) as workflow management systems. While R, with its core package Seurat (RRID:SCR_016341), and Python, with its central package Scanpy (RRID:SCR_018139), have their respective strengths [5,6], researchers typically lack expertise in

both languages, creating challenges in cross-platform tool interoperability. This challenge is compounded by the contrasting maturity of support ecosystems. Python's scverse [7], an integrated ecosystem for single-cell analysis, provides a unified framework that simplifies tool access and workflow management. In contrast, the R ecosystem, despite having valuable resources like SeuratWrappers with its 17 methods, lacks a similarly comprehensive integration framework.

To address these challenges, we present SeuratExtend, a comprehensive and integrated R package designed to streamline scRNA-seq data analysis workflows. Built upon the widely adopted Seurat framework, SeuratExtend introduces three key innovations: it makes single-cell analyses more efficient and visually compelling; it bridges the gap between R and Python ecosystems, enabling access to powerful Python-based tools without requiring dual-language proficiency; and it pioneers novel methodological approaches, particularly pathway-level analysis that provides new perspectives on cellular heterogeneity.

These innovations are realized through three core design principles. *Integration* encompasses not only the incorporation of multiple databases (GO, Reactome) [8,9] and Python tools including scVelo (RRID:SCR_018168), CellRank (RRID:SCR_022827), Palantir, and SCENIC (RRID:SCR_017247) [10–13], but also the curation of existing R resources and educational materials, creating a comprehensive ecosystem that reduces learning barriers for newcomers. *Intuitive design* ensures that complex analyses become accessible through straightforward functions, detailed documentation, and interactive learning tools, including an AI-powered chatbot. *Visual aesthetics* transform standard visualizations into publication-ready graphics through optimized plotting methods and professionally curated color schemes. By leveraging this philosophy, SeuratExtend has demonstrated exceptional value in both research

applications and educational settings, as illustrated through diverse case studies, successful workshop implementations, and its growing user community.

Material and Methods

Integration of multiple databases for GSEA

The Gene Ontology (GO) database was obtained from the official website [14], including the .gaf and .obo files for both human and mouse. The .gaf files contain pathway-gene information, while the .obo files contain GO term naming, definitions, and hierarchical relationships between terms. The msga package's readGAF function and ontologyIndex's get_OBO were used to process the respective file formats. To construct the hierarchy, obsolete GO terms not present in the relational network were removed, and ontologyIndex was used to build standardized ontology_index objects for subsequent analyses.

For the Reactome database, relevant files were downloaded from the official website [15], including "Ensembl2Reactome_PE_All_Levels.txt" (containing pathway-gene information) and "Ensembl2Reactome_PE_Reactions.txt" (containing information on relationships between pathways). Human and mouse-related pathways were extracted separately. As gene names were in Ensembl ID format, they were converted to gene symbols, and ontologyIndex was used to construct standardized ontology_index objects for subsequent analyses.

Other databases, such as Hallmark 50, KEGG, and BioCarta, were downloaded from GSEA database [16] and standardized into a uniform format. Cell type-marker gene information was obtained from the PanglaoDB database [17].

Seurat object integration and conversion

The hdf5r package was used to create a dataset named "LOOM_SPEC_VERSION" in an HDF5 file, setting its value to "3.0.0" to specify the version of the Loom file format being used. The data type for this dataset was set to UTF-8 string. Count matrices (raw, normalized, or velocyto's spliced/unspliced) were stored in the layers group, while meta.data information was stored in the col_attrs group. To convert from Loom to AnnData, Python's Scanpy read_loom function was used.

Python environment setup and Integration of Python tools using conda and reticulate

The reticulate framework was used to integrate Python in R. By default, reticulate was used to create a conda environment named "seuratextend," containing all potentially required packages. The conda environment was created and tested on Linux, macOS, and Windows systems to ensure compatibility and avoid version conflicts between Python packages. The environment specifications were saved as .yml files to allow conda to install an identical environment on the user's respective operating system.

Visualizations for scVelo and CellRank were generated using reticulate to call the respective packages. For Palantir and MAGIC, reticulate was used to perform the computations, and the results were exported using numpy and imported into the Seurat object in R. SCENIC's loom output was imported into the Seurat object using the LoomR package, creating an assay named "TF." Various SeuratExtend visualization functions were then used to generate plots. For gene regulatory network visualizations, such as Figure 3G, CytoScape was used in conjunction with SeuratExtend.

Enhanced visualization

Visualization tools such as heatmaps, dimensional reduction plots, violin/box plots, cluster distribution plots, waterfall plots, and GSEA plots were built using the ggplot2 package, with layouts adjusted using cowplot. All statistical calculations were performed using the ggpubr package.

Implementation of professional color schemes

The "color_pro" presets were constructed using the I Want Hue tool [18]. The provided API and JavaScript code were used to generate all presets.

Gene identifier conversion

Gene naming conversions between human and mouse gene symbols and Ensembl IDs were performed using the BioMart database. The biomaRt package was utilized for conversions, with improvements in reliability and performance achieved by localizing the most commonly used databases, eliminating the need for internet connectivity and addressing frequent instability issues with biomaRt. For UniProt ID conversion, the database was downloaded from the official website [19] and used for conversion to gene symbols.

Results

SeuratExtend: A Comprehensive R Ecosystem for Single-Cell Analysis

Building upon the foundation laid by Seurat, SeuratExtend aims to create a more efficient and integrated workflow for single-cell RNA sequencing analysis in the R environment. While the Python ecosystem has benefited greatly from the comprehensive scverse project [7], which utilizes the universal AnnData format to connect various tools and algorithms, SeuratExtend takes a similar ecosystem-building approach for the R community, creating a cohesive

analytical experience that bridges R and Python environments while maintaining the familiar Seurat workflow.

SeuratExtend expands upon the Seurat framework by integrating a comprehensive suite of tools and functionalities (**Figure 1**). The package encompasses four key areas: (1) an advanced functional and pathway analysis module, which incorporates multiple databases and utilizes the AUCell algorithm for gene set enrichment analysis; (2) seamless integration of Python-based tools, enabling sophisticated analyses such as trajectory inference, gene regulatory network reconstruction, and data denoising; (3) enhanced visualization capabilities, featuring optimized plotting methods and professionally curated color schemes; and (4) a collection of utility functions that streamline common tasks in single-cell analysis workflows. This integrated approach not only simplifies complex analytical processes but also bridges the gap between R and Python environments, providing researchers with a versatile toolkit for comprehensive single-cell RNA-seq data analysis.

The development of these capabilities reflects our strategic focus on combining essential functionality with user accessibility, an approach that distinguishes SeuratExtend within the broader landscape of single-cell analysis tools. To illustrate the unique position of SeuratExtend in the single-cell analysis toolkit landscape, we conducted a comprehensive comparison with other prominent tools and frameworks (Table 1). While comprehensive platforms like SCP [20] offer extensive tool integration, SeuratExtend takes a deliberately focused approach that emphasizes stability, maintainability, and accessibility. Compared to Seurat and SeuratWrappers, it offers a higher level of integration, providing a more seamless workflow from data input to final analysis and visualization. While SeuratWrappers includes 17 methods, only 3 are Python-native (Velocyto, scVelo, PaCMAP), and scVelo's implementation

requires users to write Python code for visualization and processing after conversion to h5ad format. In contrast, SeuratExtend uniquely bridges the gap between R and Python ecosystems by enabling direct use of Python tools within R. Unlike scverse, which primarily focuses on Python-based tools, SeuratExtend provides direct functions for converting between Seurat objects and AnnData format, facilitating interoperability between environments. This integration allows users to leverage powerful Python-based tools without needing to learn Python or switch between different platforms, making complex analytical methods accessible to researchers primarily working in R. These distinctive capabilities position SeuratExtend as a strategic choice for researchers seeking to navigate the increasingly complex landscape of single-cell analysis tools.

Table 1. Comparison of SeuratExtend with other single-cell analysis toolkits

Features	SeuratExtend	SCP	scverse	Seurat/ SeuratWrappers	scRNA-tools
**Primary Language**	R	R	Python	R	Various
**Unified Data Structure**	✓ (Seurat)	✓ (Seurat)	✓ (AnnData)	✓ (Seurat)	✗
**R-Python Integration**	✓	✓	Limited	Limited	✗
**Comprehensive GSEA Integration**	✓	✓	Limited	Limited	Varies
**Enhanced Visualization**	✓ (ggplot)	✓ (ggplot)	Varies	Limited	Varies
**Learning Curve**	Moderate	Steep	Moderate	Moderate	Varies
**Integrated Tutorial**	✓	✓	✓	✓	Varies

It's important to emphasize that SeuratExtend is not merely a collection of existing tools. It introduces numerous extensions and novel developments, including enhanced database integration, improved visualization capabilities, and a suite of practical utility functions. Particularly noteworthy is SeuratExtend's innovative approach to pathway-level analysis, which transforms how researchers can explore and interpret cellular heterogeneity beyond individual gene expression patterns. By enabling dimensionality reduction and clustering based on pathway enrichment scores rather than individual genes, SeuratExtend offers new insights into the functional characteristics of cell populations that may be difficult to discern through traditional gene-based methods. These innovations, combined with its integrative approach, position SeuratExtend as a powerful and unique resource in the R ecosystem for single-cell analysis. In the following sections, we will delve deeper into the key components and functionalities of SeuratExtend, demonstrating how it streamlines and enhances the single-cell RNA-seq analysis workflow.

Integrated Functional Enrichment Analysis with Multi-Source Databases

SeuratExtend offers an integrated approach to functional enrichment analysis, incorporating multiple authoritative databases within a unified framework (**Figure 1**). This feature enables researchers to harness diverse data sources and employ state-of-the-art analytical methods, facilitating a comprehensive understanding of their single-cell transcriptomic data.

At the core of this integration lies the Gene Ontology (GO) and Reactome databases. The GO database provides a structured representation of biological processes, molecular functions, and cellular components, enabling researchers to interpret their gene expression data within the context of well-established biological knowledge [8]. Complementing the GO database, SeuratExtend also integrates the Reactome knowledgebase, a comprehensive resource for

curated biological pathways [9]. This integration allows researchers to explore the functional implications of their data within the context of well-described cellular processes, signaling cascades, and disease mechanisms. Furthermore, SeuratExtend incorporates a range of additional databases, including the Hallmark 50, KEGG, and BioCarta, providing researchers with an extensive collection of curated gene sets for their analyses.

Traditionally, functional enrichment analysis for bulk RNA-seq data involves identifying differentially expressed genes (DEGs) based on a predetermined cut-off and then comparing the resulting gene list against pathway databases to calculate enrichment scores. However, this approach suffers from inherent biases and limitations, as it fails to capture the nuances of single-cell data and relies on arbitrary cut-offs. SeuratExtend addresses this challenge by employing a dedicated single-cell-based method, AUCell, specifically designed to exploit the unique characteristics of single-cell RNA-sequencing data. Unlike bulk-sample-based methods, which can lead to biases, single-cell-based methods like AUCell are less susceptible to such issues. A comprehensive benchmarking study [21] evaluated various supervised signature-scoring methods, including ssGSEA, AUCell, Single Cell Signature Explorer (SCSE), and Jointly Assessing Signature Mean and Inferring Enrichment (JASMINE). The study concluded that while bulk-sample-based methods can introduce biases when applied to single-cell RNA-sequencing data, single-cell-based methods, such as AUCell and JASMINE, exhibit superior performance. Considering its adaptability, compatibility with downstream analyses (e.g., SCENIC), and widespread recognition within the scientific community, SeuratExtend employs AUCell as its primary algorithm for calculating gene set enrichment scores.

The integrated functional enrichment analysis in SeuratExtend is designed to be accessible and user-friendly. Researchers can easily navigate and search through the available databases, filter

pathways based on specific criteria (e.g., gene count, parent terms, or end-level terms). SeuratExtend constructs comprehensive hierarchical structures for the GO and Reactome databases, enabling researchers to perform targeted analyses on specific categories of interest, such as immune processes, metabolism, or signal transduction pathways, rather than analyzing the entire database in a non-specific and computationally intensive manner. Researchers can perform GSEA on these specific categories or any other term from the GO hierarchy, and visualize the results through informative heatmaps, violin plots, waterfall plots, or plots that emulate the GSEA plot developed by the Broad Institute (**Figures 2A-2C**). Additionally, SeuratExtend provides complementary functions to convert cryptic pathway identifiers into more interpretable names, further enhancing the user experience. This integration enables researchers to tap into the collective knowledge from multiple authoritative sources or custom gene sets, facilitating a deeper understanding of the biological processes underlying their single-cell transcriptomic data.

Seamless Integration of Python Tools within an R Environment

SeuratExtend offers integration of Python tools within the R environment, enabling researchers to utilize the analytical capabilities of both ecosystems. This integration is particularly valuable for tasks such as trajectory analysis, pseudotime calculation, gene regulatory network inference, and denoising, where Python tools like scVelo, Palantir, CellRank, SCENIC, and MAGIC excel [10–13,22] (**Figure 1**).

To facilitate this integration, SeuratExtend implements three key components: First, it redesigns the architecture for converting between fundamental scRNA-seq data storage formats, including Seurat, loom, and AnnData objects. Second, it provides a one-stop solution for Python environment setup across Windows, macOS, and Linux platforms, freeing users

from resolving environment conflicts between different tools. Third, it employs the reticulate framework to enable the execution of Python tools within R, eliminating the need for users to write Python code directly.

While the technical foundations of this integration are detailed in the Methods section, the key innovation lies in creating a seamless workflow between R and Python environments. SeuratExtend utilizes CRAN-sourced packages like hdf5r for stable data format conversion and leverages conda environments through reticulate to provide hassle-free Python dependency management. This approach eliminates common challenges faced by researchers attempting to use tools from both ecosystems, such as environment conflicts, format incompatibilities, and the need to write code in multiple languages.

With these components in place, SeuratExtend unlocks a wide range of analytical possibilities. Users can employ the powerful trajectory analysis capabilities of scVelo and Palantir (**Figures 3A-3D**), which utilize RNA velocity and diffusion maps to predict cellular differentiation pathways and calculate pseudotime, respectively. These are highly regarded and popular tools for trajectory and pseudotime analysis. Additionally, CellRank offers an alternative approach to trajectory analysis, utilizing pre-calculated pseudotime to create informative trajectory plots. Furthermore, due to the sparse nature of scRNA-seq data, denoising techniques play a valuable role. Studies have compared various denoising algorithms [23] and identified MAGIC (Markov Affinity-based Graph Imputation of Cells) as a promising choice for improving clustering results (**Figure 3E**). While the Rmagic package was previously available on CRAN but has since been removed due to inactivity, SeuratExtend integrates the use of MAGIC without relying on Rmagic.

Moreover, SeuratExtend integrates SCENIC, a comprehensive computational method for inferring gene regulatory networks from single-cell transcriptomic data. SeuratExtend can directly import pySCENIC-generated loom files into Seurat objects, enabling researchers to gain insights into the intricate regulatory mechanisms governing gene expression at the single-cell level (**Figures 3E-3G**).

This seamless integration of Python tools within the R environment allows researchers to harness the collective strengths of both ecosystems, enabling them to tackle complex analytical challenges and unravel the intricate mechanisms governing cellular processes at the single-cell level.

Enhanced Data Visualization with Aesthetic Refinement

Effective visualization is paramount in the field of single-cell transcriptomics, enabling the communication of intricate cellular patterns, facilitating data interpretation, and conveying scientific insights. SeuratExtend recognizes the importance of aesthetics in scientific communication and introduces an array of tools to enhance the visual appeal and clarity of data representations.

While offering advanced analytical capabilities, SeuratExtend also places great emphasis on optimizing and expanding the fundamental visualization functions essential for presenting complex analyses. The package provides improved versions of core visualization tools such as heatmaps, dimensional reduction (UMAP) plots, violin/box plots, cluster proportion bar plots, dot/bubble plots, waterfall plots, and volcano plots (**Figures 1, 2A, 2B, 3E, and 3F**). SeuratExtend also continues to expand its visualization capabilities in response to user needs. These enhancements streamline the visualization process and introduce new features and

customization options, such as convenient statistical annotation and layout settings, enabling researchers to create more informative and visually compelling representations of their data.

Central to SeuratExtend's approach to visualization is the implementation of thoughtfully curated color schemes that adhere to principles of effective data science plotting. The creation of the "professional discrete color" (*color_pro*) series stems from the understanding that choosing the right colors for scientific visualizations is critical. Colors must be distinct enough to differentiate data points clearly, yet coordinated and subdued enough to maintain professionalism and avoid visual strain.

In the realm of data science visualization, certain color choices should be avoided, such as monochromatic schemes that can reduce visual distinction (**Figure 4A**), causing data points to blend together. Similarly, overly saturated colors can be visually aggressive and distracting, detracting from the scientific message (**Figure 4A**). While certain vibrant schemes might be engaging in an advertising context, they may be considered informal for professional journal standards (**Figure 4B**).

The *color_pro* series addresses these concerns by offering a collection of seven meticulously crafted color schemes: *default*, *light*, *red*, *yellow*, *green*, *blue*, and *purple* (**Figures 4C and 4D**). These palettes are generated using *I Want Hue* [18], an optimized algorithm that ensures visually pleasing and distinctly separable color combinations, with carefully adjusted parameters to align with the aforementioned principles. The *default* and *light* schemes span the entire hue domain, catering to general use cases while accommodating different background and text color requirements (**Figure 4C**). The specialized color schemes (*red*, *yellow*, *green*, *blue*, and *purple*) offer harmonious hues confined to specific regions (**Figure 4D**),

enabling vibrant yet coordinated visualizations that reflect the biological or categorical properties of the data.

In addition to the curated color schemes, SeuratExtend introduces an innovative approach to visualize multiple features simultaneously on a single dimension reduction plot, such as UMAP. This novel method utilizes the principles of color mixing in either the RGB (red, green, blue) or RYB (red, yellow, blue) color systems to represent the levels of different features, such as gene expression, pathway AUCell scores, or transcription factor activities.

In the RGB system, black represents no or low expression, while brighter colors indicate higher levels (**Figure 4E**). This color mixing approach is straightforward and well-established in the digital color space. In contrast, the RYB system, which is more intuitive and closely mimics the subtractive color mixing used in traditional art, employs white to represent no expression, with deeper colors indicating higher expression levels (**Figure 4F**).

However, designing an algorithm for RYB color mixing that accurately simulates real-world color blending poses a challenge. Moreover, the pure primary colors in the RYB system have inherent limitations for data visualization purposes, as yellow tends to be too light and blue too dark. To address these issues, SeuratExtend has developed a custom algorithm for RYB color mixing and has fine-tuned the brightness and saturation of the primary colors to optimize them for data visualization (**Figure 4F**).

In summary, SeuratExtend equips researchers to create visually compelling and scientifically rigorous representations of complex biological insights by integrating innovative color schemes, multi-feature visualization capabilities, and intuitive color mixing principles into its optimized plotting tools.

Utility Toolset: Streamlining Analysis Workflows

SeuratExtend introduces a comprehensive suite of utility tools designed to streamline and enhance scRNA-seq data analysis workflows (**Figure 1**). A recurring challenge in scRNA-seq analysis involves reconciling gene identifiers across disparate databases and organisms. SeuratExtend addresses this by providing robust functions that facilitate gene naming conversions between human and mouse gene symbols, Ensembl IDs, and UniProt accession numbers. These functions utilize localized databases, ensuring reliable and efficient conversion while mitigating instability issues associated with online resources. Users can directly convert gene expression matrices between human and mouse counterparts, streamlining cross-species analyses. For scenarios requiring online resources, SeuratExtend provides flexibility to fetch results directly from BioMart databases.

SeuratExtend introduces convenient tools for computing statistics and assessing the proportion of positive cells within clusters or groups. One tool computes various metrics, including mean, median, z-scores, or log-fold changes, for genomic data stored in Seurat objects or standard matrices. This enables researchers to identify cluster-specific gene expression patterns or pathway activities, crucial for understanding cellular heterogeneity and functional characteristics. Another tool allows users to assess the proportion of positive cells expressing a particular feature within specified clusters or groups, enabling the identification of genes or pathways exhibiting significant expression levels within subpopulations of cells.

To further enhance accessibility and reproducibility, SeuratExtend introduces a function that automates the execution of a standard Seurat pipeline, including normalization, principal component analysis (PCA), clustering, and uniform manifold approximation and projection (UMAP) visualization. This function offers extensive customization options and intelligent

conditional execution, ensuring that specific steps are re-run only when necessary. Additionally, it provides the option to integrate and correct for batch effects using the Harmony algorithm. According to benchmarking studies [24], Harmony performs well in general tasks, offering fast computation and efficient resource utilization while maintaining simplicity of use, making it an excellent choice for batch effect correction in scRNA-seq data analysis.

SeuratExtend's utility toolset represents a valuable advancement in streamlining and enhancing scRNA-seq data analysis workflows, facilitating efficient navigation of the complexities of scRNA-seq data exploration and accelerating scientific discoveries.

Novel Applications of SeuratExtend in Pathway-Level Analysis and Cluster Annotation

The SeuratExtend framework offers innovative approaches to address fundamental challenges in single-cell transcriptomic data analysis, such as cellular heterogeneity interpretation and cluster annotation. By leveraging the package's comprehensive integration of pathway databases and analytical tools, researchers can explore novel strategies to gain deeper insights into the functional characteristics of cell populations and streamline the annotation process.

Exploring and Analyzing Single-Cell Data at the Pathway Level

SeuratExtend introduces a novel approach to dimensionality reduction and clustering by harnessing its extensive integration of pathway databases, such as Gene Ontology (GO), Reactome, KEGG, and BioCarta. Instead of relying solely on gene expression-cell matrices, this method transforms the data into pathway enrichment score-cell matrices using AUCell. Subsequent analyses, including PCA and clustering, are then performed on these pathway-level matrices. This pathway-based approach offers several advantages. First, clustering based on pathway-level information rather than individual genes greatly reduces the impact of gene

expression fluctuations. Second, by focusing on curated pathways containing functionally characterized genes, the influence of sample-specific genes with unknown functions, such as non-coding RNAs and pseudogenes, is minimized.

To illustrate the potential of this approach, we present an example using scRNA-seq data from a melanoma cohort [25]. When clustering is based on gene expression, the UMAP visualization is significantly influenced by sample origin (**Figure 5A**). In contrast, clustering based on pathway enrichment scores derived from multiple databases (GO, Reactome, KEGG, and BioCarta) effectively aggregates cells with conserved features, such as T cells and B cells, while preserving the biological variations in malignant cells across samples (**Figure 5B**). Importantly, this pathway-based analysis can provide novel functional insights that may be difficult to obtain through traditional gene-based methods. For instance, in the original publication of the melanoma dataset, the authors identified two clusters of malignant cells named "patient_specific_A" and "patient_specific_B" but could not infer their functional characteristics based on marker genes alone. By leveraging SeuratExtend's pathway-based approach, we can easily identify the specific pathways that distinguish these clusters (**Figure 5C**), thereby gaining a deeper understanding of their biological roles.

SeuratExtend's pathway-based approach is highly versatile, as it can be applied to various databases, as well as customized pathway databases, enabling fine-tuned clustering for specific datasets. However, it should be acknowledged that this method shares some of AUCell's limitations, such as sensitivity to the number of genes expressed per cell (nGene) and optimal performance within certain gene set size ranges. Consequently, substantial differences in nGene across samples may impact the computational results.

Semi-Automated Cluster Annotation with Signature Enrichment Analysis

SeuratExtend also facilitates semi-automated cluster annotation without the need for additional tools like SingleR [26]. By leveraging databases such as PanglaoDB [27], which contains marker genes for over 100 cell types, researchers can calculate AUCell scores to identify enriched cell type signatures within each cluster, greatly assisting the annotation process.

In the provided example, unsupervised clustering yields nine unnamed clusters (**Figure 5D**). By calculating AUCell scores using PanglaoDB and sorting the top cell type signatures for each cluster, researchers can efficiently annotate the clusters based on this information (**Figure 5D**).

In summary, SeuratExtend's novel applications in pathway-level analysis and cluster annotation demonstrate its potential to address critical challenges in scRNA-seq data interpretation. By leveraging integrated pathway databases and signature enrichment analysis, SeuratExtend enables researchers to gain deeper insights into the functional characteristics of cell populations, streamline the annotation process, and ultimately expand the analytical horizons of single-cell transcriptomics.

Educational Impact

We recently demonstrated SeuratExtend's educational value by designing a comprehensive scRNAseq analysis teaching module based on it. The course successfully transitioned complete beginners to competent analysts and underlines SeuratExtend's accessibility and intuitive design. Structured as six progressive lessons, the curriculum began with fundamental R programming concepts and gradually advanced to sophisticated analytical techniques. This systematic approach enabled participants to build confidence with basic operations before tackling more complex analyses. Notably, the course expanded beyond SeuratExtend's core

functionality to cover essential advanced topics in single-cell analysis, including doublet removal, cell-cell communication analysis, automated cluster annotation, TCR/BCR analysis, and copy number variation inference. This comprehensive coverage ensured participants to gain a deeper understanding of the single-cell analysis landscape while learning to effectively utilize SeuratExtend's streamlined workflow.

Following the workshop's success and responding to community interest, all course materials have been made freely available online, extending SeuratExtend's educational impact beyond the initial workshop. This development marks a significant step in democratizing single-cell analysis education, making sophisticated analytical techniques accessible to a broader audience of researchers while positioning SeuratExtend as a valuable educational resource in the field of single-cell genomics.

Comprehensive Tutorials and Interactive Learning Resources

SeuratExtend prioritizes user accessibility through its extensive documentation and interactive learning resources. The package's primary documentation [28], provides detailed function descriptions, practical examples, and comprehensive workflow guides. This documentation is continuously refined based on user feedback and common questions, ensuring its relevance and utility for users at all skill levels.

To further enhance the learning experience, SeuratExtend incorporates artificial intelligence through a specialized chatbot powered by large language models. This interactive assistant, trained on the package's documentation and tutorials, provides real-time support for users encountering technical challenges or seeking guidance on analytical approaches. The chatbot

complements traditional documentation by offering personalized assistance and helping users navigate the complexities of single-cell analysis.

The integration of these educational resources—comprehensive documentation, interactive support, and structured tutorials—creates a robust learning ecosystem that supports users from their initial encounter with single-cell analysis through to advanced applications. This systematic approach to user education and support distinguishes SeuratExtend within the single-cell analysis toolkit landscape, making sophisticated analytical techniques more accessible to researchers across different experience levels.

Evolving Applications of SeuratExtend: From Early Adoption to Current Capabilities

SeuratExtend originated from practical research needs, with its capabilities expanding through real-world application. Here, we highlight specific instances where the package facilitated discoveries in two diverse research domains, demonstrating its practical value and subsequent evolution.

In a study investigating tumor-associated high-endothelial venules (TU-HEVs) [29]. SeuratExtend's integrated workflow enabled a comprehensive multi-angle analysis approach that would have been challenging to implement with fragmented tools. The researchers conducted extensive comparative analyses between TU-HEVs, lymph node HEVs (LN-HEVs), and tumor ECs (TU-ECs) across both murine and human samples. This comprehensive analysis integrated multiple trajectory inference methods in Python and R, pathway enrichment analyses, and transcription factor network reconstruction within a unified framework. The ability to seamlessly transition between these analytical perspectives was crucial for identifying the most promising directions for experimental validation, ultimately confirming that TU-HEVs

arise from the metaplastic conversion of postcapillary venules (PCVs). Similarly, in a subsequent study of systemic autoinflammatory diseases (SAIDs) [30], this integrated approach facilitated rapid cross-disciplinary analysis, combining GSEA with trajectory analysis tools like scVelo and Palantir to quickly uncover an unexpected therapeutic mechanism. Rather than simply suppressing inflammatory cytokines, anti-TNF therapy was found to alter macrophage differentiation pathways—an insight that emerged from the ability to efficiently investigate multiple analytical perspectives in parallel.

These applications demonstrate how SeuratExtend's integration of diverse analytical methods within a unified framework can facilitate biological discoveries by enabling more comprehensive data exploration. Since these initial applications, SeuratExtend has undergone significant development based on user feedback. Many functions have been optimized for computational efficiency and compatibility with different R environments. The current version represents a substantial advancement, offering a comprehensive, integrated platform for single-cell RNA-seq analysis, supported by extensive documentation and tutorials to lower the entry barrier for researchers new to the field.

DISCUSSION

SeuratExtend represents a significant advancement in the field of single-cell RNA sequencing (scRNA-seq) analysis, addressing the critical challenges that have emerged from the rapid proliferation of computational tools and algorithms in this domain. Our comprehensive evaluation and integration of essential analytical components have resulted in a robust, user-friendly framework that streamlines complex workflows and enhances the accessibility of advanced scRNA-seq analysis techniques.

The development of SeuratExtend was guided by three core principles: integration, intuitive design, and visual aesthetics. By incorporating multiple databases, analytical tools, and visualization techniques, we have created a cohesive ecosystem that bridges the gap between R and Python environments. This integration not only simplifies the analytical process but also expands the repertoire of available tools for researchers working primarily in R. The intuitive design of SeuratExtend, featuring straightforward functions and extensive documentation, significantly lowers the entry barrier for both novice and experienced users. Furthermore, our emphasis on visual aesthetics, exemplified by the carefully curated color schemes and optimized visualization methods, enhances the clarity and impact of data representation in scientific communications.

SeuratExtend provides a comprehensive suite of analytical tools, encompassing essential components such as denoising, batch integration, pathway analysis, gene regulatory network inference, and trajectory analysis. While the single-cell field continues to expand with new methodologies and tools, SeuratExtend adopts a strategic approach to tool integration, recognizing that reimplementing every available method within a single package is neither practical nor necessarily beneficial to users. Many existing tools already offer excellent Seurat compatibility, making redundant integration unnecessary. Instead, SeuratExtend focuses on ensuring robust implementation and reliable maintenance of its integrated functionalities while providing comprehensive educational resources and guidance for additional analytical approaches such as doublet removal, cell-cell communication, copy number variation inference, and automated cluster annotation through its extensive tutorial system. This approach ensures that users not only have access to well-maintained analytical tools but also

gain the knowledge and confidence to effectively utilize the broader ecosystem of single-cell analysis methods.

To realize this potential, SeuratExtend's future development and expansion will be greatly enhanced by active community engagement. As with many successful open-source projects, user feedback and contributions from the scientific community will play a crucial role in shaping the package's trajectory. This collaborative approach ensures that SeuratExtend continues to evolve in alignment with users' needs and the rapidly advancing field of single-cell genomics. We encourage users to provide feedback, suggest new features, and contribute to the codebase, fostering a vibrant ecosystem that can adapt to emerging challenges and opportunities in scRNA-seq analysis.

Looking ahead, SeuratExtend's integration and intuitive nature position it as an excellent educational resource, further lowering the entry barrier for those eager to learn single-cell analysis. Additionally, the rise of large language models (LLMs) presents an opportunity for AI-assisted education. While SeuratExtend currently uses OpenAI's platform for a chatbot, future endeavors may involve building more versatile chatbots using frameworks like Langchain and other LLMs like Claude and Llama 3, increasing accessibility and reducing costs. Furthermore, SeuratExtend's standardized data analysis and visualization framework could pave the way for visual applications like Shiny apps, making scRNA-seq analysis accessible even to non-bioinformaticians.

In conclusion, SeuratExtend represents a significant stride in streamlining scRNA-seq data analysis, offering a focused and integrated solution built upon the Seurat framework. By addressing the challenges of tool proliferation and complexity while maintaining a clear emphasis on stability and user accessibility, SeuratExtend has made advanced scRNA-seq

analysis more accessible to a broader range of researchers. The package's success in educational settings and positive user feedback demonstrate its effectiveness in bridging the gap between sophisticated analytical capabilities and practical usability.

Availability of Source Code and Requirements

Project name: SeuratExtend

Project home page: https://github.com/huayc09/SeuratExtend

Operating system(s): Linux/Windows/MacOS

Programming language: R

Other requirements: R (⩾ 3.6), Seurat, dplyr, ggplot2, reticulate

License: GNU General Public License (GPL) version 3 or later. Data files (*.rda files) are released under CC0 1.0 Universal Public Domain Dedication.

RRID: SCR_026143

WorkflowHub [31]: 10.48546/workflowhub.workflow.1385.1

Software Heritage PID [32]:

swh:1:snp:815e826cdf8c4f4985308551b779b82e45895c81;origin=https://github.com/huayc09/SeuratExtend

Data Availability

The SeuratExtend package is freely available in the GitHub repository (https://github.com/huayc09/SeuratExtend) and has been deposited to figshare [33]. A version of record snapshot of the GitHub repository has been archived in the Software Heritage Library

with the PID swh:1:snp:815e826cdf8c4f4985308551b779b82e45895c81;origin=https://github.com/huayc09/SeuratExtend [32]. The repository also contains comprehensive documentation and tutorials (https://huayc09.github.io/SeuratExtend/) to help users get started with the package and understand its functionalities. The example datasets used in the tutorials are available on Zenodo [34]. SeuratExtend has been registered in WorkflowHub [31]. SeuratExtend Chatbot (beta version, powered by ChatGPT): https://chatgpt.com/g/g-8scQjmzkd-scrna-seq-assistant

List of Abbreviations

DEGs: Differentially Expressed Genes

GO: Gene Ontology

GSEA: Gene Set Enrichment Analysis

MAGIC: Markov Affinity-based Graph Imputation of Cells

PCA: Principal Component Analysis

SCENIC: Single-Cell Regulatory Network Inference and Clustering

scRNA-seq: Single-cell RNA sequencing

UMAP: Uniform Manifold Approximation and Projection

Author Contributions

Y.H. conceived the idea, designed the study, developed the SeuratExtend package, performed data analysis, and drafted the manuscript. F.R. supervised the project and provided guidance throughout its development. L.W. provided extensive feedback on the package and reported

bugs, contributing to its overall improvement. F.Z. assisted in testing the code and tutorials. All authors read and approved the final manuscript.

Acknowledgements

Y.H. would like to express his sincere gratitude to the VIB KU-Leuven for their single-cell RNA-seq course, which provided the foundational knowledge essential for the development of SeuratExtend. Special thanks go to the individuals from the University Hospital Essen, KU Leuven-VIB Center for Cancer Biology, Institut National de la Santé et de la Recherche Médicale (Inserm), and Max Delbrück Center for Molecular Medicine (MDC) who contributed their time and effort to test the package. The author also acknowledges the assistance of Claude and ChatGPT in refining the code and manuscript language.

Funding

F.R. is funded by the Melanoma Research Alliance and the Wolfgang & Gertrud Boettcher Foundation. Y.H. is funded by the Melanoma Research Alliance. F.Z. received funding from the Else-Kröner-Fresenius Stiftung (Else Kröner Medical Scientist Kolleg, UMESciA, 2021_EKMK.12)

Conflict Of Interest

The authors declare no conflict of interest.

References

1. Zappia L, Theis FJ. Over 1000 tools reveal trends in the single-cell RNA-seq analysis landscape. *Genome Biol*. Springer Science and Business Media LLC; 2021; doi: 10.1186/s13059-021-02519-4.

2. Luecken MD, Theis FJ. Current best practices in single‐cell RNA‐seq analysis: a tutorial. *Mol Syst Biol*. Springer Science and Business Media LLC; 2019; doi: 10.15252/msb.20188746.

3. Heumos L, Schaar AC, Lance C, Litinetskaya A, Drost F, Zappia L, et al.. Best practices for single-cell analysis across modalities. *Nat Rev Genet*. Springer Science and Business Media LLC; 2023; doi: 10.1038/s41576-023-00586-w.

4. Kharchenko PV. The triumphs and limitations of computational methods for scRNA-seq. *Nat Methods*. Springer Science and Business Media LLC; 2021; doi: 10.1038/s41592-021-01171-x.

5. Hao Y, Hao S, Andersen-Nissen E, Mauck WM 3rd, Zheng S, Butler A, et al.. Integrated analysis of multimodal single-cell data. *Cell*. Elsevier BV; 2021; doi: 10.1016/j.cell.2021.04.048.

6. Wolf FA, Angerer P, Theis FJ. SCANPY: large-scale single-cell gene expression data analysis. *Genome Biol*. Springer Science and Business Media LLC; 2018; doi: 10.1186/s13059-017-1382-0.

7. Virshup I, Bredikhin D, Heumos L, Palla G, Sturm G, Gayoso A, et al.. The scverse project provides a computational ecosystem for single-cell omics data analysis. *Nat Biotechnol*. Springer Science and Business Media LLC; 2023; doi: 10.1038/s41587-023-01733-8.

8. The Gene Ontology Consortium. The Gene Ontology Resource: 20 years and still GOing strong. *Nucleic Acids Res*. Oxford University Press (OUP); 2019; doi: 10.1093/nar/gky1055.

9. Gillespie M, Jassal B, Stephan R, Milacic M, Rothfels K, Senff-Ribeiro A, et al.. The reactome pathway knowledgebase 2022. *Nucleic Acids Res*. Oxford University Press (OUP); 2022; doi: 10.1093/nar/gkab1028.

10. Bergen V, Lange M, Peidli S, Wolf FA, Theis FJ. Generalizing RNA velocity to transient cell states through dynamical modeling. *Nat Biotechnol*. Springer Science and Business Media LLC; 2020; doi: 10.1038/s41587-020-0591-3.

11. Lange M, Bergen V, Klein M, Setty M, Reuter B, Bakhti M, et al.. CellRank for directed single-cell fate mapping. *Nat Methods*. 2022; doi: 10.1038/s41592-021-01346-6.

12. Setty M, Kiseliovas V, Levine J, Gayoso A, Mazutis L, Pe'er D. Characterization of cell fate probabilities in single-cell data with Palantir. *Nat Biotechnol*. Springer Science and Business Media LLC; 2019; doi: 10.1038/s41587-019-0068-4.

13. Aibar S, González-Blas CB, Moerman T, Huynh-Thu VA, Imrichova H, Hulselmans G, et al.. SCENIC: single-cell regulatory network inference and clustering. *Nat Methods*. 2017; doi: 10.1038/nmeth.4463.

14. The Gene Ontology Consortium. Gene Ontology Database. https://geneontology.org/. Accessed December 2024.

15. Reactome. Reactome Pathway Database. https://reactome.org/. Accessed December 2024.

16. GSEA Molecular Signatures Database. https://www.gsea-msigdb.org/. Accessed December 2024.

17. Franzén O, Gan LM, Björkegren JLM. PanglaoDB Database. https://panglaodb.se/. Accessed December 2024.

18. MediaLab. I Want Hue Color Tool. http://medialab.github.io/iwanthue/. Accessed December 2024.

19. UniProt Consortium. UniProt Database. https://www.uniprot.org/. Accessed December 2024.

20. Zhang H. SCP: Single Cell Pipeline (Version 0.5.6) https://github.com/zhanghao-njmu/scp. Accessed December 2024.

21. Noureen N, Ye Z, Chen Y, Wang X, Zheng S. Signature-scoring methods developed for bulk samples are not adequate for cancer single-cell RNA sequencing data. *Elife*. 2022; doi: 10.7554/eLife.71994.

22. van Dijk D, Sharma R, Nainys J, Yim K, Kathail P, Carr AJ, et al.. Recovering Gene Interactions from Single-Cell Data Using Data Diffusion. *Cell*. 2018; doi: 10.1016/j.cell.2018.05.061.

23. Galuzzi BG, Vanoni M, Damiani C. Combining denoising of RNA-seq data and flux balance analysis for cluster analysis of single cells. *BMC Bioinformatics*. 2022; doi: 10.1186/s12859-022-04967-6.

24. Yu X, Xu X, Zhang J, Li X. Batch alignment of single-cell transcriptomics data using deep metric learning. *Nat Commun*. 2023; doi: 10.1038/s41467-023-36635-5.

25. Pozniak J, Pedri D, Landeloos E, Van Herck Y, Antoranz A, Vanwynsberghe L, et al.. A TCF4-dependent gene regulatory network confers resistance to immunotherapy in melanoma. *Cell*. 2024; doi: 10.1016/j.cell.2023.11.037.

26. Dvir Aran, Aaron Lun, Daniel Bunis, Jared Andrews, Friederike Dündar. SingleR. Bioconductor; doi: 10.18129/B9.bioc.SingleR

27. Franzén O, Gan L-M, Björkegren JLM. PanglaoDB: a web server for exploration of mouse and human single-cell RNA sequencing data. *Database (Oxford)*. Oxford University Press (OUP); 2019; doi: 10.1093/database/baz046.

28. Hua Y. SeuratExtend Documentation and Tutorials. https://huayc09.github.io/SeuratExtend/. Accessed December 2024.

29. Hua Y, Vella G, Rambow F, Allen E, Antoranz Martinez A, Duhamel M, et al.. Cancer immunotherapies transition endothelial cells into HEVs that generate TCF1+ T lymphocyte niches through a feed-forward loop. *Cancer Cell*. 2022; doi: 10.1016/j.ccell.2022.11.002.

30. Hua Y, Wu N, Miao J, Shen M. Single-cell transcriptomic analysis in two patients with rare systemic autoinflammatory diseases treated with anti-TNF therapy. *Front Immunol*. 2023; doi: 10.3389/fimmu.2023.1091336.

31. Hua, Y. (2025). SeuratExtend. WorkflowHub. https://doi.org/10.48546/WORKFLOWHUB.WORKFLOW.1385.1

32. Hua Y, Weng L, Zhao F and Rambow F. (2025) SeuratExtend: An Enhanced Toolkit for scRNA-seq Analysis (Version 1). [Computer software]. Software Heritage, https://archive.softwareheritage.org/swh:1:snp:815e826cdf8c4f4985308551b779b82e45895c81;origin=https://github.com/huayc09/SeuratExtend

33. Hua Y, Weng L, Zhao F and Rambow F. (2025) SeuratExtend (Version 1). [Computer software]. figshare. https://doi.org/10.6084/m9.figshare.26264255.v1

34. Hua Y, Weng L, Zhao F and Rambow F. (2025). SeuratExtend Tutorial: Curated Example Datasets for Single-Cell Analysis. Zenodo. https://doi.org/10.5281/zenodo.10944065

Table And Figures Legends

Figure 1. Overview of the SeuratExtend package's key features.

SeuratExtend streamlines single-cell RNA-seq data analysis by integrating essential components into the Seurat framework: (1) Functional and Pathway Analysis (GSEA) with multiple databases and AUCell algorithm; (2) Python Tool Integration for trajectory analysis (scVelo, Palantir, CellRank), gene regulatory network inference (SCENIC), and denoising (MAGIC); (3) Enhanced Visualization with optimized methods and professional color schemes; and (4) Utility Functions for gene identifier conversion, batch processing, and statistical analysis.

Figure 2. Visualizations of gene set enrichment analysis (GSEA) results using SeuratExtend.

(A) Heatmap displaying the z-scores of immune-related GO terms across different cell types or conditions. (B) Violin plots showing the distribution of GSEA scores for the selected GO term across different cell types, with asterisks indicating statistically significant differences (**** p <

0.001). (C) GSEA plot for the Hallmark IFN gamma Response gene set, comparing CD4 T naive cells to non-CD4 T naive cells. Kolmogorov-Smirnov Test.

Figure 3. Trajectory analysis, pseudotime inference, denoising, and gene regulatory network visualization using SeuratExtend's integrated Python tools.

(A) RNA velocity analysis using scVelo, displaying velocity vectors on a UMAP embedding. (B) Diffusion map and pseudotime calculation using Palantir, comparing DC, Mono CD14, and Mono FCGR3A cell populations. (C) Gene expression dynamics along the pseudotime trajectory for the CD14 and FCGR3A genes. (D) Heatmap showing the relative expression of fate marker genes (rows) along the pseudotime trajectory (columns) for fate1. (E) UMAP visualizations of CREB5 and POU2F2 expression before (CREB5, POU2F2) and after (magic_CREB5, magic_POU2F2) denoising with MAGIC, as well as the transcription factor activity AUCell score (tf_CREB5, tf_POU2F2) inferred by SCENIC. (F) Waterfall plot highlighting differential TF regulon activities between Mono CD14 and Mono FCGR3A cell populations, with the top 20 TFs labeled. (G) Gene regulatory networks predicted by SCENIC for Mono CD14 and Mono FCGR3A cell populations, with nodes colored by relative gene expression (round nodes) or regulon activity (square nodes).

Figure 4. Comparison of color schemes and their suitability for data science visualization.

(A) Not recommended color schemes: indistinct monochromatic colors that reduce visual distinction, and overly saturated colors that can be visually aggressive and distracting. (B) A lively color scheme that, while engaging, may be considered informal for professional journal

standards. (C) The "color_pro" series offers two meticulously crafted color schemes: "default" and "light", which span the entire hue domain and cater to general use cases. (D) The "color_pro" series also includes five specialized color schemes: "red", "yellow", "green", "blue", and "purple", which offer harmonious hues confined to specific regions, enabling vibrant yet coordinated visualizations that reflect the biological or categorical properties of the data. (E-F) Multi-feature visualization using color mixing principles in the RGB (E) or RYB (F) color system. The expression levels of three genes (CD3D, CD14, and CD79A) are displayed on a UMAP plot.

Figure 5. SeuratExtend's novel applications in pathway-level analysis and cluster annotation.

(A) UMAP visualization of melanoma scRNA-seq data colored by sample origin and clusters, demonstrating the influence of batch effects on gene expression-based clustering. (B) UMAP visualization of the same data colored by sample origin and clusters derived from pathway enrichment scores based on multiple databases (GO, Reactome, KEGG, and BioCarta), showing the grouping of cells with conserved features (e.g., T cells and B cells) while preserving biological variations in malignant cells. (C) UMAP visualization of malignant cells highlighting the location of patient_specific_A and patient_specific_B clusters (top) and their corresponding specific pathway activities (bottom). (D) UMAP visualization of unsupervised clustering results yielding nine unnamed clusters (top-left) and the corresponding heatmap of top enriched cell type signatures from PanglaoDB for each cluster (right), facilitating semi-automated cluster annotation (bottom-left).

Figure 1

Click here to access/download;Figure;Figure1.pdf

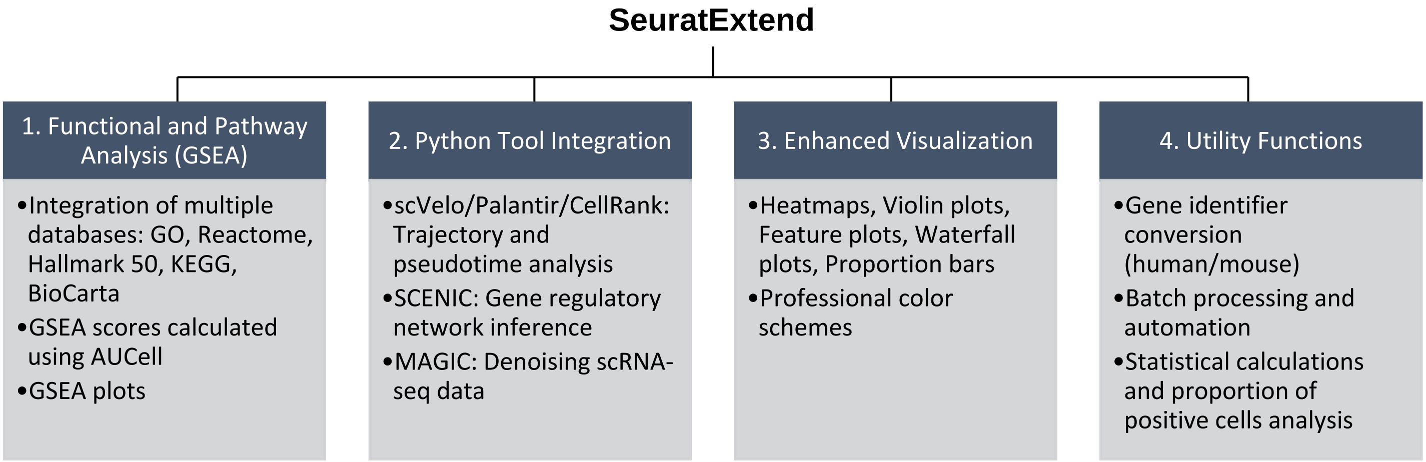

Figure 2

Click here to access/download;Figure;Figure2.pdf

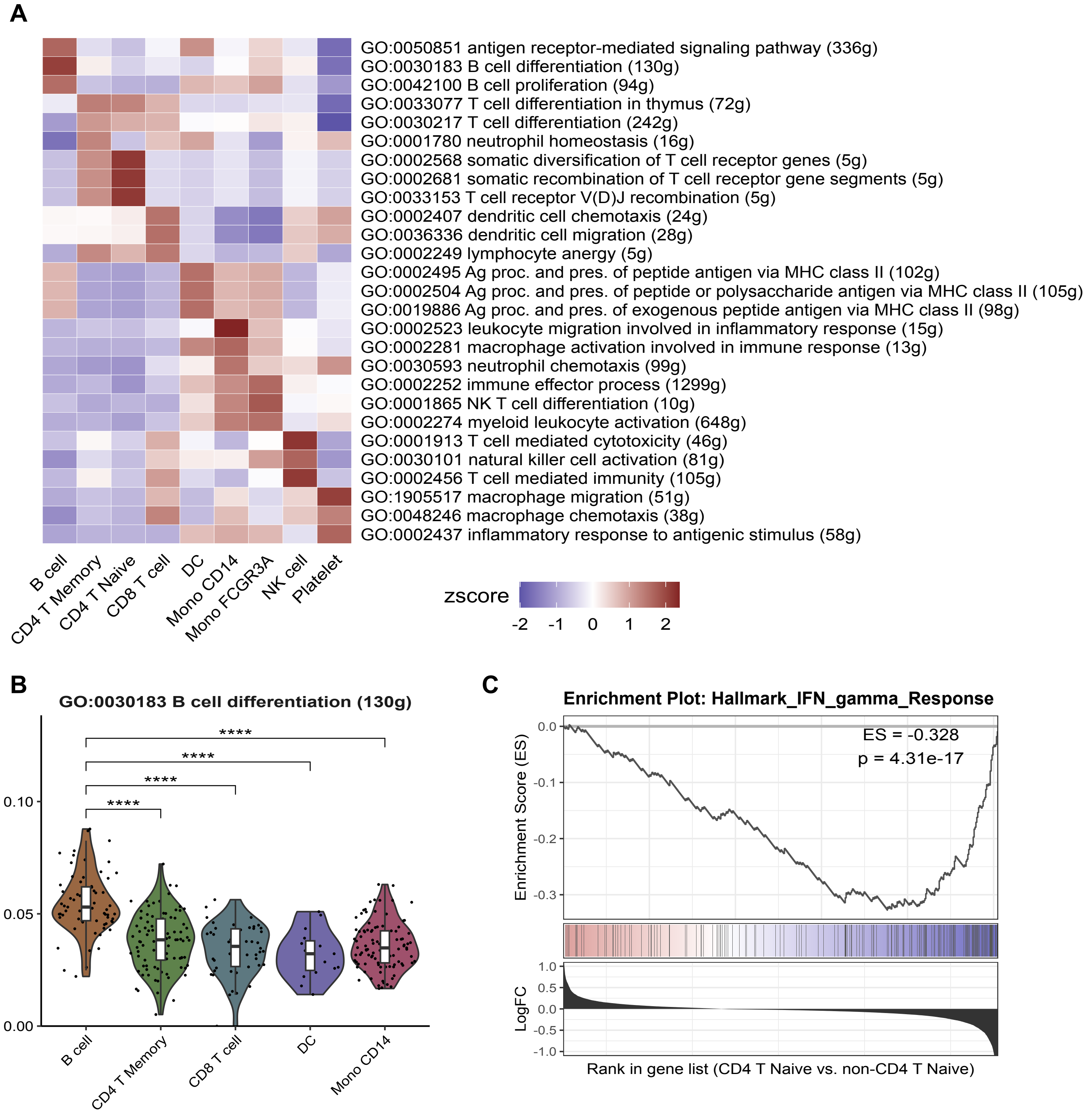

Figure 3

Click here to access/download;Figure;Figure3.pdf

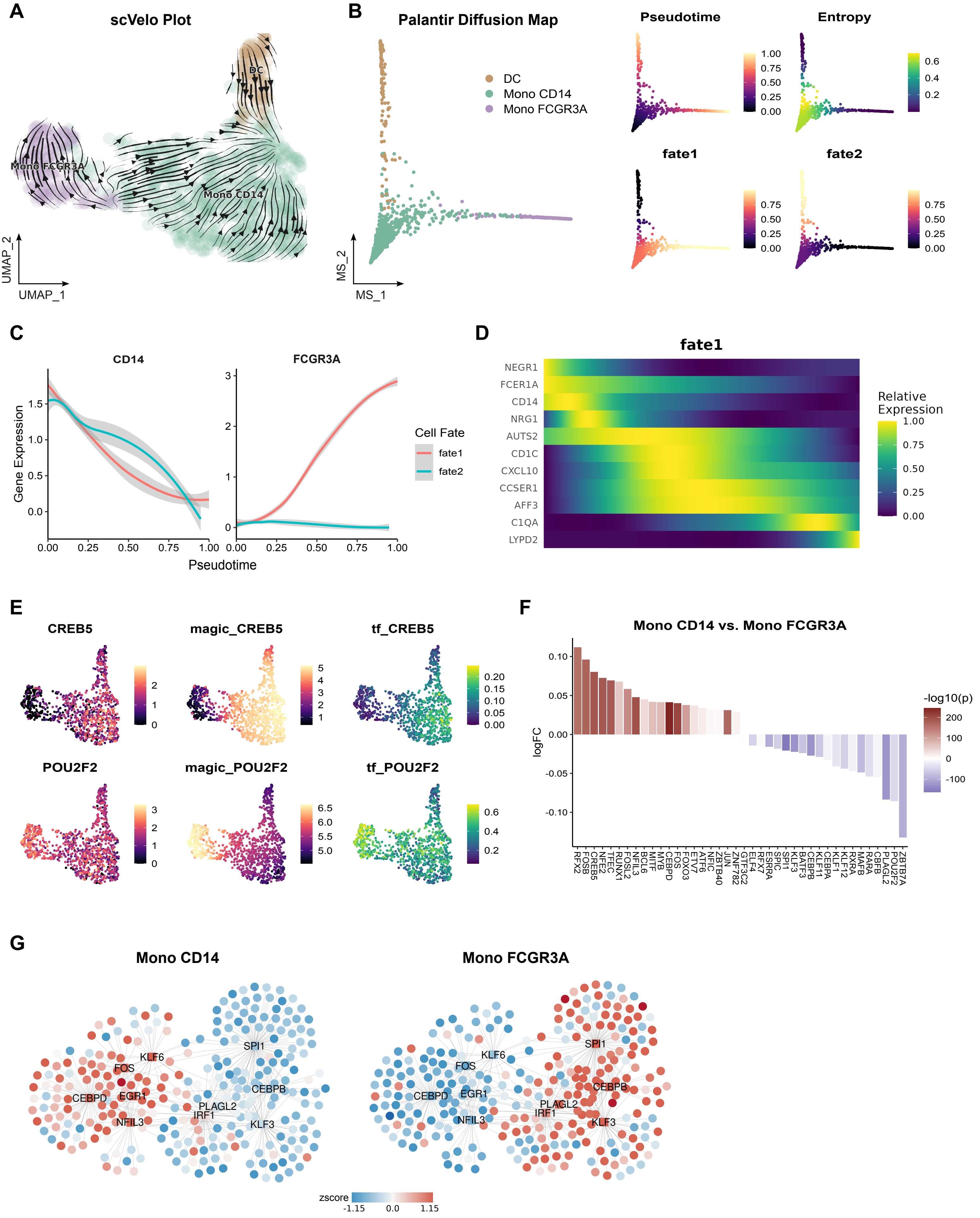

Figure 4 Click here to access/download;Figure;Figure4.pdf

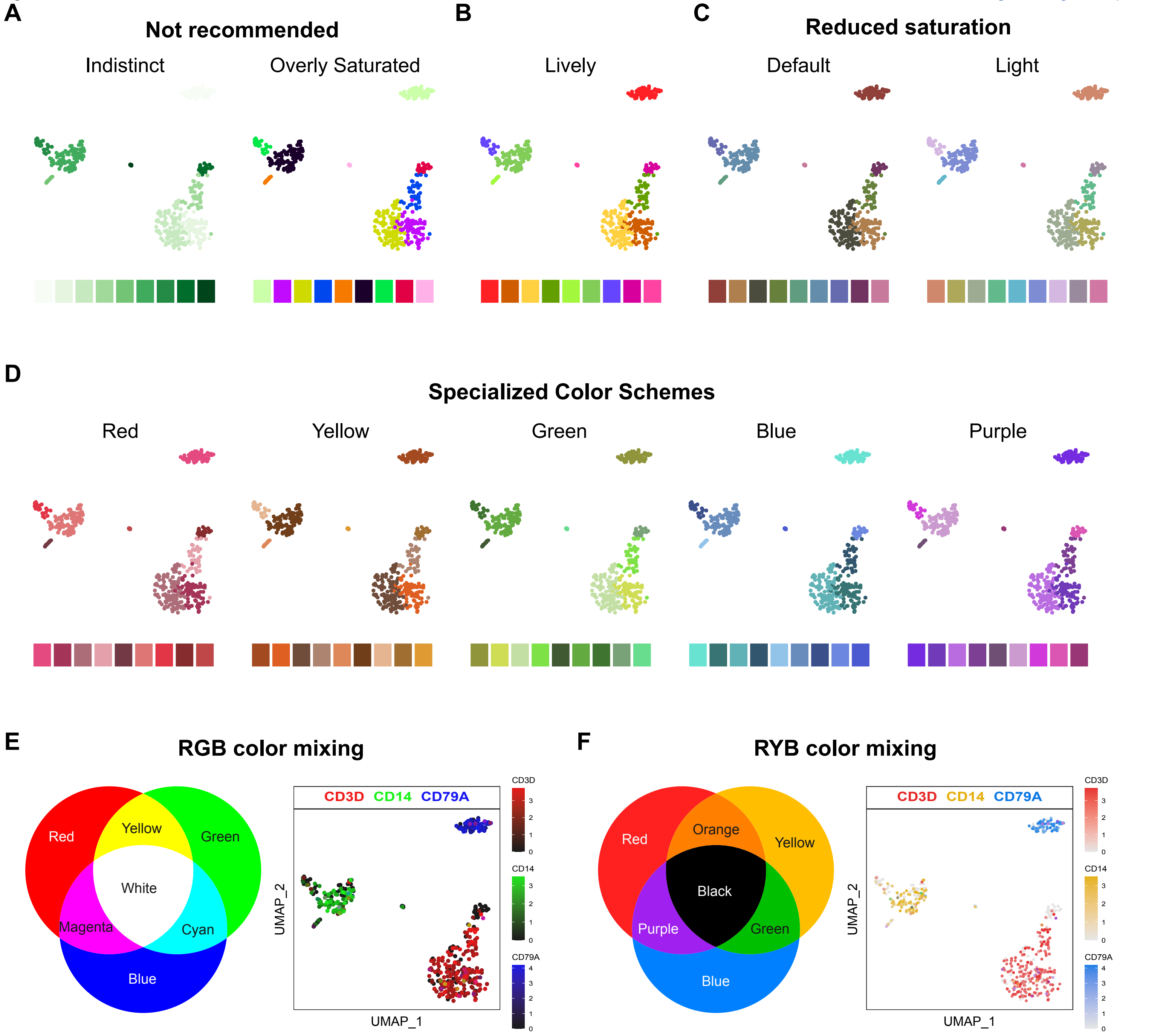

Figure 5

Click here to access/download;Figure;Figure5.pdf

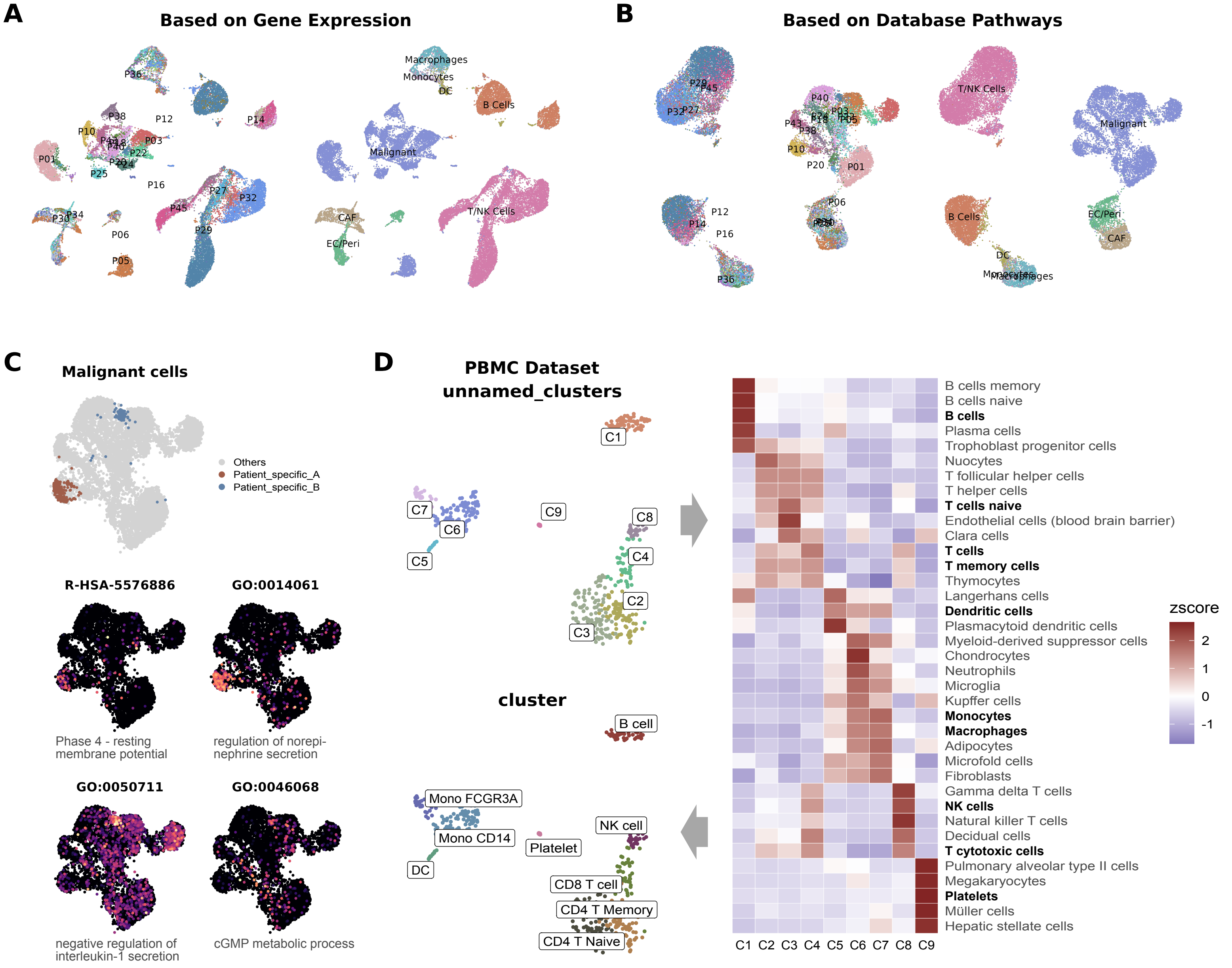

Cover Letter

Click here to access/download;Personal Cover;Cover_SeuratExtend_GigaScience_Rambow_Rebuttal.p

Universitätsmedizin Essen
Institut für KI in der Medizin (IKIM)

University Medicine Essen | IKIM | ACCR | Girardetstr. 8 | 45131 Essen, Germany

University Medicine Essen
Institute for Artificial Intelligence in Medicine (IKIM)
Girardetstr. 8
45131 Essen
Germany

Tel. +49 201 723–77813
E-Mail florian.rambow@uk-essen.de

Essen, 17. May 2025

Prof. Dr. Florian Rambow
Applied Computational Cancer Research

Dear Scott Edmunds,

It is a pleasure to re-submit our revised manuscript entitled "SeuratExtend: Streamlining Single-Cell RNA-Seq Analysis Through an Integrated and Intuitive Framework" (GIGA-D-24-00558). I am very appreciative of the referee's careful reading and constructive comments.

As you can see from the detailed point-by-point reply to the referees' comments we have addressed all of their points (see full rebuttal attached to this document).

Briefly, we implemented and improved several functions in our updated SeuratExtend v1.2.0 version relating to visualization (plotting), statistical analysis, and compatibility with latest Apple M4 chips. Furthermore, we carved out more convincingly SeuratExtend's innovative applications around Pathway-Analysis and Cluster Annotation. Finally, we shortened and streamlined voluminous text sections into concise and more comprehensive paragraphs.

As a result, our manuscript has further improved and I hope that you will find this new version acceptable for publication in *GigaScience* (manuscript type: "Technical Note").

Sincerely,

Prof. Dr. Florian Rambow
Applied Computational Cancer Research

Universitätsklinikum Essen
Hufelandstraße 55
45147 Essen

Tel. +49 (0)201 – 723 – 0
Web www.ume.de

Sparkasse Essen
IBAN DE82 3605 0105 0004 9007 00
BIC SPESDE3EXXX

Reviewer reports:

Reviewer #1: This manuscript introduces an extended version of the widely-used Seurat package, named SeuratExtend. Specifically, Hua et al. developed an integrated an intuitive framework to streamline scRNA-seq data analysis, such as trajectory analysis, GRN construction, and functional enrichment analysis. The package also features direct integration with other popular tools, including Seurat, scVelo, etc. Notably, the software has been demonstrated through training programs, with over 100 stars on GitHub, which is impressive. I have tested the package, including installation and some basic functions. Moreover, the GitHub webpage is well-documented, featuring multiple use cases tailored for beginners. The overall user experience exceeded my expectations, though I have a few minor comments for improvement:

1, The DimPlot2 function is very useful, and easy to customize the colors. However, the default color scheme seems to be too dark. Considering a more distinguishable and visually appealing color palette might be a solution.

We appreciate the reviewer's valuable feedback regarding the DimPlot2 function's default color scheme. This observation aligns with feedback from multiple users who found the default "dark" palette too intense for certain visualization contexts.

In response to this feedback, we have implemented several significant improvements in SeuratExtend v1.2.0:

- We have changed the default color scheme for discrete variables from "default" (dark) to "light" - a more visually comfortable palette with increased brightness and reduced saturation. This change makes plots more readable and visually appealing, particularly when labels are displayed.

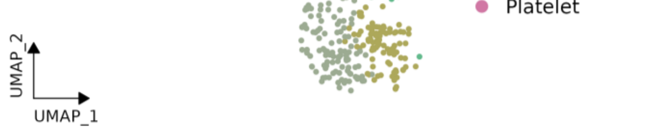

- We have introduced a new "bright" color scheme that offers higher contrast colors for visualizations requiring more distinct separation between groups. This option is particularly useful for presentations or when subtle color differences need to be emphasized.

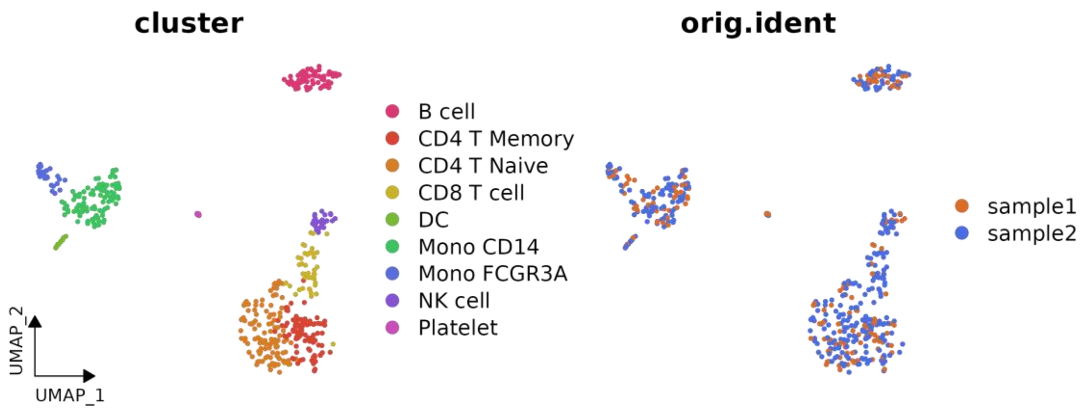

- We've expanded the maximum number of colors supported by our color palettes from 50 to 80, accommodating datasets with larger numbers of clusters or categories.

These enhancements provide users with greater flexibility in choosing color schemes that best suit their specific visualization needs while addressing the reviewer's concerns about distinguishability and visual appeal. The changes have been fully documented in our package vignettes with illustrative examples.

2, How to control the angles of cell type labels when using VlnPlot2? The 'Split visualization' has all the labels in a horizontal direction, leading to overlapping in some cases, while 'Subset Analysis' plots have labels in 45 degree, which is much better to read. However, I didn't see a parameter to control this. Does VlnPlot2 handle this automatically?

We agree with the reviewer's observation regarding label angles in VlnPlot2 visualizations. In response to this feedback, we have implemented a significant enhancement to the function in SeuratExtend v1.2.0.

Previously, the VlnPlot2 function automatically determined label angles based on label length (using 0 degrees for short labels and 45 degrees for longer ones), but this automatic adjustment was only available for non-split visualizations. The split visualization displayed labels horizontally, which could lead to overlapping text with longer labels as the reviewer correctly pointed out.

We have now added three new parameters to VlnPlot2:

- angle: Controls the rotation angle of labels
- hjust: Controls horizontal justification of labels
- vjust: Controls vertical justification of labels

All three parameters default to NULL, in which case the function automatically determines appropriate values based on label length and plot type:

- For non-split visualizations: 0 degrees for short labels (≤2 characters), 45 degrees for longer labels

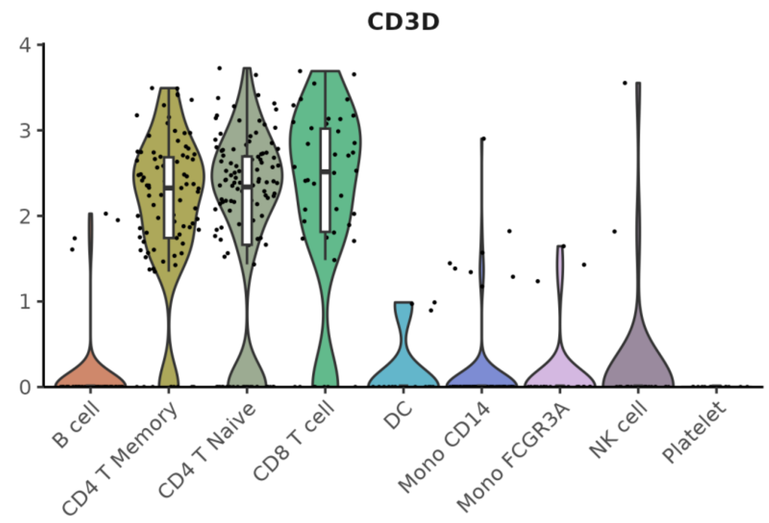

- For split visualizations: 0 degrees for short labels, -90 degrees (vertical) for longer labels

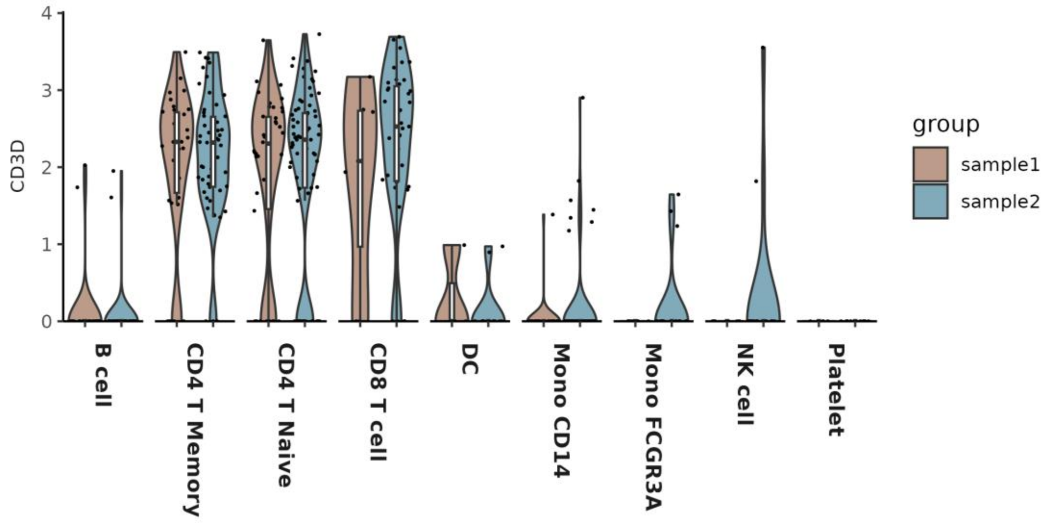

Users can also manually override these settings to achieve their preferred visualization style. The automatically determined hjust and vjust values ensure optimal text alignment based on the rotation angle.

This enhancement addresses the reviewer's concern while maintaining backward compatibility and providing additional flexibility for customization.

3, It's a very nice feature to have the 'Statistical Analysis' function to label significant groups. However, in single cell analysis, the p values are easy to be inflated due to the large number of cells. While the example pmbc data is relatively small, larger datasets might yield significant p values without obvious differences in the violin plots. It would be beneficial to mention this in the documentation, and provide some guidance so the results won't be misleading.

We appreciate the reviewer's insightful point regarding p-value inflation in single-cell analysis with the 'Statistical Analysis' function. This is indeed an important consideration that we've now addressed in SeuratExtend v1.2.0.

We have added explicit warnings about this issue in both the function documentation and the package vignette. Specifically, we now caution users that p-values can be artificially inflated in large single-cell datasets due to the high number of cells, potentially resulting in statistically significant differences (small p-values) even when the biological effect size is minimal.

The documentation now emphasizes that:

- Users should be cautious with statistical interpretations, especially when visual differences are subtle
- We recommend examining log fold changes (logFC) between groups to better assess the magnitude of biological differences
- Additionally, the percentage of cells expressing a marker (similar to pct.1 and pct.2 values in Seurat's FindMarkers) is an important metric to consider - if the difference in percentage between groups is minimal, the expression difference may be biologically negligible despite a significant p-value
- Parameters like min.pct in differential expression analysis can help filter out features with low expression prevalence
- For a comprehensive visualization of differences between two groups, our WaterfallPlot() function can provide logFC values along with statistical significance

- The function uses the Holm method (p.adjust.method = "holm") by default to adjust p-values for multiple comparisons

This warning appears in both the R documentation accessible via ?VlnPlot2 and in the "Enhanced Visualization" vignette, ensuring users are properly informed about this statistical consideration when interpreting their results.

We believe these additions directly address the reviewer's concern while providing practical guidance to users when applying statistical analysis to single-cell data.

4, The ClusterDistrBar is another valuable function. Based on my experience with similar analyses, I suggest incorporating features to identify robust changes in cell type composition. For instance, tools like sccomp can help determine changes in cell population composition.

We value the suggestion regarding enhancement of the *ClusterDistrBar* function to identify robust changes in cell type composition. We agree that this is an important aspect of single-cell analysis, especially when comparing multiple conditions or samples.

We have implemented a new function in SeuratExtend v1.2.0 called *ClusterDistrPlot*, which extends the functionality of *ClusterDistrBar*. This new function allows users to compare cluster distributions between experimental conditions using boxplots instead of stacked bars, and perform statistical testing (Wilcoxon rank-sum test or t-test) to identify significant differences in cell type proportions between conditions.

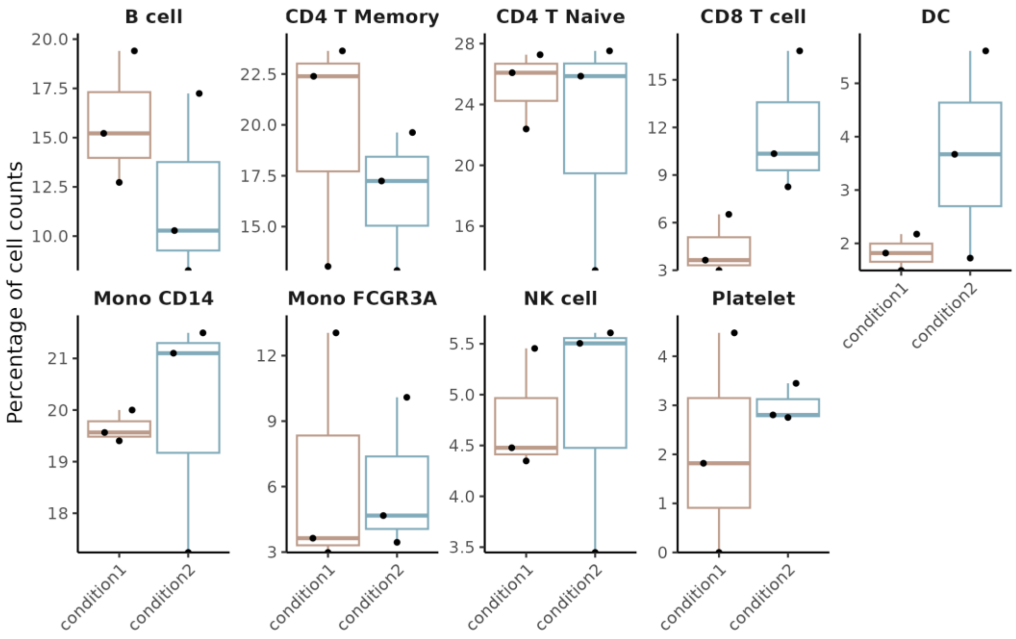

The *ClusterDistrPlot* function maintains backward compatibility with *ClusterDistrBar* while adding these new features specifically designed for compositional analysis. It applies various visualization parameters inherited from *VlnPlot2* for customized presentation. Our implementation was inspired by specialized tools like sccomp, maintaining the same intuitive interface and visualization style consistent with other SeuratExtend functions.

5, I wonder if the gene label directions can be changed easily for WaterfallPlot?

We welcome the reviewer's inquiry about changing gene label directions in the WaterfallPlot function. WaterfallPlot has included comprehensive label orientation controls through several parameters:

- The flip parameter controls the overall plot orientation (horizontal vs. vertical bars), with flip=TRUE displaying genes on the y-axis and flip=FALSE displaying genes on the x-axis.
- The angle parameter allows precise control over gene label rotation angles, defaulting to -90 degrees for vertical plots and 0 degrees for horizontal plots, but users can specify any custom angle.

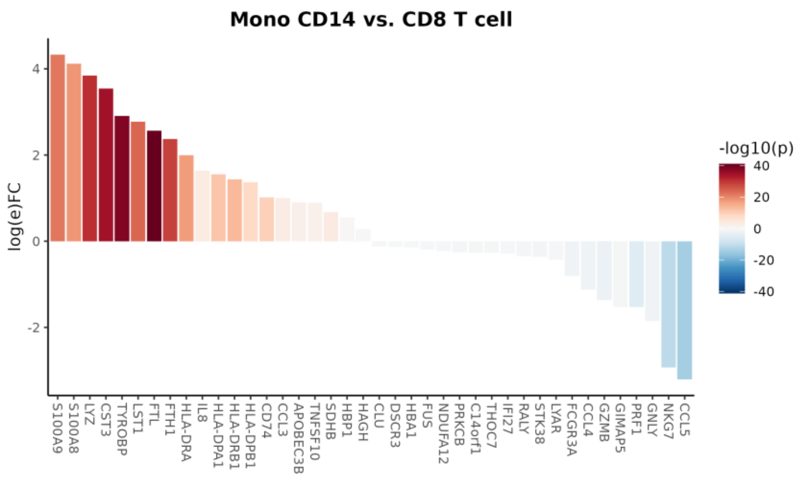

- The hjust and vjust parameters enable fine-tuning of horizontal and vertical label justification, which automatically adapt to the specified angle and flip settings.

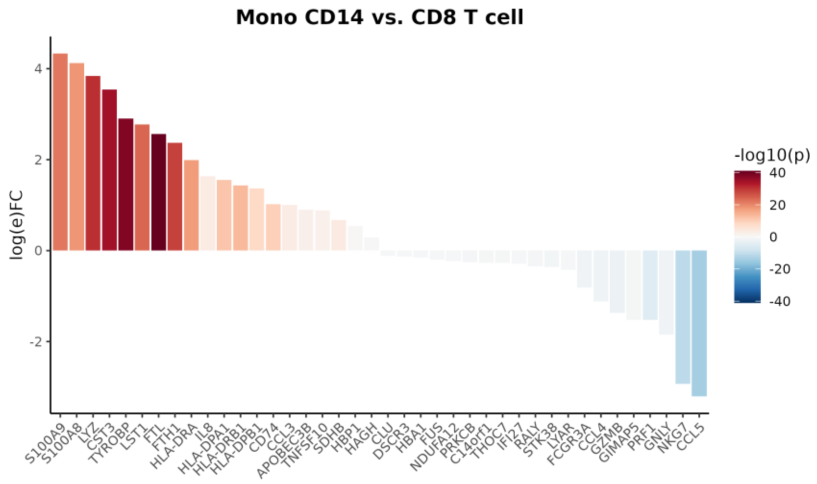

These parameters provide users complete flexibility to adjust gene label directions according to their visualization needs.

6, Regarding the volcano plot, does LogFC mean log2 or log(e)? I noticed that this may not be consistent if you used different tools. For example, some tools like Seurat FindMarkers uses Log2, while NEBULA uses Log(e). Clear labeling on the x-axis and tutorial guidance would help ensure consistency.

We are thankful for this important observation about the inconsistency in log-fold change calculations across different tools. In response to this feedback, we have implemented comprehensive improvements in SeuratExtend v1.2.0 to address this issue directly:

- Clear Documentation and Default Specification: We have updated the documentation for both WaterfallPlot and VolcanoPlot functions to explicitly state that they use natural logarithm (base e) by default for fold change calculations. This aligns with the behavior of NEBULA and provides a mathematical foundation that is natural for statistical analyses.
- User-Selectable Log Base: We've added a new `log.base` parameter to both WaterfallPlot and VolcanoPlot, allowing users to explicitly choose their preferred logarithm base.
- Automatic Axis Labeling: The axis labels on plots now automatically reflect the chosen log base. For example, when using log2, the axis label will explicitly show "log2FC" rather than a generic "logFC" label, eliminating ambiguity.

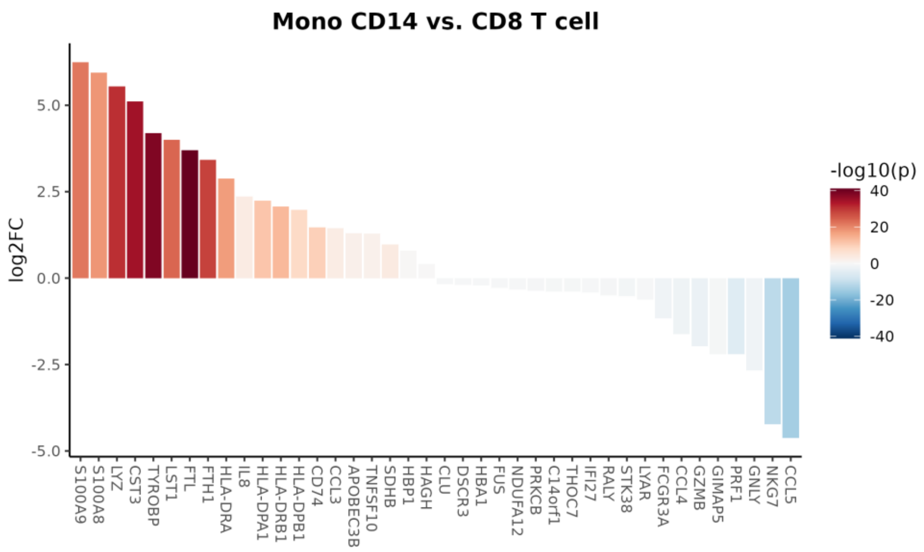

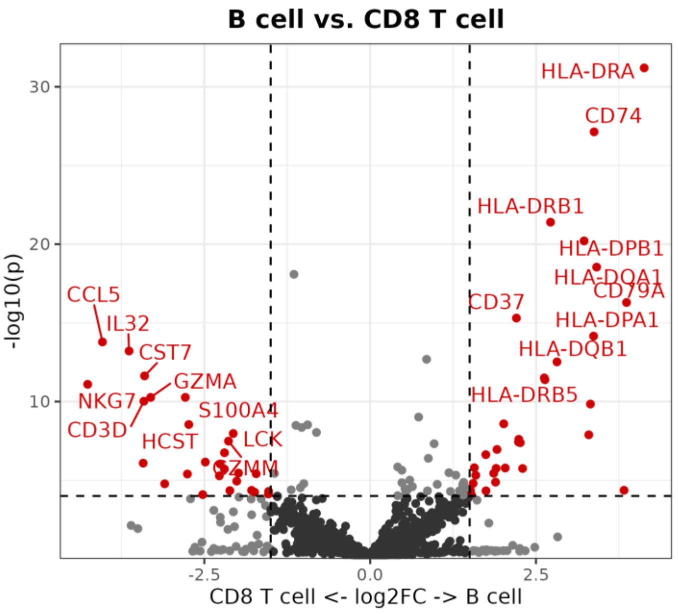

- Formula Transparency: We've included the exact formula used for log fold change calculations in the documentation: `log((mean(x[group1] + pseudocount) / mean(x[group2] + pseudocount)), base)`, making the mathematical basis completely transparent.

These enhancements ensure users have full control over the logarithm base used in their analyses, with clear visual feedback and documentation to prevent confusion. This directly addresses the reviewer's concern while providing additional flexibility for users working across different analytical frameworks.

7, Very nice introduction about the color palettes at the end of the Enhanced Visualization tutorial.

We appreciate the reviewer's kind words regarding our color palette documentation. We've put significant effort into creating accessible and informative color resources that balance aesthetic appeal with scientific clarity.

8, The incorporation of python tools into R is innovative, including scVelo, Palantir. There may be a need to continue incorporating new tools, such as Dynamo, a newer tool I started to use recently. While this is not required for the current revision, it could be a valuable direction for future development.

We are happy about the reviewer's positive feedback on our Python tool integration approach and the valuable suggestion regarding Dynamo. While we haven't included Dynamo in the current release, we agree this would be a valuable addition for future development. Dynamo's advanced capabilities for analyzing cellular dynamics would complement our existing trajectory analysis tools.

In SeuratExtend v1.2.0, our development efforts focused primarily on expanding platform compatibility, particularly adding support for Apple Silicon processors. This required significant technical adjustments to ensure reliable performance across operating systems.

We maintain a development roadmap that prioritizes new tool integration based on analytical value and user feedback, with Dynamo now under consideration for upcoming releases.

Overall, this tool represents a comprehensive extension of Seurat, combining enhanced visualization, pathway enrichment, and trajectory analysis into a single package. I look forward to seeing a revised version of this manuscript.

Reviewer #2: Overall, this is a very nice writeup of a useful package that extends the Seurat package to expand possibilities for single cell analysts in R. I liked the visualization options, the ability to try certain python-based tools easily in R which was not previously easy, and some of the authors' new innovations like their use of pathway enrichment scores in broad ways. Kudos to the authors for releasing a package with really excellent documentation and tutorials!

I think this paper could be made better if the authors stressed with a little more clarity how specifically their work is innovative. The text in the present manuscript is fine but reads like a bit of a grab bag of functionality. For example, from the abstract:

"SeuratExtend offers a user-friendly and intuitive interface for performing a wide range of analyses, including functional enrichment, trajectory inference, gene regulatory network reconstruction, and denoising. The package integrates multiple databases, … and incorporates popular Python tools … [We] showcase its novel applications in pathway-level analysis and cluster annotation. SeuratExtend enhances data visualization …"

How could they be more clear or specific? One example could be by categorizing what SeuratExtend can do that other packages can't. For example, I see innovations in perhaps three general areas:

1. Making single cell analyses easier/faster/prettier (i.e. visualizations, pathway enrichment)

2. Making previously published single cell tools more broadly accessible (e.g. first option to bring certain python tools to R)

3. New innovations (e.g. dimensionality reduction and clustering based on pathway enrichment scores; may not be completely new but I don't recall seeing this elsewhere)

If this was added I feel the paper would more clearly communicate to readers the information necessary for them to choose whether they want to try the package.

We appreciate the reviewer's constructive suggestion about categorizing innovations. We have now restructured the introduction to explicitly categorize SeuratExtend's innovations into three dimensions: (1) making single-cell analyses more efficient and visually compelling; (2) bridging the gap between R and Python ecosystems; and (3) introducing novel methodological approaches, particularly at the pathway level. These innovations are now connected to our core philosophy framework (integration, intuitive design, and visual aesthetics), clarifying the "what"

and "how" relationship. This restructuring directly addresses the reviewer's observation about the manuscript reading as "a grab bag of functionality" by providing a clear conceptual framework for understanding SeuratExtend's value proposition.

I have the following additional significant comments:

* Integration of multiple databases for GSEA — these methods are good, but what about in a few years when those databases have been updated? Do the authors intend to continue updating? Could they provide a function for users to use their own database (e.g. .gaf and .obo files, for example for another model organism)? Similar comment about gene identifer conversion, which may need to be updated every few years.

We thank the reviewer for this important question about database updates for GSEA analysis and gene identifier conversion. In SeuratExtend v1.2.0, we have implemented comprehensive solutions to address both current and future needs:

1. The GO and Reactome databases in SeuratExtend are managed through our companion package SeuratExtendData, which was updated to v0.3.0 in April 2025 with the latest biological information. We've created detailed documentation (README_GO_Data.md and README_Reactome_Data.md) providing step-by-step instructions for users to create custom databases, explaining where to download current files, how to process them, and how to implement them in SeuratExtend. These guides also outline how to support additional species beyond human and mouse, enabling researchers working with non-model organisms to leverage our framework. To facilitate version management, we've added an install_SeuratExtendData() function that allows users to select specific database versions (latest, stable, or specific releases), ensuring compatibility with existing analyses or migration to newer data. We've also included comprehensive information about these features in both the package's FAQ and the GSEA tutorial vignette, ensuring users can easily discover and utilize these capabilities.
2. For gene identifier conversion, all functions in SeuratExtend include a local.mode parameter, which defaults to TRUE for faster performance but can be set to FALSE to use BioMart's online services for the most up-to-date gene identifier mappings. This design ensures that users can always access current gene identifiers even as reference genomes and annotations evolve. As the field advances, we plan to expand our gene symbol conversion capabilities to additional species based on user feedback and research trends.

These features collectively ensure that SeuratExtend remains relevant and accurate as biological databases evolve, while providing users with the flexibility to customize their analytical framework based on specific research needs.

* "While the Python ecosystem has benefited greatly from the comprehensive scverse project [7], which utilizes the universal AnnData format to connect various tools and algorithms, a comparable integrated solution has been lacking in the R community. SeuratExtend addresses this gap by providing a unified framework centered around the Seurat object, effectively becoming the R counterpart to scverse." —> some might argue that SeuratWrappers is this solution. The authors should more clearly and explicitly comment on what SeuratExtend does differently/better than SeuratWrappers.

We acknowledge the reviewer's point about clarifying SeuratExtend's position relative to SeuratWrappers. We have addressed this comprehensively in the revised manuscript through several coordinated updates:

- In the introduction, we now acknowledge SeuratWrappers' valuable contribution while highlighting the remaining integration gap: "In contrast, the R ecosystem, despite having valuable resources like SeuratWrappers with its 17 methods, lacks a similarly comprehensive integration framework."
- We've revised Results section #1 to position SeuratExtend as taking "a similar ecosystem-building approach for the R community, creating a cohesive analytical experience that bridges R and Python environments while maintaining the familiar Seurat workflow."
- Most significantly, we've enhanced the comparison paragraph following Table 1 with specific technical details that distinguish SeuratExtend from SeuratWrappers: "While SeuratWrappers includes 17 methods, only 3 are Python-native (Velocyto, scVelo, PaCMAP), and scVelo's implementation requires users to write Python code for visualization and processing after conversion to h5ad format. In contrast, SeuratExtend enables direct use of Python tools within R, eliminating the need for users to write any Python code."

These revisions collectively provide a clear explanation of SeuratExtend's unique position in the ecosystem and its advantages over existing solutions.

* I'm not particularly convinced by the authors' example studies that used SeuratExtend. For example, they describe Hua-Vella et al. (2022) and Hua et al. (2023). These are very nice

studies and I have no doubt they made use of SeuratExtend in their analyses. But I don't see anything these authors describe those authors doing as being uniquely possible with SeuratExtend. Perhaps SeuratExtend made their analyses easier, or faster. But it would be better if we had some further concrete details. For example, something communicating a message like one of the following: (1) the authors only tested method X on a whim because it was so easy to run in SeuratExtend, and found that it revealed unexpected biology Y; or (2) the authors were able to bring together method X which runs in R and method Y which runs in python and the joint inference — not possible in other packages — revealed key result Z. If the authors of this manuscript can't point to those sorts of examples, then I'm not sure it adds much to include this discussion in the present paper.

We appreciate the reviewer's insightful comment about providing more concrete examples of SeuratExtend's unique contributions to the cited research. We've substantially revised this section to better demonstrate the practical value of our integrated approach.

In the updated manuscript, we now emphasize how SeuratExtend's unified analytical framework facilitated comprehensive multi-angle analyses that would have been challenging with fragmented tools. For the TU-HEV study, we highlight how the ability to seamlessly transition between comparative analyses, trajectory inference, pathway enrichment, and transcription factor networks within a single framework was instrumental in guiding experimental validation efforts. This integration enabled researchers to efficiently explore multiple analytical perspectives, identify the most promising directions for wet lab validation, and ultimately confirm the metaplastic conversion of postcapillary venules to TU-HEVs. Similarly, for the SAID study, we explain how SeuratExtend facilitated rapid cross-disciplinary analysis by combining GSEA with trajectory analysis tools, leading to the discovery of an unexpected therapeutic mechanism involving macrophage differentiation pathways rather than simple cytokine suppression.

The revised section better illustrates how SeuratExtend's integrated workflow supports comprehensive data exploration and accelerates the discovery process by removing technical barriers between complementary analytical methods. We believe this revision addresses the reviewer's concern while demonstrating the practical value of SeuratExtend in facilitating biological insights that might otherwise require significantly more effort to uncover.

* I really liked the section "Novel Applications of SeuratExtend in Pathway-Level Analysis and Cluster Annotation", especially "Exploring and Analyzing Single-Cell Data at the Pathway

Level". I thought these applications could perhaps be stressed a bit more strongly or made more prominent earlier in the paper.

We welcome the reviewer's positive feedback on our "Novel Applications of SeuratExtend in Pathway-Level Analysis and Cluster Annotation" section. We agree that this represents a key innovation of SeuratExtend that merits greater prominence in the manuscript.

To address this valuable suggestion, we have made three strategic changes:

- We explicitly highlighted pathway-level analysis in the introduction as one of SeuratExtend's key innovations: "it pioneers novel methodological approaches, particularly pathway-level analysis that provides new perspectives on cellular heterogeneity."
- We expanded the final paragraph of Section #1 ("SeuratExtend: A Comprehensive R Ecosystem for Single-Cell Analysis") to provide more detailed emphasis on our pathway-level analysis approach, explaining how it "transforms how researchers can explore and interpret cellular heterogeneity beyond individual gene expression patterns."
- We repositioned the "Novel Applications" section to appear before the "Educational Impact" section, giving it greater prominence in the paper's overall flow while maintaining the logical progression of technical content.

These changes enhance the visibility of this innovative approach throughout the manuscript while preserving the paper's structural integrity and the necessary technical dependencies between sections.

* Figures 2 and 3 are showing example plots from which we don't actually need to infer any important biology. I thought these figures could be combined and each individual plot type only shown once. (This is for clarity and I don't see anything incorrect about the authors' current plots.

We appreciate the reviewer's insightful suggestion about potentially combining Figures 2 and 3. After careful consideration, we have decided to maintain the current organization of these figures for the following reasons:

1. These figures represent distinct functional modules of SeuratExtend: Figure 2 demonstrates GSEA-related visualizations while Figure 3 showcases the integration of Python tools for

trajectory analysis, gene regulatory networks, and denoising. Each panel within these figures serves a unique purpose in illustrating different capabilities:

- Figure 2A (heatmap) shows how pathway enrichment scores can be visualized across multiple cell types
- Figure 2B (violin plots) demonstrates statistical comparisons between groups
- Figure 2C (enrichment plot) illustrates a different approach to pathway visualization
- Figure 3A-D demonstrate different trajectory analysis tools (scVelo vectors, Palantir diffusion maps, pseudotime plots, and fate marker heatmaps)
- Figure 3E-G show the progression from gene expression to denoised expression to regulatory networks

2. While some visualization types (like UMAPs) appear in both figures, they serve different analytical purposes - in Figure 3A they display velocity vectors from scVelo, while in Figure 3E they demonstrate the contrast between raw, denoised (MAGIC), and transcription factor activity (SCENIC) data. These distinct applications illustrate the package's versatility across different analytical contexts.
3. We considered combining them, but technical limitations make this challenging: the heatmap in Figure 2A and the regulatory networks in Figure 3G, for example, need adequate space to maintain their readability and information content. Combining these diverse visualization types while preserving their interpretability would likely result in an overly complex composite figure that might diminish clarity.

We believe the current organization provides the optimal balance between comprehensiveness and clarity, allowing readers to better understand the distinct capabilities of SeuratExtend's analytical modules. We greatly appreciate the reviewer's suggestion aimed at improving manuscript clarity.

* There may be some issues with dependencies for some users. For example, it prompted me to install viridis and loomR as I went through the Quickstart. I ended up encountering an error `there is no package called 'loomR'` while trying. I had to manually install with `remotes::install_github(repo = "mojaveazure/loomR")`. Maybe provide an explicit dependencies list/list of recommended packages to install?

We appreciate the reviewer's feedback regarding dependency management in SeuratExtend. The error message "there is no package called 'loomR'" that the reviewer encountered is related to our internal function named import(), which is responsible for handling package dependencies in

SeuratExtend. This function automatically checks for required packages when users call specific functions, and if a package is missing, it provides installation instructions tailored to the package's source (CRAN, Bioconductor, or GitHub) and handles the installation process with user permission. The misleading error message was caused by how this function processes warnings from the require() function in R.

We have made improvements to address this issue:

1. Fixed warning handling: We have updated our import() function to suppress the confusing "there is no package called..." warnings and replace them with clear guidance messages. The function now verifies successful installation before proceeding and provides specific error messages when installation fails.
2. Comprehensive dependency management: SeuratExtend already provides a detailed list of dependencies in the DESCRIPTION file of the package, separated into different categories:
   - Imports: Seurat, dplyr, remotes, rlist, mosaic, ggplot2, reshape2, purrr, magrittr, tidyr, scales, ggpubr, BiocManager, reticulate, glue, hdf5r
   - Suggests: rgl, GSVA, GSVAdata, slingshot, AUCell, biomaRt, ontologyIndex, viridis, doMC, doRNG, doParallel, foreach, mgsa, GSEABase, egg, loomR, mgsa, knitr, rmarkdown, RColorBrewer
   - Depends: R (>= 3.6), SeuratExtendData, SeuratObject

This separation allows our package to use a just-in-time approach to dependency management - core packages in "Imports" are installed automatically with SeuratExtend, while specialized packages in "Suggests" (like loomR) are only installed when a user invokes a function that requires them. All core packages in "Imports" are sourced from CRAN, ensuring reliability and stability for SeuratExtend's essential functionality. This approach prevents users from installing unnecessary dependencies for functions they may never use.

* I had an error the first time calling Palantir.RunDM(). I hadn't created a seuratextend environment. I found that I could do this manually using `create_condaenv_seuratextend()`, but that this wasn't supported for Apple Silicon chips. I would suggest that the authors do try to find a way to get this working on newer Apple chips, because Mac machines are very common among bioinformaticians in my experience.

We appreciate the reviewer's feedback regarding Apple Silicon support. We are pleased to announce that as of SeuratExtend v1.2.0, we have added support for Apple Silicon

(M1/M2/M3/M4) Macs, though with some limitations that we've addressed with specific workarounds.

During our comprehensive testing on M4 chips, we identified a persistent memory management issue between R and Python that is specific to Apple Silicon architecture. Specifically, if any R objects (such as Seurat objects) are loaded in the R session before initializing Python functions, Python operations like PCA on AnnData objects would consistently cause the R session to crash. This appears to be related to how memory is allocated and shared between R and Python environments on the ARM64 architecture.

To address this issue, we've implemented a solution through our new `activate_python()` function, which must be called at the beginning of a fresh R session before loading any R objects. This function properly initializes the Python environment with the necessary memory allocations, preventing the crashes that would otherwise occur during operations like `Palantir.RunDM()`.

While this workaround requires a specific workflow sequence on Apple Silicon Macs (initialize Python first, then load R objects), it provides a reliable solution that enables all Python-dependent functions to work properly. This workflow is fully documented in our package vignettes and FAQ, with detailed step-by-step guidance for macOS users.

* While the writing is largely quite clear, I found it to be a bit voluminous. If the authors are able to cut down on text length that may help in emphasizing the key points that make their package valuable to users.

We acknowledge your observation about the manuscript being voluminous. We have made substantial reductions to improve clarity and emphasis on key points:

1. Introduction - Condensed the discussion of challenges in scRNA-seq analysis from two full paragraphs to one focused paragraph that retains the most relevant examples; Streamlined the introduction of our core design principles while maintaining their conceptual clarity.
2. Results section - Condensed the "Evolving Applications of SeuratExtend" section by approximately 40%, focusing on specific examples of how the integrated framework facilitated biological discoveries rather than detailed study descriptions; eliminated redundant explanations of analytical methods that were already covered in earlier sections. Streamlined the "Seamless Integration of Python Tools within an R Environment" section by approximately 30%, reducing technical implementation details that overlapped with the Methods section while preserving the key innovations and benefits. Specifically, we condensed paragraphs

describing data format conversion and environment setup into a more concise explanation that focuses on user benefits rather than implementation mechanics.

These reductions improve the manuscript's flow while emphasizing the key innovations and practical value of SeuratExtend.

I had these minor comments:

* "Moreover, mainstream scRNA-seq analysis tools are primarily developed for either the R or Python platforms, with additional options like Nextflow and Snakemake" — I suggest revising this sentence. The tools are developed in R or python languages, which I would not call platforms. I would reword that Nextflow and Snakemake are workflow management systems that provide additional options for pipeline automation

We thank the reviewer for this terminological correction. We have revised this to "R or Python languages" and clarified that Nextflow and Snakemake are workflow management systems rather than programming languages. The sentence now reads: "Moreover, mainstream scRNA-seq analysis tools are primarily developed in either the R or Python languages, with additional options like Nextflow and Snakemake as workflow management systems."

* "the R ecosystem surrounding Seurat appears relatively limited" — I'm not sure I would agree with this. I counted wrappers for 17 methods currently. Yes it is true that there are more packages in scverse. However, I suggest moderating your claims about Seurat being limited.

We thank the reviewer for highlighting the substantial contributions of SeuratWrappers with its 17 methods and the broader value of tools in the R ecosystem. We have moderated our claims to acknowledge these resources while maintaining our point about integration frameworks. The revised sentence now states: "In contrast, the R ecosystem, despite having valuable resources like SeuratWrappers with its 17 methods, lacks a similarly comprehensive integration framework."

* Suggest removing snakemake from Table 1 — it is really different from the other tools listed there

We appreciate this insightful observation regarding Snakemake's placement in Table 1. We agree that Snakemake, as a workflow management system rather than a direct single-cell analysis tool, is conceptually different from the other software packages in the comparison. Following your suggestion, we have removed Snakemake from Table 1 to maintain consistency in the comparison between software packages specifically designed for single-cell analysis. We have

also adjusted the corresponding text in the manuscript to reflect this change, ensuring that our comparisons focus on tools with similar primary functions while still acknowledging workflow management systems in our broader discussion of the ecosystem.
