# Supplementary material for: SeuratExtend: streamlining single-cell RNA-seq analysis through an integrated and intuitive framework
Source: Gigascience. 2025 Jul 8;14:giaf076. doi: 10.1093/gigascience/giaf076 (PMC12236070; doi:10.1093/gigascience/giaf076)
Supplement: giaf076_Authors_Response_To_Reviewer_Comments_Original_Submission [file giaf076_authors_response_to_reviewer_comments_original_submission.pdf]

Author's Response To Reviewer Comments

Reviewer reports:
Reviewer #1: This manuscript introduces an extended version of the widely-used Seurat package, named SeuratExtend. Specifically, Hua et al. developed an integrated an intuitive framework to streamline scRNA-seq data analysis, such as trajectory analysis, GRN construction, and functional enrichment analysis. The package also features direct integration with other popular tools, including Seurat, scVelo, etc. Notably, the software has been demonstrated through training programs, with over 100 stars on GitHub, which is impressive. I have tested the package, including installation and some basic functions. Moreover, the GitHub webpage is well-documented, featuring multiple use cases tailored for beginners. The overall user experience exceeded my expectations, though I have a few minor comments for improvement:
1, The DimPlot2 function is very useful, and easy to customize the colors. However, the default color scheme seems to be too dark. Considering a more distinguishable and visually appealing color palette might be a solution.

RESPONSE:
We appreciate the reviewer's valuable feedback regarding the DimPlot2 function's default color scheme. This observation aligns with feedback from multiple users who found the default "dark" palette too intense for certain visualization contexts.
In response to this feedback, we have implemented several significant improvements in SeuratExtend v1.2.0:
· We have changed the default color scheme for discrete variables from "default" (dark) to "light" - a more visually comfortable palette with increased brightness and reduced saturation. This change makes plots more readable and visually appealing, particularly when labels are displayed.
· We have introduced a new "bright" color scheme that offers higher contrast colors for visualizations requiring more distinct separation between groups. This option is particularly useful for presentations or when subtle color differences need to be emphasized.
· We've expanded the maximum number of colors supported by our color palettes from 50 to 80, accommodating datasets with larger numbers of clusters or categories.
These enhancements provide users with greater flexibility in choosing color schemes that best suit their specific visualization needs while addressing the reviewer's concerns about distinguishability and visual appeal. The changes have been fully documented in our package vignettes with illustrative examples.

2, How to control the angles of cell type labels when using VlnPlot2? The 'Split visualization' has all the labels in a horizontal direction, leading to overlapping in some cases, while 'Subset Analysis' plots have labels in 45 degree, which is much better to read. However, I didn't see a parameter to control this. Does VlnPlot2 handle this automatically?

RESPONSE:
We agree with the reviewer's observation regarding label angles in VlnPlot2 visualizations. In response to this feedback, we have implemented a significant enhancement to the function in SeuratExtend v1.2.0. Previously, the VlnPlot2 function automatically determined label angles based on label length (using 0 degrees for short labels and 45 degrees for longer ones), but this automatic adjustment was only available for non-split visualizations. The split visualization displayed labels horizontally, which could lead to overlapping text with longer labels as the reviewer correctly pointed out.
We have now added three new parameters to VlnPlot2:
· angle: Controls the rotation angle of labels
· hjust: Controls horizontal justification of labels
· vjust: Controls vertical justification of labels
All three parameters default to NULL, in which case the function automatically determines appropriate values based on label length and plot type:
· For non-split visualizations: 0 degrees for short labels (≤2 characters), 45 degrees for longer labels
· For split visualizations: 0 degrees for short labels, -90 degrees (vertical) for longer labels
Users can also manually override these settings to achieve their preferred visualization style. The automatically determined hjust and vjust values ensure optimal text alignment based on the rotation

angle.
This enhancement addresses the reviewer's concern while maintaining backward compatibility and providing additional flexibility for customization.

3, It's a very nice feature to have the 'Statistical Analysis' function to label significant groups. However, in single cell analysis, the p values are easy to be inflated due to the large number of cells. While the example pmbc data is relatively small, larger datasets might yield significant p values without obvious differences in the violin plots. It would be beneficial to mention this in the documentation, and provide some guidance so the results won't be misleading.

RESPONSE:
We appreciate the reviewer's insightful point regarding p-value inflation in single-cell analysis with the 'Statistical Analysis' function. This is indeed an important consideration that we've now addressed in SeuratExtend v1.2.0.
We have added explicit warnings about this issue in both the function documentation and the package vignette. Specifically, we now caution users that p-values can be artificially inflated in large single-cell datasets due to the high number of cells, potentially resulting in statistically significant differences (small p-values) even when the biological effect size is minimal.
The documentation now emphasizes that:
· Users should be cautious with statistical interpretations, especially when visual differences are subtle
· We recommend examining log fold changes (logFC) between groups to better assess the magnitude of biological differences
· Additionally, the percentage of cells expressing a marker (similar to pct.1 and pct.2 values in Seurat's FindMarkers) is an important metric to consider - if the difference in percentage between groups is minimal, the expression difference may be biologically negligible despite a significant p-value
· Parameters like min.pct in differential expression analysis can help filter out features with low expression prevalence
· For a comprehensive visualization of differences between two groups, our WaterfallPlot() function can provide logFC values along with statistical significance
· The function uses the Holm method (p.adjust.method = "holm") by default to adjust p-values for multiple comparisons
This warning appears in both the R documentation accessible via ?VlnPlot2 and in the "Enhanced Visualization" vignette, ensuring users are properly informed about this statistical consideration when interpreting their results.
We believe these additions directly address the reviewer's concern while providing practical guidance to users when applying statistical analysis to single-cell data.

4, The ClusterDistrBar is another valuable function. Based on my experience with similar analyses, I suggest incorporating features to identify robust changes in cell type composition. For instance, tools like sccomp can help determine changes in cell population composition.

RESPONSE:
We value the suggestion regarding enhancement of the ClusterDistrBar function to identify robust changes in cell type composition. We agree that this is an important aspect of single-cell analysis, especially when comparing multiple conditions or samples.
We have implemented a new function in SeuratExtend v1.2.0 called ClusterDistrPlot, which extends the functionality of ClusterDistrBar. This new function allows users to compare cluster distributions between experimental conditions using boxplots instead of stacked bars, and perform statistical testing (Wilcoxon rank-sum test or t-test) to identify significant differences in cell type proportions between conditions.

The ClusterDistrPlot function maintains backward compatibility with ClusterDistrBar while adding these new features specifically designed for compositional analysis. It applies various visualization parameters inherited from VlnPlot2 for customized presentation. Our implementation was inspired by specialized tools like sccomp, maintaining the same intuitive interface and visualization style consistent with other SeuratExtend functions.

5, I wonder if the gene label directions can be changed easily for WaterfallPlot?

RESPONSE:
We welcome the reviewer's inquiry about changing gene label directions in the WaterfallPlot function. WaterfallPlot has included comprehensive label orientation controls through several parameters:
· The flip parameter controls the overall plot orientation (horizontal vs. vertical bars), with flip=TRUE displaying genes on the y-axis and flip=FALSE displaying genes on the x-axis.
· The angle parameter allows precise control over gene label rotation angles, defaulting to -90 degrees for vertical plots and 0 degrees for horizontal plots, but users can specify any custom angle.

· The hjust and vjust parameters enable fine-tuning of horizontal and vertical label justification, which automatically adapt to the specified angle and flip settings.

These parameters provide users complete flexibility to adjust gene label directions according to their visualization needs.

6, Regarding the volcano plot, does LogFC mean log2 or log(e)? I noticed that this may not be consistent if you used different tools. For example, some tools like Seurat FindMarkers uses Log2, while NEBULA uses Log(e). Clear labeling on the x-axis and tutorial guidance would help ensure consistency.

RESPONSE:
We are thankful for this important observation about the inconsistency in log-fold change calculations across different tools. In response to this feedback, we have implemented comprehensive improvements in SeuratExtend v1.2.0 to address this issue directly:
· Clear Documentation and Default Specification: We have updated the documentation for both WaterfallPlot and VolcanoPlot functions to explicitly state that they use natural logarithm (base e) by default for fold change calculations. This aligns with the behavior of NEBULA and provides a mathematical foundation that is natural for statistical analyses.
· User-Selectable Log Base: We've added a new `log.base` parameter to both WaterfallPlot and VolcanoPlot, allowing users to explicitly choose their preferred logarithm base.
· Automatic Axis Labeling: The axis labels on plots now automatically reflect the chosen log base. For example, when using log2, the axis label will explicitly show "log2FC" rather than a generic "logFC" label, eliminating ambiguity.
· Formula Transparency: We've included the exact formula used for log fold change calculations in the documentation: `log((mean(x[group1] + pseudocount) / mean(x[group2] + pseudocount)), base)`, making the mathematical basis completely transparent.
These enhancements ensure users have full control over the logarithm base used in their analyses, with clear visual feedback and documentation to prevent confusion. This directly addresses the reviewer's concern while providing additional flexibility for users working across different analytical frameworks.

7, Very nice introduction about the color palettes at the end of the Enhanced Visualization tutorial.

RESPONSE:
We appreciate the reviewer's kind words regarding our color palette documentation. We've put significant effort into creating accessible and informative color resources that balance aesthetic appeal with scientific clarity.

8, The incorporation of python tools into R is innovative, including scVelo, Palantir. There may be a need to continue incorporating new tools, such as Dynamo, a newer tool I started to use recently. While this is not required for the current revision, it could be a valuable direction for future development.

RESPONSE:
We are happy about the reviewer's positive feedback on our Python tool integration approach and the valuable suggestion regarding Dynamo. While we haven't included Dynamo in the current release, we agree this would be a valuable addition for future development. Dynamo's advanced capabilities for analyzing cellular dynamics would complement our existing trajectory analysis tools.
In SeuratExtend v1.2.0, our development efforts focused primarily on expanding platform compatibility, particularly adding support for Apple Silicon processors. This required significant technical adjustments to

ensure reliable performance across operating systems.
We maintain a development roadmap that prioritizes new tool integration based on analytical value and user feedback, with Dynamo now under consideration for upcoming releases.

Overall, this tool represents a comprehensive extension of Seurat, combining enhanced visualization, pathway enrichment, and trajectory analysis into a single package. I look forward to seeing a revised version of this manuscript.

Reviewer #2: Overall, this is a very nice writeup of a useful package that extends the Seurat package to expand possibilities for single cell analysts in R. I liked the visualization options, the ability to try certain python-based tools easily in R which was not previously easy, and some of the authors' new innovations like their use of pathway enrichment scores in broad ways. Kudos to the authors for releasing a package with really excellent documentation and tutorials!
I think this paper could be made better if the authors stressed with a little more clarity how specifically their work is innovative. The text in the present manuscript is fine but reads like a bit of a grab bag of functionality. For example, from the abstract:
"SeuratExtend offers a user-friendly and intuitive interface for performing a wide range of analyses, including functional enrichment, trajectory inference, gene regulatory network reconstruction, and denoising. The package integrates multiple databases, … and incorporates popular Python tools … [We] showcase its novel applications in pathway-level analysis and cluster annotation. SeuratExtend enhances data visualization …"
How could they be more clear or specific? One example could be by categorizing what SeuratExtend can do that other packages can't. For example, I see innovations in perhaps three general areas:
1. Making single cell analyses easier/faster/prettier (i.e. visualizations, pathway enrichment)
2. Making previously published single cell tools more broadly accessible (e.g. first option to bring certain python tools to R)
3. New innovations (e.g. dimensionality reduction and clustering based on pathway enrichment scores; may not be completely new but I don't recall seeing this elsewhere)
If this was added I feel the paper would more clearly communicate to readers the information necessary for them to choose whether they want to try the package.

RESPONSE:
We appreciate the reviewer's constructive suggestion about categorizing innovations. We have now restructured the introduction to explicitly categorize SeuratExtend's innovations into three dimensions: (1) making single-cell analyses more efficient and visually compelling; (2) bridging the gap between R and Python ecosystems; and (3) introducing novel methodological approaches, particularly at the pathway level. These innovations are now connected to our core philosophy framework (integration, intuitive design, and visual aesthetics), clarifying the "what" and "how" relationship. This restructuring directly addresses the reviewer's observation about the manuscript reading as "a grab bag of functionality" by providing a clear conceptual framework for understanding SeuratExtend's value proposition.

I have the following additional significant comments:
* Integration of multiple databases for GSEA — these methods are good, but what about in a few years when those databases have been updated? Do the authors intend to continue updating? Could they provide a function for users to use their own database (e.g. .gaf and .obo files, for example for another model organism)? Similar comment about gene identifer conversion, which may need to be updated every few years.

RESPONSE:
We thank the reviewer for this important question about database updates for GSEA analysis and gene identifier conversion. In SeuratExtend v1.2.0, we have implemented comprehensive solutions to address both current and future needs:
1. The GO and Reactome databases in SeuratExtend are managed through our companion package SeuratExtendData, which was updated to v0.3.0 in April 2025 with the latest biological information. We've created detailed documentation (README_GO_Data.md and README_Reactome_Data.md) providing step-by-step instructions for users to create custom databases, explaining where to download current files, how to process them, and how to implement them in SeuratExtend. These guides also outline how to support

additional species beyond human and mouse, enabling researchers working with non-model organisms to leverage our framework. To facilitate version management, we've added an install_SeuratExtendData() function that allows users to select specific database versions (latest, stable, or specific releases), ensuring compatibility with existing analyses or migration to newer data. We've also included comprehensive information about these features in both the package's FAQ and the GSEA tutorial vignette, ensuring users can easily discover and utilize these capabilities.
2. For gene identifier conversion, all functions in SeuratExtend include a local.mode parameter, which defaults to TRUE for faster performance but can be set to FALSE to use BioMart's online services for the most up-to-date gene identifier mappings. This design ensures that users can always access current gene identifiers even as reference genomes and annotations evolve. As the field advances, we plan to expand our gene symbol conversion capabilities to additional species based on user feedback and research trends.
These features collectively ensure that SeuratExtend remains relevant and accurate as biological databases evolve, while providing users with the flexibility to customize their analytical framework based on specific research needs.

* "While the Python ecosystem has benefited greatly from the comprehensive scverse project [7], which utilizes the universal AnnData format to connect various tools and algorithms, a comparable integrated solution has been lacking in the R community. SeuratExtend addresses this gap by providing a unified framework centered around the Seurat object, effectively becoming the R counterpart to scverse." —> some might argue that SeuratWrappers is this solution. The authors should more clearly and explicitly comment on what SeuratExtend does differently/better than SeuratWrappers.

RESPONSE:
We acknowledge the reviewer's point about clarifying SeuratExtend's position relative to SeuratWrappers. We have addressed this comprehensively in the revised manuscript through several coordinated updates:
· In the introduction, we now acknowledge SeuratWrappers' valuable contribution while highlighting the remaining integration gap: "In contrast, the R ecosystem, despite having valuable resources like SeuratWrappers with its 17 methods, lacks a similarly comprehensive integration framework."
· We've revised Results section #1 to position SeuratExtend as taking "a similar ecosystem-building approach for the R community, creating a cohesive analytical experience that bridges R and Python environments while maintaining the familiar Seurat workflow."
· Most significantly, we've enhanced the comparison paragraph following Table 1 with specific technical details that distinguish SeuratExtend from SeuratWrappers: "While SeuratWrappers includes 17 methods, only 3 are Python-native (Velocyto, scVelo, PaCMAP), and scVelo's implementation requires users to write Python code for visualization and processing after conversion to h5ad format. In contrast, SeuratExtend enables direct use of Python tools within R, eliminating the need for users to write any Python code."
These revisions collectively provide a clear explanation of SeuratExtend's unique position in the ecosystem and its advantages over existing solutions.

* I'm not particularly convinced by the authors' example studies that used SeuratExtend. For example, they describe Hua-Vella et al. (2022) and Hua et al. (2023). These are very nice studies and I have no doubt they made use of SeuratExtend in their analyses. But I don't see anything these authors describe those authors doing as being uniquely possible with SeuratExtend. Perhaps SeuratExtend made their analyses easier, or faster. But it would be better if we had some further concrete details. For example, something communicating a message like one of the following: (1) the authors only tested method X on a whim because it was so easy to run in SeuratExtend, and found that it revealed unexpected biology Y; or (2) the authors were able to bring together method X which runs in R and method Y which runs in python and the joint inference — not possible in other packages — revealed key result Z. If the authors of this manuscript can't point to those sorts of examples, then I'm not sure it adds much to include this discussion in the present paper.

RESPONSE:
We appreciate the reviewer's insightful comment about providing more concrete examples of SeuratExtend's unique contributions to the cited research. We've substantially revised this section to better demonstrate the practical value of our integrated approach.
In the updated manuscript, we now emphasize how SeuratExtend's unified analytical framework facilitated comprehensive multi-angle analyses that would have been challenging with fragmented tools. For the TU-

HEV study, we highlight how the ability to seamlessly transition between comparative analyses, trajectory inference, pathway enrichment, and transcription factor networks within a single framework was instrumental in guiding experimental validation efforts. This integration enabled researchers to efficiently explore multiple analytical perspectives, identify the most promising directions for wet lab validation, and ultimately confirm the metaplastic conversion of postcapillary venules to TU-HEVs. Similarly, for the SAID study, we explain how SeuratExtend facilitated rapid cross-disciplinary analysis by combining GSEA with trajectory analysis tools, leading to the discovery of an unexpected therapeutic mechanism involving macrophage differentiation pathways rather than simple cytokine suppression.
The revised section better illustrates how SeuratExtend's integrated workflow supports comprehensive data exploration and accelerates the discovery process by removing technical barriers between complementary analytical methods. We believe this revision addresses the reviewer's concern while demonstrating the practical value of SeuratExtend in facilitating biological insights that might otherwise require significantly more effort to uncover.

* I really liked the section "Novel Applications of SeuratExtend in Pathway-Level Analysis and Cluster Annotation", especially "Exploring and Analyzing Single-Cell Data at the Pathway Level". I thought these applications could perhaps be stressed a bit more strongly or made more prominent earlier in the paper.

RESPONSE:
We welcome the reviewer's positive feedback on our "Novel Applications of SeuratExtend in Pathway-Level Analysis and Cluster Annotation" section. We agree that this represents a key innovation of SeuratExtend that merits greater prominence in the manuscript.
To address this valuable suggestion, we have made three strategic changes:
· We explicitly highlighted pathway-level analysis in the introduction as one of SeuratExtend's key innovations: "it pioneers novel methodological approaches, particularly pathway-level analysis that provides new perspectives on cellular heterogeneity."
· We expanded the final paragraph of Section #1 ("SeuratExtend: A Comprehensive R Ecosystem for Single-Cell Analysis") to provide more detailed emphasis on our pathway-level analysis approach, explaining how it "transforms how researchers can explore and interpret cellular heterogeneity beyond individual gene expression patterns."
· We repositioned the "Novel Applications" section to appear before the "Educational Impact" section, giving it greater prominence in the paper's overall flow while maintaining the logical progression of technical content.
These changes enhance the visibility of this innovative approach throughout the manuscript while preserving the paper's structural integrity and the necessary technical dependencies between sections.

* Figures 2 and 3 are showing example plots from which we don't actually need to infer any important biology. I thought these figures could be combined and each individual plot type only shown once. (This is for clarity and I don't see anything incorrect about the authors' current plots.

RESPONSE:
We appreciate the reviewer's insightful suggestion about potentially combining Figures 2 and 3. After careful consideration, we have decided to maintain the current organization of these figures for the following reasons:
1. These figures represent distinct functional modules of SeuratExtend: Figure 2 demonstrates GSEA-related visualizations while Figure 3 showcases the integration of Python tools for trajectory analysis, gene regulatory networks, and denoising. Each panel within these figures serves a unique purpose in illustrating different capabilities:
· Figure 2A (heatmap) shows how pathway enrichment scores can be visualized across multiple cell types
· Figure 2B (violin plots) demonstrates statistical comparisons between groups
· Figure 2C (enrichment plot) illustrates a different approach to pathway visualization
· Figure 3A-D demonstrate different trajectory analysis tools (scVelo vectors, Palantir diffusion maps, pseudotime plots, and fate marker heatmaps)
· Figure 3E-G show the progression from gene expression to denoised expression to regulatory networks
2. While some visualization types (like UMAPs) appear in both figures, they serve different analytical purposes - in Figure 3A they display velocity vectors from scVelo, while in Figure 3E they demonstrate the contrast between raw, denoised (MAGIC), and transcription factor activity (SCENIC) data. These distinct

applications illustrate the package's versatility across different analytical contexts.
3. We considered combining them, but technical limitations make this challenging: the heatmap in Figure 2A and the regulatory networks in Figure 3G, for example, need adequate space to maintain their readability and information content. Combining these diverse visualization types while preserving their interpretability would likely result in an overly complex composite figure that might diminish clarity.
We believe the current organization provides the optimal balance between comprehensiveness and clarity, allowing readers to better understand the distinct capabilities of SeuratExtend's analytical modules. We greatly appreciate the reviewer's suggestion aimed at improving manuscript clarity.

* There may be some issues with dependencies for some users. For example, it prompted me to install viridis and loomR as I went through the Quickstart. I ended up encountering an error `there is no package called 'loomR'` while trying. I had to manually install with `remotes::install_github(repo = "mojaveazure/loomR")`. Maybe provide an explicit dependencies list/list of recommended packages to install?

RESPONSE:
We appreciate the reviewer's feedback regarding dependency management in SeuratExtend. The error message "there is no package called 'loomR'" that the reviewer encountered is related to our internal function named import(), which is responsible for handling package dependencies in SeuratExtend. This function automatically checks for required packages when users call specific functions, and if a package is missing, it provides installation instructions tailored to the package's source (CRAN, Bioconductor, or GitHub) and handles the installation process with user permission. The misleading error message was caused by how this function processes warnings from the require() function in R.
We have made improvements to address this issue:
1. Fixed warning handling: We have updated our import() function to suppress the confusing "there is no package called..." warnings and replace them with clear guidance messages. The function now verifies successful installation before proceeding and provides specific error messages when installation fails.
2. Comprehensive dependency management: SeuratExtend already provides a detailed list of dependencies in the DESCRIPTION file of the package, separated into different categories:
· Imports: Seurat, dplyr, remotes, rlist, mosaic, ggplot2, reshape2, purrr, magrittr, tidyr, scales, ggpubr, BiocManager, reticulate, glue, hdf5r
· Suggests: rgl, GSVA, GSVAdata, slingshot, AUCell, biomaRt, ontologyIndex, viridis, doMC, doRNG, doParallel, foreach, mgsa, GSEABase, egg, loomR, mgsa, knitr, rmarkdown, RColorBrewer
· Depends: R (>= 3.6), SeuratExtendData, SeuratObject
This separation allows our package to use a just-in-time approach to dependency management - core packages in "Imports" are installed automatically with SeuratExtend, while specialized packages in "Suggests" (like loomR) are only installed when a user invokes a function that requires them. All core packages in "Imports" are sourced from CRAN, ensuring reliability and stability for SeuratExtend's essential functionality. This approach prevents users from installing unnecessary dependencies for functions they may never use.

* I had an error the first time calling Palantir.RunDM(). I hadn't created a seuratextend environment. I found that I could do this manually using `create_condaenv_seuratextend()`, but that this wasn't supported for Apple Silicon chips. I would suggest that the authors do try to find a way to get this working on newer Apple chips, because Mac machines are very common among bioinformaticians in my experience.

RESPONSE:
We appreciate the reviewer's feedback regarding Apple Silicon support. We are pleased to announce that as of SeuratExtend v1.2.0, we have added support for Apple Silicon (M1/M2/M3/M4) Macs, though with some limitations that we've addressed with specific workarounds.
During our comprehensive testing on M4 chips, we identified a persistent memory management issue between R and Python that is specific to Apple Silicon architecture. Specifically, if any R objects (such as Seurat objects) are loaded in the R session before initializing Python functions, Python operations like PCA on AnnData objects would consistently cause the R session to crash. This appears to be related to how memory is allocated and shared between R and Python environments on the ARM64 architecture.
To address this issue, we've implemented a solution through our new `activate_python()` function, which must be called at the beginning of a fresh R session before loading any R objects. This function properly

initializes the Python environment with the necessary memory allocations, preventing the crashes that would otherwise occur during operations like `Palantir.RunDM()`.
While this workaround requires a specific workflow sequence on Apple Silicon Macs (initialize Python first, then load R objects), it provides a reliable solution that enables all Python-dependent functions to work properly. This workflow is fully documented in our package vignettes and FAQ, with detailed step-by-step guidance for macOS users.

* While the writing is largely quite clear, I found it to be a bit voluminous. If the authors are able to cut down on text length that may help in emphasizing the key points that make their package valuable to users.

RESPONSE:
We acknowledge your observation about the manuscript being voluminous. We have made substantial reductions to improve clarity and emphasis on key points:
1. Introduction - Condensed the discussion of challenges in scRNA-seq analysis from two full paragraphs to one focused paragraph that retains the most relevant examples; Streamlined the introduction of our core design principles while maintaining their conceptual clarity.
2. Results section - Condensed the "Evolving Applications of SeuratExtend" section by approximately 40%, focusing on specific examples of how the integrated framework facilitated biological discoveries rather than detailed study descriptions; eliminated redundant explanations of analytical methods that were already covered in earlier sections. Streamlined the "Seamless Integration of Python Tools within an R Environment" section by approximately 30%, reducing technical implementation details that overlapped with the Methods section while preserving the key innovations and benefits. Specifically, we condensed paragraphs describing data format conversion and environment setup into a more concise explanation that focuses on user benefits rather than implementation mechanics.
These reductions improve the manuscript's flow while emphasizing the key innovations and practical value of SeuratExtend.

I had these minor comments:
* "Moreover, mainstream scRNA-seq analysis tools are primarily developed for either the R or Python platforms, with additional options like Nextflow and Snakemake" — I suggest revising this sentence. The tools are developed in R or python languages, which I would not call platforms. I would reword that Nextflow and Snakemake are workflow management systems that provide additional options for pipeline automation

RESPONSE:
We thank the reviewer for this terminological correction. We have revised this to "R or Python languages" and clarified that Nextflow and Snakemake are workflow management systems rather than programming languages. The sentence now reads: "Moreover, mainstream scRNA-seq analysis tools are primarily developed in either the R or Python languages, with additional options like Nextflow and Snakemake as workflow management systems."

* "the R ecosystem surrounding Seurat appears relatively limited" — I'm not sure I would agree with this. I counted wrappers for 17 methods currently. Yes it is true that there are more packages in scverse. However, I suggest moderating your claims about Seurat being limited.

RESPONSE:
We thank the reviewer for highlighting the substantial contributions of SeuratWrappers with its 17 methods and the broader value of tools in the R ecosystem. We have moderated our claims to acknowledge these resources while maintaining our point about integration frameworks. The revised sentence now states: "In contrast, the R ecosystem, despite having valuable resources like SeuratWrappers with its 17 methods, lacks a similarly comprehensive integration framework."

* Suggest removing snakemake from Table 1 — it is really different from the other tools listed there

RESPONSE:
We appreciate this insightful observation regarding Snakemake's placement in Table 1. We agree that

Snakemake, as a workflow management system rather than a direct single-cell analysis tool, is conceptually different from the other software packages in the comparison. Following your suggestion, we have removed Snakemake from Table 1 to maintain consistency in the comparison between software packages specifically designed for single-cell analysis. We have also adjusted the corresponding text in the manuscript to reflect this change, ensuring that our comparisons focus on tools with similar primary functions while still acknowledging workflow management systems in our broader discussion of the ecosystem.

Cookie Preference Center

We use cookies which are necessary to make our site work. We may also use additional cookies to analyze, improve and personalize our content and your digital experience. For more information, see our Cookie Policy.

You may choose not to allow some types of cookies. However, blocking some types may impact your experience of our site and the services we are able to offer. See the different category headings below to find out more or change your settings.

Allow all

Manage Consent Preferences

Strictly Necessary Cookies

Always active

These cookies are necessary for the website to function and cannot be switched off in our systems. They are usually only set in response to actions made by you which amount to a request for services, such as setting your privacy preferences, logging in or filling in forms. You can set your browser to block or alert you about these cookies, but some parts of the site will not then work.

Functional Cookies

These cookies enable the website to provide enhanced functionality and personalisation. They may be set by us or by third party providers whose services we

have added to our pages. If you do not allow these cookies then some or all of these services may not function properly.

Performance Cookies

These cookies allow us to count visits and traffic sources so we can measure and improve the performance of our site. They help us to know which pages are the most and least popular and see how visitors move around the site.

Targeting Cookies

These cookies may be set through our site by our advertising partners. They may be used by those companies to build a profile of your interests and show you relevant adverts on other sites. If you do not allow these cookies, you will experience less targeted advertising.

Cookie List

Clear

Apply Cancel
Consent Leg.Interest

Confirm my choices

Powered by onetrust
