# Supplementary material for: SeuratExtend: streamlining single-cell RNA-seq analysis through an integrated and intuitive framework
Source: Gigascience. 2025 Jul 8;14:giaf076. doi: 10.1093/gigascience/giaf076 (PMC12236070; doi:10.1093/gigascience/giaf076)
Supplement: giaf076_Reviewer_1_Report_Original_Submission — Yu H. Sun, Ph.D. -- 1/25/2025 [file giaf076_reviewer_1_report_original_submission.pdf]

Reviewer Report

Title: SeuratExtend: Streamlining Single-Cell RNA-Seq Analysis Through an Integrated and Intuitive Framework

Version: Original Submission **Date:** 1/25/2025

Reviewer name: Yu H. Sun, Ph.D.

Reviewer Comments to Author:

This manuscript introduces an extended version of the widely-used Seurat package, named SeuratExtend. Specifically, Hua et al. developed an integrated an intuitive framework to streamline scRNA-seq data analysis, such as trajectory analysis, GRN construction, and functional enrichment analysis. The package also features direct integration with other popular tools, including Seurat, scVelo, etc. Notably, the software has been demonstrated through training programs, with over 100 stars on GitHub, which is impressive. I have tested the package, including installation and some basic functions. Moreover, the GitHub webpage is well-documented, featuring multiple use cases tailored for beginners. The overall user experience exceeded my expectations, though I have a few minor comments for improvement:1, The DimPlot2 function is very useful, and easy to customize the colors. However, the default color scheme seems to be too dark. Considering a more distinguishable and visually appealing color palette might be a solution.2, How to control the angles of cell type labels when using VlnPlot2? The 'Split visualization' has all the labels in a horizontal direction, leading to overlapping in some cases, while 'Subset Analysis' plots have labels in 45 degree, which is much better to read. However, I didn't see a parameter to control this. Does VlnPlot2 handle this automatically?3, It's a very nice feature to have the 'Statistical Analysis' function to label significant groups. However, in single cell analysis, the p values are easy to be inflated due to the large number of cells. While the example pmbc data is relatively small, larger datasets might yield significant p values without obvious differences in the violin plots. It would be beneficial to mention this in the documentation, and provide some guidance so the results won't be misleading.4, The ClusterDistrBar is another valuable function. Based on my experience with similar analyses, I suggest incorporating features to identify robust changes in cell type composition. For instance, tools like sccomp can help determine changes in cell population composition.5, I wonder if the gene label directions can be changed easily for WaterfallPlot?6, Regarding the volcano plot, does LogFC mean log2 or log(e)? I noticed that this may not be consistent if you used different tools. For example, some tools like Seurat FindMarkers uses Log2, while NEBULA uses Log(e). Clear labeling on the x-axis and tutorial guidance would help ensure consistency.7, Very nice introduction about the color palettes at the end of the Enhanced Visualization tutorial.8, The incorporation of python tools into R is innovative, including scVelo, Palantir. There may be a need to continue incorporating new tools, such as Dynamo, a newer tool I started to use recently. While this is not required for the current revision, it could be a valuable direction for future development.Overall, this tool represents a comprehensive extension of Seurat, combining enhanced visualization, pathway enrichment, and trajectory analysis into a single package. I look forward to seeing a revised version of this manuscript.

Level of Interest

Please indicate how interesting you found the manuscript: Choose an item.

Quality of Written English

Please indicate the quality of language in the manuscript: Choose an item.

Declaration of Competing Interests

Please complete a declaration of competing interests, considering the following questions:

- Have you in the past five years received reimbursements, fees, funding, or salary from an organisation that may in any way gain or lose financially from the publication of this manuscript, either now or in the future?
- Do you hold any stocks or shares in an organisation that may in any way gain or lose financially from the publication of this manuscript, either now or in the future?
- Do you hold or are you currently applying for any patents relating to the content of the manuscript?
- Have you received reimbursements, fees, funding, or salary from an organization that holds or has applied for patents relating to the content of the manuscript?
- Do you have any other financial competing interests?
- Do you have any non-financial competing interests in relation to this paper?

If you can answer no to all of the above, write 'I declare that I have no competing interests' below. If your reply is yes to any, please give details below.

I declare no competing interests.

I agree to the open peer review policy of the journal. I understand that my name will be included on my report to the authors and, if the manuscript is accepted for publication, my named report including any attachments I upload will be posted on the website along with the authors' responses. I agree for my report to be made available under an Open Access Creative Commons CC-BY license (http://creativecommons.org/licenses/by/4.0/). I understand that any comments which I do not wish to be included in my named report can be included as confidential comments to the editors, which will not be published.

Choose an item.

To further support our reviewers, we have joined with Publons, where you can gain additional credit to further highlight your hard work (see: https://publons.com/journal/530/gigascience). On publication of

this paper, your review will be automatically added to Publons, you can then choose whether or not to claim your Publons credit. I understand this statement.

Yes Choose an item.
