# Supplementary material for: SeuratExtend: streamlining single-cell RNA-seq analysis through an integrated and intuitive framework
Source: Gigascience. 2025 Jul 8;14:giaf076. doi: 10.1093/gigascience/giaf076 (PMC12236070; doi:10.1093/gigascience/giaf076)
Supplement: giaf076_Reviewer_2_Report_Original_Submission — Daniel A. Skelly -- 2/16/2025 [file giaf076_reviewer_2_report_original_submission.pdf]

Reviewer Report

Title: SeuratExtend: Streamlining Single-Cell RNA-Seq Analysis Through an Integrated and Intuitive Framework

Version: Original Submission **Date:** 2/16/2025

Reviewer name: Daniel A. Skelly

Reviewer Comments to Author:

Overall, this is a very nice writeup of a useful package that extends the Seurat package to expand possibilities for single cell analysts in R. I liked the visualization options, the ability to try certain python-based tools easily in R which was not previously easy, and some of the authors' new innovations like their use of pathway enrichment scores in broad ways. Kudos to the authors for releasing a package with really excellent documentation and tutorials!I think this paper could be made better if the authors stressed with a little more clarity how specifically their work is innovative. The text in the present manuscript is fine but reads like a bit of a grab bag of functionality. For example, from the abstract:"SeuratExtend offers a user-friendly and intuitive interface for performing a wide range of analyses, including functional enrichment, trajectory inference, gene regulatory network reconstruction, and denoising. The package integrates multiple databases, … and incorporates popular Python tools … [We] showcase its novel applications in pathway-level analysis and cluster annotation. SeuratExtend enhances data visualization …"How could they be more clear or specific? One example could be by categorizing what SeuratExtend can do that other packages can't. For example, I see innovations in perhaps three general areas:1. Making single cell analyses easier/faster/prettier (i.e. visualizations, pathway enrichment)2. Making previously published single cell tools more broadly accessible (e.g. first option to bring certain python tools to R)3. New innovations (e.g. dimensionality reduction and clustering based on pathway enrichment scores; may not be completely new but I don't recall seeing this elsewhere)If this was added I feel the paper would more clearly communicate to readers the information necessary for them to choose whether they want to try the package.I have the following additional significant comments:* Integration of multiple databases for GSEA â€” these methods are good, but what about in a few years when those databases have been updated? Do the authors intend to continue updating? Could they provide a function for users to use their own database (e.g. .gaf and .obo files, for example for another model organism)? Similar comment about gene identifer conversion, which may need to be updated every few years.* "While the Python ecosystem has benefited greatly from the comprehensive scverse project [7], which utilizes the universal AnnData format to connect various tools and algorithms, a comparable integrated solution has been lacking in the R community. SeuratExtend addresses this gap by providing a unified framework centered around the Seurat object, effectively becoming the R counterpart to scverse." â€”> some might argue that SeuratWrappers is this solution. The authors should more clearly and explicitly comment on what SeuratExtend does differently/better than SeuratWrappers.* I'm not particularly convinced by the authors' example studies that used SeuratExtend. For example, they describe Hua-Vella et al. (2022) and Hua et al. (2023). These are very nice studies and I have no doubt they made use of SeuratExtend in their analyses. But I don't

see anything these authors describe those authors doing as being uniquely possible with SeuratExtend. Perhaps SeuratExtend made their analyses easier, or faster. But it would be better if we had some further concrete details. For example, something communicating a message like one of the following: (1) the authors only tested method X on a whim because it was so easy to run in SeuratExtend, and found that it revealed unexpected biology Y; or (2) the authors were able to bring together method X which runs in R and method Y which runs in python and the joint inference â€” not possible in other packages â€” revealed key result Z. If the authors of this manuscript can't point to those sorts of examples, then I'm not sure it adds much to include this discussion in the present paper.* I really liked the section "Novel Applications of SeuratExtend in Pathway-Level Analysis and Cluster Annotation", especially "Exploring and Analyzing Single-Cell Data at the Pathway Level". I thought these applications could perhaps be stressed a bit more strongly or made more prominent earlier in the paper.* Figures 2 and 3 are showing example plots from which we don't actually need to infer any important biology. I thought these figures could be combined and each individual plot type only shown once. (This is for clarity and I don't see anything incorrect about the authors' current plots.* There may be some issues with dependencies for some users. For example, it prompted me to install viridis and loomR as I went through the Quickstart. I ended up encountering an error `there is no package called 'loomR'` while trying. I had to manually install with `remotes::install_github(repo = "mojaveazure/loomR")`. Maybe provide an explicit dependencies list/list of recommended packages to install?* I had an error the first time calling Palantir.RunDM(). I hadn't created a seuratextend environment. I found that I could do this manually using `create_condaenv_seuratextend()`, but that this wasn't supported for Apple Silicon chips. I would suggest that the authors do try to find a way to get this working on newer Apple chips, because Mac machines are very common among bioinformaticians in my experience.* While the writing is largely quite clear, I found it to be a bit voluminous. If the authors are able to cut down on text length that may help in emphasizing the key points that make their package valuable to users.I had these minor comments:* "Moreover, mainstream scRNA-seq analysis tools are primarily developed for either the R or Python platforms, with additional options like Nextflow and Snakemake" â€” I suggest revising this sentence. The tools are developed in R or python languages, which I would not call platforms. I would reword that Nextflow and Snakemake are workflow management systems that provide additional options for pipeline automation* "the R ecosystem surrounding Seurat appears relatively limited" â€” I'm not sure I would agree with this. I counted wrappers for 17 methods currently. Yes it is true that there are more packages in scverse. However, I suggest moderating your claims about Seurat being limited.* Suggest removing snakemake from Table 1 â€” it is really different from the other tools listed there

Level of Interest

Please indicate how interesting you found the manuscript: Choose an item.

Quality of Written English

Please indicate the quality of language in the manuscript: Choose an item.

Declaration of Competing Interests

Please complete a declaration of competing interests, considering the following questions:

- Have you in the past five years received reimbursements, fees, funding, or salary from an organisation that may in any way gain or lose financially from the publication of this manuscript, either now or in the future?
- Do you hold any stocks or shares in an organisation that may in any way gain or lose financially from the publication of this manuscript, either now or in the future?
- Do you hold or are you currently applying for any patents relating to the content of the manuscript?
- Have you received reimbursements, fees, funding, or salary from an organization that holds or has applied for patents relating to the content of the manuscript?
- Do you have any other financial competing interests?
- Do you have any non-financial competing interests in relation to this paper?

If you can answer no to all of the above, write 'I declare that I have no competing interests' below. If your reply is yes to any, please give details below.

I declare that I have no competing interests

I agree to the open peer review policy of the journal. I understand that my name will be included on my report to the authors and, if the manuscript is accepted for publication, my named report including any attachments I upload will be posted on the website along with the authors' responses. I agree for my report to be made available under an Open Access Creative Commons CC-BY license (http://creativecommons.org/licenses/by/4.0/). I understand that any comments which I do not wish to be included in my named report can be included as confidential comments to the editors, which will not be published.

Choose an item.

To further support our reviewers, we have joined with Publons, where you can gain additional credit to further highlight your hard work (see: https://publons.com/journal/530/gigascience). On publication of this paper, your review will be automatically added to Publons, you can then choose whether or not to claim your Publons credit. I understand this statement.

Yes Choose an item.
